# The effectiveness of ablative and non‐surgical therapies for early hepatocellular carcinoma: Systematic review and network meta‐analysis of randomised controlled trials

**DOI:** 10.1002/cam4.6643

**Published:** 2023-10-30

**Authors:** Emily South, Ros Wade, Sumayya Anwer, Sahar Sharif‐Hurst, Melissa Harden, Helen Fulbright, Sofia Dias, Mark Simmonds, Ian Rowe, Patricia Thornton, Tze Min Wah, Alison Eastwood

**Affiliations:** ^1^ Centre for Reviews and Dissemination University of York York UK; ^2^ Leeds Teaching Hospitals NHS Trust Leeds UK; ^3^ Patient Collaborator UK

**Keywords:** hepatocellular carcinoma, microwave ablation, network meta‐analysis, percutaneous ethanol injection, radiofrequency ablation, systematic review

## Abstract

**Background & Aims:**

Non‐surgical therapies are frequently used for patients with early or very early hepatocellular carcinoma (HCC). The aim of this systematic review and network meta‐analysis (NMA) was to evaluate and compare the effectiveness of ablative and non‐surgical therapies for patients with small HCC.

**Methods:**

Nine databases were searched (March 2021) along with clinical trial registries. Randomised controlled trials (RCTs) of any ablative or non‐surgical therapy versus any comparator in patients with HCC ≤3 cm were eligible. Risk of bias (RoB) was assessed using the Cochrane RoB 2 tool. The effectiveness of therapies was compared using NMA. Threshold analysis was undertaken to identify which NMA results had less robust evidence.

**Results:**

Thirty‐seven eligible RCTs were included (including over 3700 patients). Most were from China (*n* = 17) or Japan (*n* = 7). Sample sizes ranged from 30 to 308 patients. The majority had a high RoB or some RoB concerns. No RCTs were identified for some therapies and no RCTs reported quality of life outcomes. The results of the NMA and treatment effectiveness rankings were very uncertain. However, the evidence demonstrated that percutaneous ethanol injection was worse than radiofrequency ablation for overall survival (hazard ratio [HR]: 1.45, 95% credible interval [CrI]: 1.16–1.82), progression‐free survival (HR: 1.36, 95% CrI: 1.11–1.67), overall recurrence (relative risk [RR]: 1.19, 95% CrI: 1.02–1.39) and local recurrence (RR: 1.80, 95% CrI: 1.19–2.71). The threshold analysis suggested that robust evidence was lacking for some comparisons.

**Conclusions:**

It is unclear which treatment is most effective for patients with small HCC because of limitations in the evidence base. It is also not known how these treatments would impact on quality of life. Further high quality RCTs are needed to provide robust evidence but may be difficult to undertake.

## Graphical abstract

This systematic review and network meta‐analysis compared the effectiveness of ablative and non‐surgical therapies for patients with hepatocellular carcinoma (HCC) up to 3cm. Evidence from randomised controlled trials demonstrated that percutaneous ethanol injection was worse than radiofrequency ablation for overall survival, progression‐free survival, overall recurrence and local recurrence. However, overall the results of the NMA and treatment effectiveness rankings were very uncertain and it is unclear which treatment is most effective for patients with small HCC.

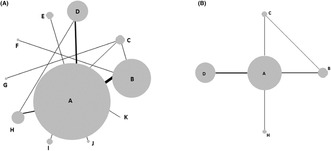

## 1 INTRODUCTION

Hepatocellular carcinoma (HCC) is the most common type of primary liver cancer, representing around 90% of cases.^1^ The global incidence of HCC increased by 75% between 1990 and 2015.^2^ Aetiology varies geographically but hepatitis B virus (HBV) and alcohol are the most common causes of primary liver cancer globally.^2^

Once HCC has become symptomatic, prognosis is poor,^3^ so early diagnosis is important. Ultrasound surveillance of patients with cirrhosis is recommended every 6 months to detect early HCC.^1^ Staging systems for HCC take account of tumour status, liver function (based on Child‐Pugh classification, bilirubin, albumin, clinically relevant portal hypertension, ascites etc.) and performance status.^1^ Very early stage HCC is defined by the Barcelona clinic liver cancer (BCLC) staging system as a single tumour ≤2 cm in diameter without vascular invasion or extrahepatic spread, preserved liver function and a performance status of 0. Early stage HCC is a single tumour of any size or 2–3 tumours ≤3 cm without macrovascular invasion or extrahepatic spread, preserved liver function, and a performance status of 0.^4^

If patients have good liver function, early HCC can be treated with curative intent. Treatment options include surgical interventions (liver resection or transplantation) and non‐surgical interventions, such as ablative therapies. With treatment, 5‐year survival rates for very early or early HCC are generally high.^1^ However, resection and transplantation are not available for all patients. Liver resection is restricted by factors including the presence and severity of portal hypertension, location of the tumour and liver dysfunction.^5^ Liver transplantation is limited by availability.^5^ Therefore, ablative therapies are frequently used in patients with early stage HCC, primarily microwave ablation (MWA) and radiofrequency ablation (RFA). Other ablative methods include percutaneous ethanol injection (PEI), percutaneous acetic acid injection (PAI), irreversible electroporation (IRE), laser ablation and cryoablation. Stereotactic ablative radiotherapy (SABR) is an emerging alternative to invasive ablation. Non‐ablative methods including transarterial (chemo‐) embolization (TA(C)E), and selective internal radiation therapy (SIRT) may also be used.

There has been no definitive assessment of all ablative and other non‐surgical therapies for this group of patients. Previous network meta analyses (NMAs) with relevant populations have focused on specific therapies and outcomes only.^6^, ^7^, ^8^ For example one recent NMA on treatments for solitary HCC ≤5 cm or multifocal HCC ≤3 cm assessed overall survival (OS) and recurrence‐free survival only (reporting very limited data on HCC ≤3 cm in a subgroup analysis).^6^ Cochrane reviews have been published on some of the therapies and have generally found very limited, low quality evidence.^9^, ^10^, ^11^, ^12^, ^13^

The aim of this systematic review and network meta‐analysis (NMA) was to evaluate and compare the effectiveness of all ablative and non‐surgical therapies for patients with small HCC (up to 3 cm). NMA allows the effectiveness of multiple interventions to be compared, using both direct and indirect evidence across a network of interventions.^14^ This means that pairs of interventions can be compared even if they have not been directly compared in a clinical trial. It also enables the relative rankings of different interventions to be estimated.^14^ This systematic review of randomised controlled trials (RCTs) was part of a broader National Institute for Health and Care Research‐funded project comparing the effectiveness of ablative and non‐surgical therapies for early and very early HCC.^15^

## 2 METHODS

A systematic review and NMA of RCTs was undertaken. The protocol was registered as PROSPERO CRD42020221357. A group of clinical experts and patient advisors provided advice throughout the project.

### 2.1 Search strategy

A comprehensive search strategy was developed by information specialists (MH and HF). Four databases (MEDLINE, Embase, CENTRAL, Science Citation Index) were searched for RCTs in February and March 2021. See Appendix S1 for the full search strategies. Searches were limited to articles published from 2000, based on clinical advice that practice and techniques have evolved over the past two decades. No language limits were applied. Four systematic review databases were also searched and the reference lists of any relevant systematic reviews were checked. The International HTA database was searched for any ongoing or completed health technology assessments. Clinical trial registries (ClinicalTrials.Gov and the European Union Clinical Trials Register) were searched in April 2021 to identify ongoing studies. Clinical advisors were consulted to identify any further relevant studies. Search results were imported into EndNote 20 (Clarivate Analytics) and deduplicated.

### 2.2 Study selection

RCTs that included patients with HCC up to 3 cm were eligible. A cut‐off of 3 cm was chosen to reflect the most appropriate population for ablative therapies given the technical limitations of some ablative methods. RCTs with wider populations that reported separate data for patients with HCC up to 3 cm were also included. Any ablative or non‐surgical therapy was eligible, including as follows:

1. Radiofrequency ablation (RFA)
2. Microwave ablation (MWA)
3. Laser ablation
4. High intensity focussed ultrasound (HIFU)
5. Cryoablation
6. Percutaneous ethanol injection (PEI)
7. Percutaneous acid injection (PAI)
8. Irreversible electroporation (IRE)
9. Transarterial chemoembolization (TACE)
10. Transarterial embolization (TAE)
11. Selective internal radiation therapy (SIRT; also known as transarterial radioembolization [TARE])
12. Electrochemotherapy (ECT)
13. Histotripsy
14. Stereotactic ablative radiotherapy (SABR)
15. Wider radiotherapy techniques

Any comparator was eligible, including surgical resection. However, RCTs that compared different methods of undertaking the same intervention were excluded. Eligible outcomes were OS, progression‐free survival (PFS), time to progression (TTP), serious adverse events, intervention‐specific adverse events and quality of life.

As the searches were expected to return a large number of records, titles and abstracts were screened by a single reviewer (RW, SS‐H or ES), with 10% checked by a second reviewer. Any disagreements were discussed and there were no major discrepancies between reviewers' screening decisions. Full texts of potentially eligible papers were retrieved and screened for inclusion by two reviewers independently (RW or SS‐H). Disagreements were resolved through discussion or consultation with a third reviewer (AE). When necessary, authors of conference abstracts were contacted for further information to enable screening and data extraction.

### 2.3 Data extraction and quality assessment

A data extraction form was developed and piloted using Microsoft Excel®. Data extracted included intervention and comparator details, patient characteristics and aggregate results. Hazard ratios (HRs) were extracted for survival outcomes and relative risks (RRs) for dichotomous outcomes, in addition to their corresponding 95% confidence intervals or standard errors. Kaplan–Meier data were extracted and used to compute hazard ratios and variances for studies where these were not reported. Outcomes were extracted as reported in the primary studies (see Table A1 in the Appendix S2 for the outcomes reported by each study). Data were extracted from conference abstracts if authors did not respond to the request for further information.

Risk of bias (RoB) was assessed using the Cochrane RoB 2 tool.^16^ Data extraction and quality assessment were both undertaken by one reviewer (ES or SS‐H) and checked by a second reviewer (RW). The second reviewer ensured that RoB decisions were made consistently across all studies. Discrepancies were resolved through discussion. For foreign language studies, data extraction and quality assessment were undertaken by a native speaker.

### 2.4 Synthesis

All analyses were carried out in R (Version 4.1.2).^17^ NMAs were conducted for four outcomes: OS, PFS, overall recurrence and local recurrence. For the PFS outcome, trials which reported outcomes of recurrence‐free survival, disease‐free survival, cancer‐free survival or event‐free survival were also included in the NMA. For the local recurrence outcome, trials which reported outcomes of local tumour progression or local disease progression were also included in the NMA. The analyses were conducted in a Bayesian framework using Markov chain Monte Carlo (MCMC) methods using the GeMTC package.^18^ All four outcomes were modelled using a normal likelihood with an identity link.^19^ Fixed (common) and random effects contrast‐based models,^19^, ^20^, ^21^ which appropriately account for correlations in trials with more than two arms, were used. Models were sampled for 100,000 iterations over 4 chains after an initial burn‐in of 50,000 iterations. Model convergence was assessed through visual inspection of Brook‐Gelman‐Rubin diagnostic and history plots.^22^ The DIC was used to choose between the fixed‐effect and random‐effects model; if the difference between the two models was less than three, the simpler fixed‐effect model was selected. Inconsistency was checked by comparing the model fit and between‐study heterogeneity from the NMA models to the corresponding unrelated mean effects (inconsistency) models.^20^, ^23^ See the full report for further details on the NMA methods.^15^ As some studies and outcomes could not be included in the NMAs, data were also narratively synthesised.

Threshold analysis is a novel statistical approach used to examine which NMA results could plausibly change due to potential changes in the observed evidence (e.g. due to bias or sampling variation).^24^, ^25^ Threshold analysis was conducted at the contrast (treatment effect) level using the nmathresh package in R (Version 0.1.6)^24^ to identify which comparisons had less robust evidence and would benefit from further trials.

### 2.5 Patient and public involvement

A patient collaborator (PT) was involved throughout the project, from developing the proposal to disseminating the findings. Four additional patients were recruited to the project advisory group, providing input at key stages of the project, including protocol development and interpretation of review findings.

## 3 RESULTS

**FIGURE 1:**
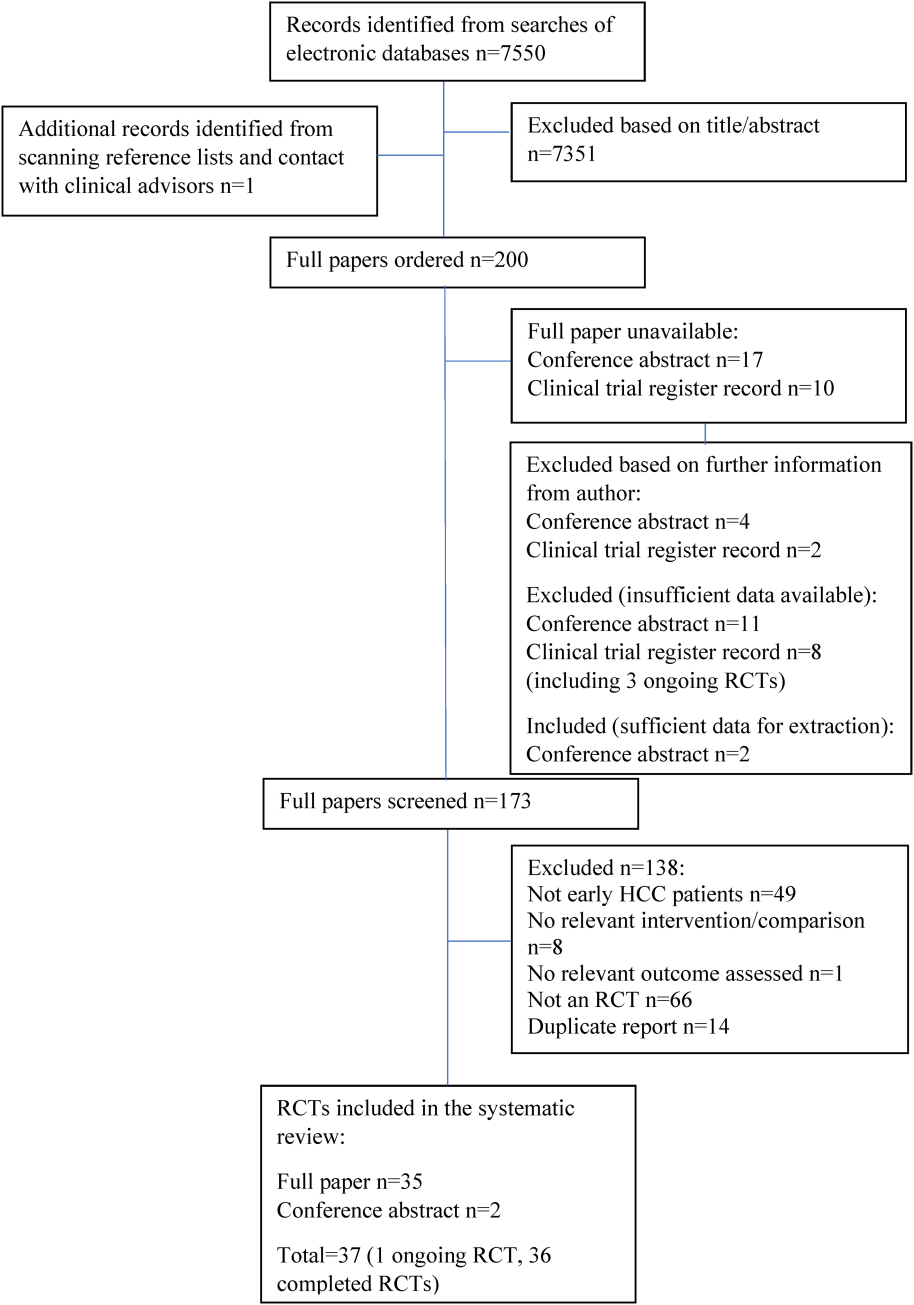
Flow diagram of the study selection process. RCT, randomised controlled trial.

The database and clinical trial register searches identified 7550 unique records. One additional study was identified from systematic review reference lists. Two hundred records were considered potentially eligible and full texts were ordered. In total, 37 eligible RCTs were identified. This included one protocol for an ongoing RCT with no published results.^26^ Two RCTs were published as conference abstracts only.^27^, ^28^ Figure 1 shows the study selection process. Detailed study characteristics and results are reported in Appendix S1 Table A1.

**FIGURE 2:**
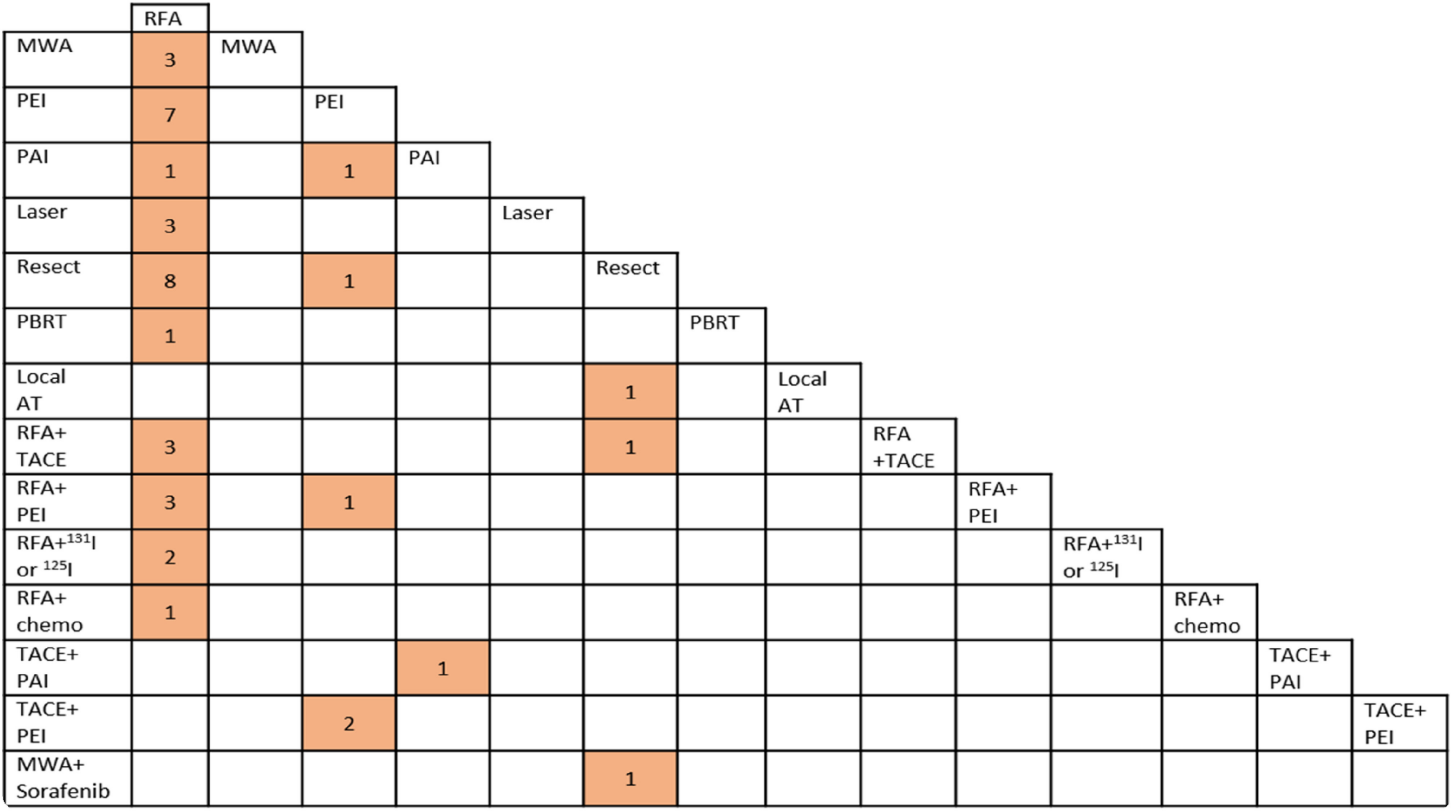
Matrix of RCT comparisons. AT, ablative therapy; Chemo, chemotherapy; MWA, microwave ablation; PAI, percutaneous acid injection; PBRT, proton beam radiotherapy; PEI, percutaneous ethanol injection; resect, resection; RFA, radiofrequency ablation; TACE, transarterial chemoembolization.

The majority of studies were conducted in Asian countries (China [*n* = 17], Japan [*n* = 7], Taiwan [*n* = 4] or South Korea [*n* = 1]). Sample sizes were generally small, ranging from 30 to 308 patients. Fifteen of the RCTs only included patients with HCC up to 3 cm in diameter.^27^, ^28^, ^29^, ^30^, ^31^, ^32^, ^33^, ^34^, ^35^, ^36^, ^37^, ^38^, ^39^, ^40^, ^41^ The remaining RCTs either reported separate results for a subgroup of patients with small HCC or a clear majority of participants had tumours under 3 cm. RFA was the most frequently assessed therapy, either alone or combined with other therapies (see Figure 2). Outcomes reported included OS, PFS, recurrence, response and adverse events. One study reported patient satisfaction^41^ but no studies reported quality of life outcomes. No RCTs were identified which assessed HIFU, cryoablation, IRE, ECT, histotripsy, SABR or wider radiotherapy techniques. Clinical trial register records for a further ongoing four RCTs that would meet our eligibility criteria were found (see Appendix S3 for details).

The quality of the included studies was mixed and reporting of methods was generally poor (see Appendix S1 Figure A1). The overall RoB judgement was high for 12 RCTs, low for 9 RCTs and there were some concerns for 14 RCTs. One ongoing RCT^26^ and one that did not report relevant outcomes for the eligible subgroup^42^ were not assessed.

As it was not possible to include all results in the NMA (see section on Results of NMA for further details), study findings were also narratively synthesised. For the following comparisons, a single RCT was identified: RFA versus PAI; RFA versus proton beam therapy; RFA versus RFA + Iodine‐131; RFA versus RFA + Iodine‐125; RFA versus RFA + chemotherapy; RFA + TACE versus resection; PEI versus PAI; PEI vs. resection; PEI versus RFA + PEI; PAI versus PAI + TACE; percutaneous local ablative therapy versus resection; microwave ablation + sorafenib versus resection. For the comparisons with a single RCT, study results have not been summarised in the narrative synthesis below but extracted results can be found in Appendix S1 Table A1 (or see the full report for a summary of these studies^15^).

### 3.1 Narrative synthesis

Seven RCTs compared RFA with PEI.^29^, ^33^, ^37^, ^39^, ^43^, ^44^, ^45^ RFA appeared to be more effective than PEI in most studies that reported OS (4/6 RCTs favoured RFA), event‐ or cancer‐free survival (3/3 RCTs) and recurrence or local tumour progression (5/6 RCTs); all studies favouring RFA had a low RoB or some RoB concerns. However, some studies reported worse adverse events after treatment with RFA.^37^, ^39^, ^43^, ^45^

Seven completed RCTs compared RFA with resection, with mixed results.^28^, ^31^, ^42^, ^46^, ^47^, ^48^, ^49^ Two RCTs reported better 5‐year OS after resection (one high ROB, one low ROB),^47^, ^49^ one reported slightly better 3‐year OS after RFA (with some RoB concerns)^31^ and one reported similar rates between groups (low RoB).^48^ Disease or recurrence‐free survival results were also mixed.^28^, ^31^, ^48^, ^49^ One study reported that adverse events (including major complications) were more frequent after resection than RFA.^31^

Whilst three RCTs compared RFA with MWA,^50^, ^51^, ^52^ only one (with some RoB concerns) reported survival and recurrence outcomes. OS was similar between groups at 2 years.^51^ In the RFA group, more patients experienced recurrence but the median time‐to‐progression was longer. There were more major (or Grade 4) complications after MWA in two studies,^51^, ^52^ but there were more Grade 1–3 adverse events after RFA.^51^ Only one of the three small RCTs comparing RFA with laser ablation (all with some RoB concerns)^41^, ^53^, ^54^ reported survival or progression. PFS and local disease progression were better after RFA than laser ablation.^53^ However, one RCT reported that patient satisfaction was higher after laser ablation.^41^ Results on adverse events were mixed.

RCTs assessing RFA in combination with other treatments were limited by small sample sizes and most had a high RoB or some RoB concerns. Three RCTs compared RFA with RFA + TACE.^27^, ^38^, ^55^ RFA + TACE was generally superior to RFA alone in terms of survival and progression/recurrence‐free survival, although there were some inconsistencies in results. Rates of major complications were the same between treatment groups.^27^, ^38^ Three RCTs compared RFA with RFA + PEI.^44^, ^56^, ^57^ RFA + PEI was superior in terms of OS^56^, ^57^ and recurrence.^56^

Results from two small RCTs comparing PEI + TACE with PEI alone were inconsistent. One RCT with a low RoB favoured PEI + TACE for OS and recurrence^36^ and the other RCT (with some RoB concerns) favoured PEI alone for OS and recurrence (but the PEI + TACE group had a longer mean cancer‐free survival).^58^ The RCT with a low RoB reported more major complications in the combined treatment group.^36^

### 3.2 Results of NMA

**FIGURE 3:**
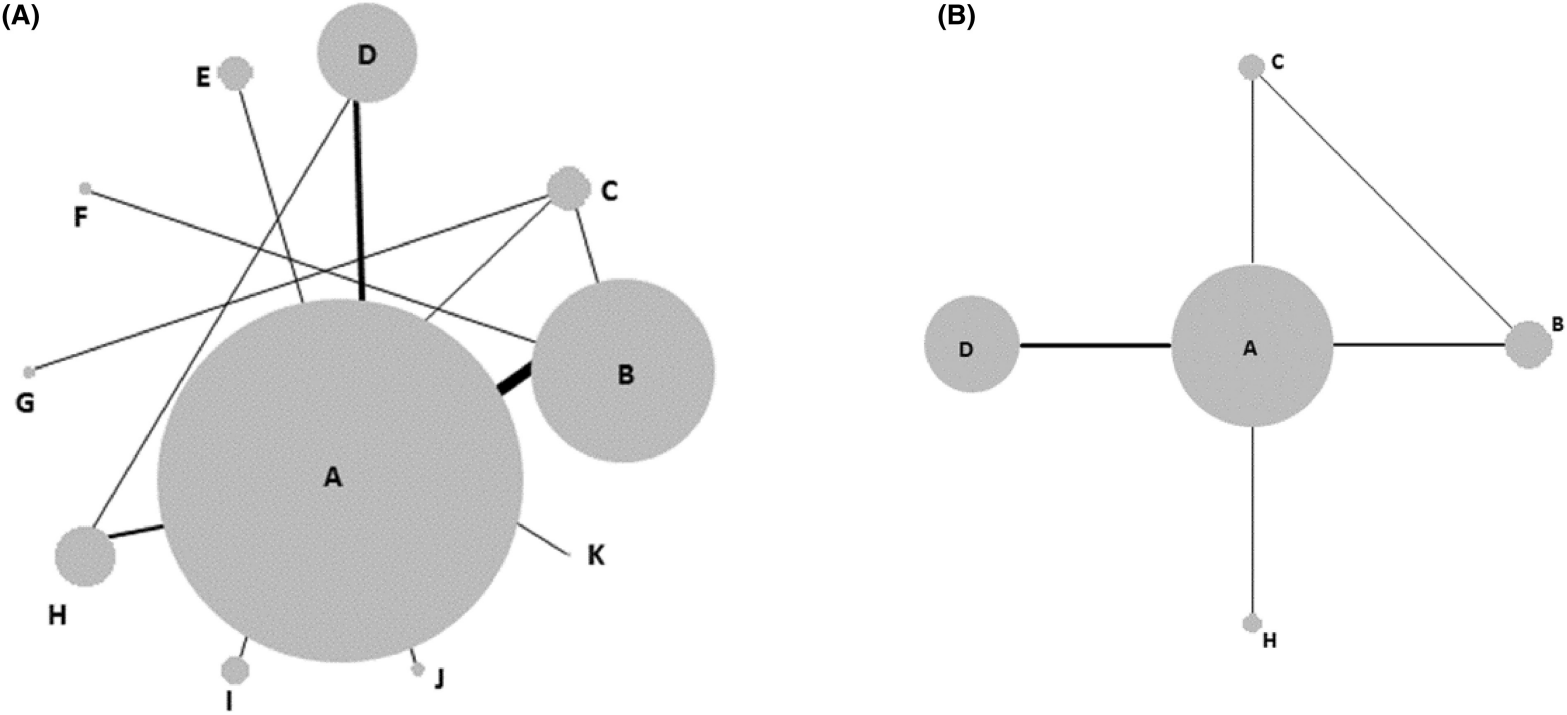
Network diagrams for survival outcomes: (A) overall survival (B) progression‐free survival. Nodes are sized proportional to the number of patients who received the relevant treatment; lines are weighted according to the number of studies that reported the relevant comparison. Treatment Codes: (A) radiofrequency ablation (RFA), (B): percutaneous ethanol injection (PEI), (C): percutaneous acid injection (PAI), (D): resection, E: microwave ablation (MWA), (F): transarterial chemoembolization (TACE) + PEI, (G): TACE + PAI, (H:) RFA+ TACE, (I): RFA + Iodine‐125, (J): RFA + PEI, (K): laser ablation.

**FIGURE 4:**
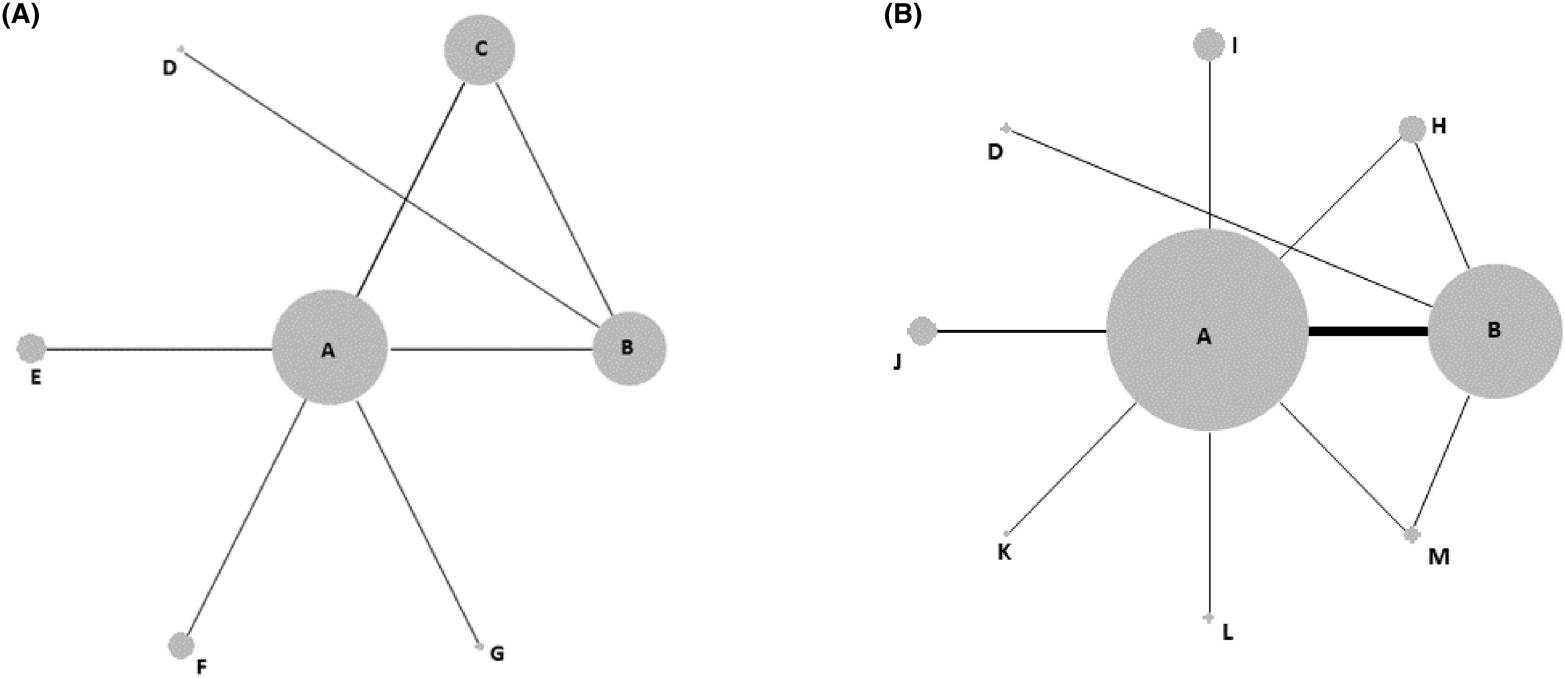
Network diagrams for recurrence outcomes: (A) overall recurrence (B) local recurrence. Nodes are sized proportional to the number of patients who received the relevant treatment; lines are weighted according to the number of studies that reported the relevant comparison. Treatment Codes: A: radiofrequency ablation (RFA), B: percutaneous ethanol injection (PEI), C: resection, D: transarterial chemoembolization (TACE) + PEI, E: RFA + Iodine‐125, F: microwave (MWA) + sorafenib, G: RFA + systemic chemotherapy, H: percutaneous acid injection (PAI), I: MWA, J: RFA + TACE, K: Laser, L: RFA + PEI, M: high‐dose PEI

Network diagrams for OS, PFS, overall recurrence and local recurrence are shown in Figures 3 and 4. Not all of the RCTs identified in the systematic review could be included in the NMAs. Some did not report data on any of the four outcomes for the ≤3 cm tumour subgroup, and some reported data that were not suitable for inclusion (e.g. would have required strong assumptions to be made). There were also three RCTs that included patients with recurrent or residual HCC^35^, ^48^, ^55^; clinical experts advised that it was not appropriate to synthesise these results with those for non‐recurrent HCC patients.

For all four outcomes, the fixed‐effect model was chosen (see Appendix S1 Table A2 for model fit parameters). The results of the random‐effects model can be found in the full report.^15^ There was no evidence to suggest inconsistency in the networks for OS or overall recurrence (see Appendix S5 [Figures A2 and A3, Tables A3 and A4] for inconsistency checks). There was no potential for inconsistency to be detected in the networks for PFS and local recurrence as there was no independent, indirect evidence for any of the comparisons.

**FIGURE 5:**
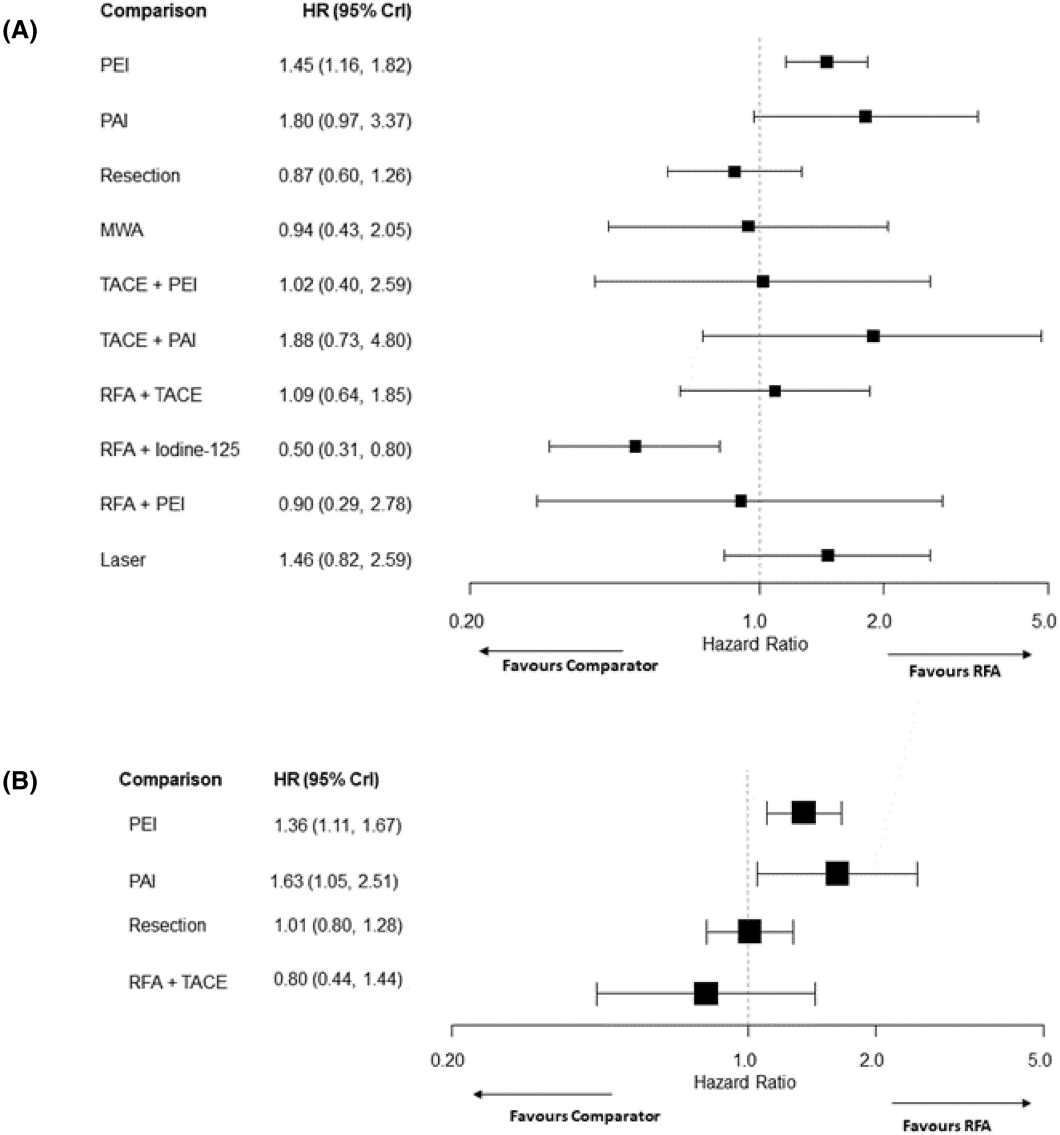
Forest plots for survival outcomes comparing interventions to RFA, (A) overall survival, (B) progression‐free survival. CrI, credible interval; HR, hazard ratio; MWA, microwave ablation; PEI, percutaneous ethanol injection; PAI, percutaneous acid injection; RFA, radiofrequency ablation; TACE, transarterial chemoembolization.

**FIGURE 6:**
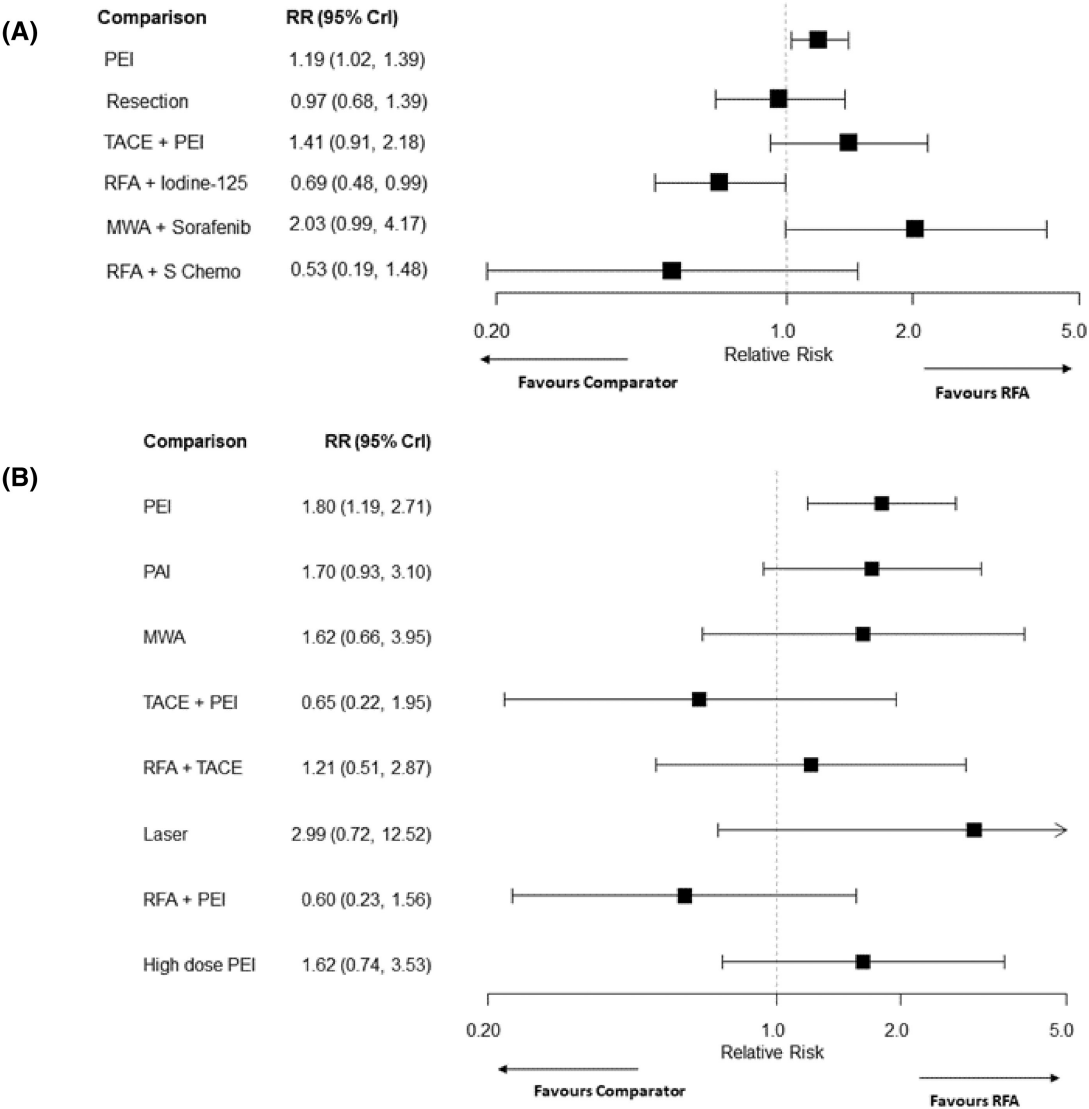
Forest plots for recurrence outcomes comparing interventions to RFA, (A) overall recurrence (B) local recurrence. CrI, credible interval; MWA, microwave ablation; PEI, percutaneous ethanol injection; PAI, percutaneous acid injection; RFA, radiofrequency ablation; RR, relative risk; TACE, transarterial chemoembolization; S Chemo, systemic chemotherapy.

The forest plots in Figures 5 and 6 show the results for all comparisons involving RFA. The HRs and RRs for all other comparisons are reported in Tables A5–A8 in the Appendix S6. Treatment rankings for all outcomes were very uncertain, with all treatments displaying wide credible intervals for the ranks for PFS, overall recurrence and local recurrence (see Appendix S7 Tables A9–A12).

Nodes are sized proportional to the number of patients who received the relevant treatment; lines are weighted according to the number of studies that reported the relevant comparison. Treatment Codes: (A): radiofrequency ablation (RFA), (B): percutaneous ethanol injection (PEI), (C): resection, (D): transarterial chemoembolization (TACE) + PEI, E: RFA + Iodine‐125, (F): microwave (MWA) + sorafenib, (G): RFA + systemic chemotherapy, (H): percutaneous acid injection (PAI), (I): MWA, (J): RFA + TACE, (K): Laser, (L): RFA + PEI, (M): high‐dose PEI.

### 3.3 Overall survival

Data from 16 RCTs^27^, ^29^, ^30^, ^31^, ^33^, ^36^, ^37^, ^38^, ^39^, ^47^, ^49^, ^51^, ^54^, ^57^, ^59^, ^60^ (fifteen two‐arm and one three‐arm trials) comparing 11 interventions were included in the NMA of OS. Results for many comparisons were uncertain and included the ‘null’ effect. However, there was evidence that PEI was associated with worse OS compared to RFA (HR: 1.45, 95% credible interval (CrI): 1.16–1.82) and RFA + Iodine‐125 was superior to RFA alone (HR: 0.50, 95% CrI: 0.31–0.80) (see Figure 5). There was also evidence that surgical resection improved OS compared to PEI (HR: 0.60, 95% CrI: 0.39–0.92) and that RFA + Iodine‐125 was superior to PEI, PAI, TACE + PAI, RFA + TACE and laser ablation (see Appendix S6 Table A5). While RFA + Iodine‐125 had the highest probability of being ranked the best treatment, all other treatments had very wide credible intervals with some 95% credible intervals including all 11 potential ranks (see Appendix S7 Table A9).

### 3.4 Progression‐free survival

Six RCTs^28^, ^31^, ^37^, ^38^, ^45^, ^49^ (five two‐arm and one three‐arm trial) comparing six interventions were included in the NMA of PFS. NMA results suggested that PEI (HR: 1.36, 95% CrI: 1.11–1.67) and PAI (HR: 1.63, 95% CrI:1.05–2.51) worsened PFS compared with RFA (see Figure 5). There was insufficient evidence of any difference between other treatments in the network. RFA + TACE had the highest probability of being ranked the best treatment, closely followed by RFA and resection (see Appendix S7 Table A10).

### 3.5 Overall recurrence

Seven RCTs^30^, ^31^, ^32^, ^34^, ^39^, ^40^, ^58^ comparing seven interventions were included in the NMA of overall recurrence. All included RCTs had two treatment arms. The NMA results suggested that there was a higher risk of overall recurrence after treatment with PEI than after treatment with RFA (RR: 1.19, 95% CrI: 1.02–1.39) (see Figure 6). RFA + Iodine‐125 decreased the risk of recurrence compared with RFA (RR: 0.69, 95% CrI: 0.48–0.99). However, the 95% credible intervals for these estimates are very close to the ‘null’ effect. The evidence also suggested that there was a lower risk of overall recurrence with RFA + Iodine‐125 compared with both PEI and TACE + PEI (see Appendix S6 Table A7). MWA + Sorafenib was inferior to resection, RFA + Iodine‐125 and RFA + systemic chemotherapy. There was insufficient evidence to suggest any difference between treatments for the other comparisons. RFA + systemic chemotherapy and RFA + Iodine‐125 had the highest probabilities of being ranked the best treatment (see Appendix S7 Table A11).

### 3.6 Local recurrence

Ten RCTs^27^, ^33^, ^36^, ^37^, ^38^, ^43^, ^45^, ^51^, ^53^, ^56^ (eight two‐arm and two three‐arm trials), comparing nine different interventions, were included in the NMA of local recurrence. Evidence from this NMA suggested that there was a greater risk of local recurrence after PEI than after RFA (RR: 1.80, 95% CrI: 1.19–2.71) (see Figure 6). RFA + PEI was also superior to PEI alone (RR: 0.33, 95% CrI: 0.12–0.94) (see Appendix S6 Table A8). There was insufficient evidence to suggest any difference for the other comparisons. RFA + PEI had the highest probability of being ranked the best treatment (see Appendix S7 Table A12) but the probability was below 50%.

### 3.7 Threshold analysis

The threshold analysis suggested a lack of robust evidence for some comparisons. For example, for the NMA of OS, the result for the MWA versus RFA comparison was sensitive to uncertainty in the data and could plausibly be changed by additional evidence. The forest plot for the OS threshold analysis is presented in Appendix S8 Figure A4. See the full report for further details and results of the threshold analysis.^15^

## 4 DISCUSSION

Thirty‐seven eligible RCTs assessing ablative or non‐surgical therapies for patients with small HCC were identified. However, there were limitations and gaps in the RCT evidence and it remains unclear which treatment is most effective in this population. Most of the studies had small sample sizes and reporting of methods was often poor. Most were judged to have a high risk of bias or some risk of bias concerns. For many of the treatment comparisons, data were very limited. The results of the NMA and treatment effectiveness rankings were very uncertain and the threshold analysis showed that results for some comparisons could plausibly change if there were small changes in this data (e.g. MWA vs. RFA for OS). For these comparisons, current results are not robust and further high quality RCTs are needed to draw firm conclusions on effectiveness.

Despite these limitations in the evidence, there is some evidence from the NMA that both RFA and resection are superior to PEI for OS. RFA is also superior to PEI for PFS and recurrence outcomes. RFA was found to be better than PAI for PFS, although based on limited data. The risk of local recurrence was lower for RFA combined with PEI than PEI alone. There was also some evidence to suggest that RFA + Iodine‐125 is more effective than some of the other therapies, and that MWA + Sorafenib is inferior to resection, RFA + Iodine‐125 and RFA + systematic chemotherapy. There were no data from any studies on quality of life outcomes.

No RCTs have been published on some therapies. Some of these treatments (cryoablation, IRE, ECT and SABR) tend to be used in subgroups of patients with tumours that are more challenging to treat with other techniques. The lack of RCTs may therefore be due to difficulties in recruiting patients. There were no RCTs on histotripsy but this is still being evaluated as an investigational product.

Although a small number of similar NMAs have been undertaken,^6^, ^7^, ^8^ they had slightly different inclusion criteria or scope which limits the ability to compare results. All three NMAs included at least some patients with larger HCC (up to 5 cm). These NMAs also focused on a much narrower range of outcomes than our systematic review. Two NMAs assessed OS only^7^, ^8^ and the other NMA assessed recurrence‐free survival and OS, but for the subgroup of patients with HCC ≤3 cm only OS is reported.^6^ Patients and clinical advisors emphasised the importance of assessing recurrence outcomes as well as OS. One previous NMA included a different evidence base of mainly retrospective and prospective cohort studies.^7^

One previous NMA, for which any treatment for early HCC was eligible, ranked RFA + Iodine‐125 as the best treatment.^6^ This NMA included a subgroup analysis of solitary HCC up to 3 cm, with RFA + Iodine‐125 found to improve OS compared to RFA alone. Our results also suggest that RFA + Iodine‐125 may be a promising treatment. However, according to our clinical advisors, this is a treatment that is used in very few centres outside China, which might limit the relevance of this finding to other contexts. Additionally, both our NMA and the NMA by Kamarajah et al.^6^ identified only one RCT^30^ on RFA + Iodine‐125. A Cochrane systematic review of management of early or very early HCC reported that evidence was of low or very low quality.^10^ PEI and PAI were found to be inferior to RFA but no differences in all‐cause mortality were found between other treatments.

Currently RFA and MWA are the preferred ablative treatments in clinical practice.^4^, ^61^ MWA (rather than RFA) is now the standard of care in many centres, partly due to speed and ease of use.^62^ Some treatments, such as cryoablation, HIFU and laser ablation, have not been widely adopted in Western centres. PEI is no longer recommended unless other techniques are not possible.^1^, ^4^

Strengths of this systematic review include the comprehensive searches, robust review methods and the NMAs of four important clinical effectiveness outcomes. The project also involved several patient and clinical advisors throughout, helping to ensure that relevant outcomes were assessed and adding context to the review findings. Limitations include the weak evidence base identified which limited our ability to draw firm conclusions on which treatment is best. It was also not possible to draw any conclusions on quality of life due to a lack of data, despite the importance of this outcome to our patient advisors. The fact that the majority of studies were conducted in Asian countries may limit the generalisability of results to other regions (e.g. Europe), due to differences in aetiology of disease^2^ and treatments available. For example, while RFA is widely used in Asia, MWA has now been more widely adopted in Europe.

## 5 RESEARCH RECOMMENDATIONS

It is difficult to make specific recommendations for feasible future research studies based on the findings of our review. While RCT evidence was completely lacking on some interventions, expert clinical advice suggested that it would likely be difficult to recruit sufficient numbers of patients for trials of some of these therapies as they are generally only appropriate for patients with specific tumour characteristics. However, it may be possible to undertake an international multi‐centre trial for some interventions. For example, our clinical advisors suggested that a trial of this nature of SABR would be of international relevance.

Feasibility studies could explore some of the issues that may arise when undertaking a larger trial. These include the acceptability of interventions to patients, their willingness to take part in a trial, the practicality of delivering interventions and ability to measure relevant outcomes. In terms of outcomes, we recommend that future RCTs assess local recurrence, overall recurrence, OS, PFS, health related quality of life and patient acceptability. Authors should use clear and consistent definitions of these outcomes so that results can easily be compared. It was not possible to undertake subgroup analysis based on specific patient characteristics (e.g. tumour size or number, severity of cirrhosis) due to insufficient data. Research to explore whether these characteristics modify the effect of ablative or non‐surgical therapies would be useful.

## 6 CONCLUSIONS

There is limited or no RCT evidence on many ablative and non‐surgical therapies for early HCC. While there is some evidence from our NMA that PEI and PAI are inferior to RFA and PEI is inferior to resection and RFA + PEI for certain outcomes, it is not possible to draw meaningful overall conclusions on which therapies are the most effective. It is also not known how these treatments might impact on patients' quality of life as no RCTs have assessed this outcome. Future randomised trials on some of the therapies for which evidence was lacking would be useful but may be difficult to undertake.

## AUTHOR CONTRIBUTIONS

**Emily South:** Investigation (equal); writing – original draft (lead); writing – review and editing (lead). **Ros Wade:** Conceptualization (lead); funding acquisition (equal); investigation (equal); writing – review and editing (equal). **Sumayya Anwer:** Formal analysis (lead); investigation (equal); visualization (lead); writing – original draft (supporting); writing – review and editing (equal). **Sahar Sharif‐Hurst:** Conceptualization (equal); formal analysis (equal); investigation (equal); writing – review and editing (equal). **Melissa Harden:** Conceptualization (equal); investigation (equal); writing – review and editing (equal). **Helen Fulbright:** Investigation (equal); writing – review and editing (equal). **Sofia Dias:** Conceptualization (equal); formal analysis (equal); funding acquisition (equal); methodology (equal); writing – review and editing (equal). **Mark Simmonds:** Conceptualization (equal); formal analysis (equal); funding acquisition (equal); methodology (equal); writing – review and editing (equal). **Ian Rowe:** Conceptualization (equal); funding acquisition (equal); investigation (supporting); writing – review and editing (equal). **Patricia Thornton:** Conceptualization (equal); investigation (supporting); writing – review and editing (equal). **Tze Min Wah:** Investigation (supporting); writing – review and editing (equal). **Alison Eastwood:** Conceptualization (lead); funding acquisition (lead); investigation (equal); supervision (lead); writing – review and editing (equal).

## FUNDING INFORMATION

This project was funded by the National Institute for Health and Care Research (NIHR) Health Technology Assessment (HTA) programme (project number NIHR131224) and will be published in full in Health Technology Assessment. This report presents independent research commissioned by the NIHR. The views and opinions expressed by authors in this publication are those of the authors and do not necessarily reflect those of the NHS, the NIHR, MRC, CCF, NETSCC, the HTA programme or the Department of Health and Social Care.

## CONFLICT OF INTEREST STATEMENT

**Ian Rowe:** Honoraria from Roche, outside the submitted work. **Tze Min Wah:** Research grant from Boston Scientific and HistoSonics, research grant and honoraria from Angiodynamics. All other authors report no conflicts of interest.

## REGISTRATION

PROSPERO CRD42020221357.

## Supporting information


Data S1:

## Data Availability

The data that supports the findings of this study are available in the supplementary material of this article.
