# Supplementary material for: The effectiveness of ablative and non‐surgical therapies for early hepatocellular carcinoma: Systematic review and network meta‐analysis of randomised controlled trials
Source: Cancer Med. 2023 Oct 30;12(22):20759–72. doi: 10.1002/cam4.6643 (PMC10709740; doi:10.1002/cam4.6643)
Supplement: Supplementary file 1 — Data S1: [file CAM4-12-20759-s001.docx]

Appendix

# Search strategies

## Search strategies for main therapies (February 2021)

MEDLINE ALL

(includes: Epub Ahead of Print, In-Process & Other Non-Indexed Citations, Ovid MEDLINE Daily and Ovid MEDLINE)

via Ovid <http://ovidsp.ovid.com/>

Date range: 1946 to 1^st^ February, 2021

Date searched: 3^rd^ February 2021

Records retrieved: 2303

The MEDLINE strategy below includes a search filter to limit retrieval to RCTs using the Cochrane Highly Sensitive Search Strategy for identifying randomized trials in MEDLINE: sensitivity-maximizing version (2008 revision); Ovid format.

Lefebvre C, Glanville J, Briscoe S, Littlewood A, Marshall C, Metzendorf M-I, Noel-Storr A, Rader T, Shokraneh F, Thomas J, Wieland LS. Technical Supplement to Chapter 4: Searching for and selecting studies. In: Higgins JPT, Thomas J, Chandler J, Cumpston MS, Li T, Page MJ, Welch VA (eds). *Cochrane Handbook for Systematic Reviews of Interventions* Version 6.2 (updated February 2021). Cochrane, 2021. Available from: [www.training.cochrane.org/handbook](http://www.training.cochrane.org/handbook).

1 Carcinoma, Hepatocellular/ (86979)

2 Liver Neoplasms/ (151355)

3 ((liver or hepatocellular or hepato-cellular or hepatic$) adj3 (carcinoma$ or cancer$ or neoplas$ or tumour$ or tumor$ or malign$)).ti,ab. (150762)

4 (hepatocellularcarcinoma$ or hepatocarcinoma$ or hepato-carcinoma$).ti,ab. (4183)

5 hepatoma$.ti,ab. (28611)

6 HCC.ti,ab. (58929)

7 or/1-6 (234592)

8 Neoplasm Staging/ (177611)

9 (small$ or early or earlystage?).ti,ab. (3163695)

10 (((BCLC or Barcelona-Clinic Liver Cancer) adj3 ("0" or A or A1 or A2 or A3 or A4)) or BCLC0-A).ti,ab. (578)

11 (("1" or "2" or "3" or one or two or three) adj (cm$ or centimet$)).ti,ab. (77157)

12 (1cm$ or 2cm$ or 3cm$).ti,ab. (4783)

13 ((carcinoma$ or tumor$ or tumour$ or lesion$ or nodule$) adj6 (size$ or diameter$)).ti,ab. (124495)

14 (eHCC or sHCC).ti,ab. (251)

15 or/8-13 (3424647)

16 14 or (7 and 15) (50618)

17 Radiofrequency Ablation/ (1071)

18 Catheter Ablation/ (33067)

19 Radiofrequency Therapy/ (1098)

20 ((radiofrequenc$ or radio frequenc$) adj3 ablat$).ti,ab. (20597)

21 RFA.ti,ab. (6924)

22 RF ablation.ti,ab. (2496)

23 RTA.ti,ab. (2494)

24 RFTA.ti,ab. (62)

25 or/17-24 (45285)

26 16 and 25 (3125)

27 Microwaves/ (17445)

28 (microwave$ or micro wave$).ti,ab. (38436)

29 (MWA or MCT or PMCT or PMWA).ti,ab. (7692)

30 or/27-29 (47890)

31 16 and 30 (715)

32 Laser Therapy/ (38202)

33 (laser$ adj2 ablat$).ti,ab. (10008)

34 LTA.ti,ab. (3499)

35 or/32-34 (48883)

36 16 and 35 (142)

37 High-Intensity Focused Ultrasound Ablation/ (1728)

38 Ultrasonic Therapy/ (9714)

39 High intensity focus?ed ultrasound.ti,ab. (3137)

40 HIFU.ti,ab. (2449)

41 or/37-40 (12933)

42 16 and 41 (132)

43 Cryosurgery/ (13169)

44 Cryotherapy/ (5214)

45 (cryoablat$ or cryo-ablat$ or cryotherap$ or cryo-therap$ or cryosurg$ or cryo-surg$).ti,ab. (14455)

46 or/43-45 (23464)

47 16 and 46 (317)

48 Ethanol/ (88324)

49 ((alcohol or ethanol) adj2 (inject$ or ablat$)).ti,ab. (5254)

50 (PEI or PEIT).ti,ab. (8408)

51 or/48-50 (98037)

52 16 and 51 (975)

53 Acetic Acid/ (10269)

54 Acetates/ (39925)

55 (acetic acid adj2 (inject$ or ablat$)).ti,ab. (408)

56 PAI.ti,ab. (14810)

57 PAAI.ti,ab. (19)

58 or/53-57 (62615)

59 16 and 58 (132)

60 Electroporation/ (8033)

61 electroporation.ti,ab. (10705)

62 IRE.ti,ab. (2151)

63 or/60-62 (15275)

64 16 and 63 (122)

65 ((stereotactic or stereotaxic) adj3 ablat$).ti,ab. (1271)

66 ((stereotactic or stereotaxic) adj3 (radiotherap$ or radiation)).ti,ab. (9612)

67 (SABR or SABRT).ti,ab. (803)

68 SBRT.ti,ab. (4238)

69 SABER.ti,ab. (356)

70 or/65-69 (10729)

71 16 and 70 (428)

72 Ablation Techniques/ (2918)

73 (ablat$ adj2 (therap$ or intervention$ or treatment$ or technique$ or method$ or procedure$)).ti,ab. (16748)

74 (ablat$ adj2 (chemical$ or thermal$)).ti,ab. (4065)

75 (ablat$ adj2 (tumour$ or tumor$)).ti,ab. (4083)

76 or/72-75 (24549)

77 16 and 76 (1788)

78 Chemoembolization, Therapeutic/ (5983)

79 (chemo-emboli$ or chemoemboli$).ti,ab. (8271)

80 TACE.ti,ab. (5534)

81 cTACE.ti,ab. (144)

82 (DEBTACE or DEB-TACE).ti,ab. (243)

83 (eluting adj2 bead$).ti,ab. (624)

84 DC bead$.ti,ab. (108)

85 or/78-84 (11130)

86 16 and 85 (3538)

87 Embolization, Therapeutic/ (32829)

88 (embolization$ or embolisation$ or embolize$ or embolise$ or embolizing$ or embolising$ or embolotherap$).ti,ab. (52479)

89 TAE.ti,ab. (2435)

90 or/87-89 (63331)

91 16 and 90 (2015)

92 ((locoregional or loco-regional) adj2 (therap$ or intervention$ or treatment$ or technique$ or method$ or procedure$)).ti,ab. (3346)

93 16 and 92 (531)

94 (Therasphere$ or Thera-sphere$).ti,ab. (79)

95 (SIR-Sphere$ or SIRSphere$).ti,ab. (119)

96 (QuiremSphere$ or Quirem-Sphere$).ti,ab. (4)

97 or/94-96 (167)

98 16 and 97 (44)

99 Microspheres/ (28670)

100 (microsphere$ or sphere$).ti,ab. (76678)

101 (microbead$ or bead$).ti,ab. (56354)

102 or/99-101 (139820)

103 Yttrium Radioisotopes/ (3105)

104 Yttrium/ (3157)

105 Yttrium Isotopes/ (709)

106 (Yttrium$ or 90Yttrium$ or Y90 or Y-90 or 90Y or 90-Y).ti,ab. (9775)

107 Holmium/ (904)

108 (Holmium$ or 166Holmium$ or Ho-166 or Ho166 or 166Ho or 166-Ho).ti,ab. (3496)

109 Radiopharmaceuticals/ (51067)

110 or/103-109 (66136)

111 102 and 110 (1871)

112 ((radioactiv$ or radio-activ$ or radionuclide$ or radio-nuclide$ or radioisotope$ or radio-isotope$ or radiolabel$ or radio-label$ or radiopharmaceutic$ or radio-pharmaceutic$) adj2 (sphere$ or microsphere$ or bead$ or microbead$)).ti,ab. (4168)

113 (radiomicrosphere$ or radio-microsphere$).ti,ab. (33)

114 or/111-113 (5932)

115 16 and 114 (315)

116 Brachytherapy/ (19954)

117 (brachytherap$ or brachy-therap$ or microbrachytherap$).ti,ab. (18064)

118 or/116-117 (25595)

119 118 and (110 or 112 or 113) (1048)

120 16 and 119 (85)

121 (radioemboli$ or radio-emboli$ or radioembolotherap$ or radio-embolotherap$).ti,ab. (1791)

122 TARE.ti,ab. (276)

123 (internal$ adj3 (radiation$ or radiotherap$ or radio therap$ or radionuclide$ or radio-nuclide$ or radioisotope$ or radio-isotope$)).ti,ab. (2446)

124 ((intra-arterial$ or intraarterial$) adj3 (radiation$ or radiotherap$ or radio therap$ or radionuclide$ or radio-nuclide$ or radioisotope$ or radio-isotope$)).ti,ab. (284)

125 ((intra-arterial$ or intraarterial$) adj2 (brachytherap$ or brachy-therap$)).ti,ab. (20)

126 SIRT.ti,ab. (1519)

127 (SIR adj2 (therap$ or treatment$)).ti,ab. (88)

128 (radiation adj2 (segmentectom$ or lobectom$)).ti,ab. (53)

129 or/121-128 (5699)

130 16 and 129 (617)

131 26 or 31 or 36 or 42 or 47 or 52 or 59 or 64 or 71 or 77 (5352)

132 86 or 91 or 93 or 98 or 115 or 120 or 130 (5241)

133 randomized controlled trial.pt. (521951)

134 controlled clinical trial.pt. (94049)

135 randomized.ab. (510387)

136 placebo.ab. (215580)

137 drug therapy.fs. (2274478)

138 randomly.ab. (351559)

139 trial.ab. (541682)

140 groups.ab. (2157357)

141 133 or 134 or 135 or 136 or 137 or 138 or 139 or 140 (4916502)

142 exp animals/ not humans.sh. (4782806)

143 141 not 142 (4274490)

144 131 and 143 (1485)

145 132 and 143 (1633)

146 144 or 145 (2615)

147 limit 146 to yr="2000 -Current" (2303)

**Key:**

/ = subject heading (MeSH heading)

sh = subject heading (MeSH heading)

exp = exploded subject heading (MeSH heading)

$ = truncation

? = optional wild card character - stands for zero or one characters

ti,ab = terms in title or abstract fields

adj3 = terms within three words of each other (any order)

pt = publication type

fs = floating subheading

Embase

via Ovid <http://ovidsp.ovid.com/>

Date range: 1974 to 1^st^ February 2021

Date searched: 3^rd^ February 2021

Records retrieved: 2852

The Embase strategy below includes the Cochrane Embase RCT filter (Ovid format).

Glanville J, Foxlee R, Wisniewski S, Noel-Storr A, Edwards M, Dooley G. [Translating the Cochrane EMBASE RCT filter from the Ovid interface to Embase.com: a case study.](https://www.google.com/url?q=https%3A%2F%2Fwww.ncbi.nlm.nih.gov%2Fpubmed%2F%3Fterm%3DTranslating%2Bthe%2BCochrane%2BEMBASE%2BRCT%2Bfilter%2Bfrom%2Bthe%2BOvid%2Binterface%2Bto%2BEmbase.com&sa=D&sntz=1&usg=AFQjCNH0CofWYrMngcNicVE34LrrrxeXlQ) *Health Info Libr J*. 2019 Jul 22. doi: 10.1111/hir.12269

1 liver cell carcinoma/ (162194)

2 liver cancer/ (33757)

3 ((liver or hepatocellular or hepato-cellular or hepatic$) adj3 (carcinoma$ or cancer$ or neoplas$ or tumour$ or tumor$ or malign$)).ti,ab. (213965)

4 (hepatocellularcarcinoma$ or hepatocarcinoma$ or hepato-carcinoma$).ti,ab. (5720)

5 hepatoma$.ti,ab. (32390)

6 HCC.ti,ab. (95027)

7 or/1-6 (287192)

8 early cancer/ (18733)

9 cancer size/ (68391)

10 cancer staging/ (351293)

11 (small$ or early or earlystage?).ti,ab. (4089821)

12 (((BCLC or Barcelona-Clinic Liver Cancer) adj3 ("0" or A or A1 or A2 or A3 or A4)) or BCLC0-A).ti,ab. (1713)

13 (("1" or "2" or "3" or one or two or three) adj (cm$ or centimet$)).ti,ab. (118443)

14 (1cm$ or 2cm$ or 3cm$).ti,ab. (15664)

15 ((carcinoma$ or tumor$ or tumour$ or lesion$ or nodule$) adj6 (size$ or diameter$)).ti,ab. (199061)

16 (eHCC or sHCC).ti,ab. (381)

17 or/8-15 (4561230)

18 16 or (7 and 17) (76409)

19 radiofrequency ablation/ or radiofrequency catheter ablation/ (35323)

20 ((radiofrequenc$ or radio frequenc$) adj3 ablat$).ti,ab. (33895)

21 RFA.ti,ab. (13495)

22 RF ablation.ti,ab. (4770)

23 RTA.ti,ab. (3238)

24 RFTA.ti,ab. (112)

25 or/19-24 (54268)

26 18 and 25 (6083)

27 microwave thermotherapy/ (2355)

28 (microwave$ or micro wave$).ti,ab. (42308)

29 (MWA or MCT or PMCT or PMWA).ti,ab. (12308)

30 or/27-29 (53536)

31 18 and 30 (1341)

32 laser surgery/ or laser thermotherapy/ (29970)

33 low level laser therapy/ (24761)

34 (laser$ adj2 ablat$).ti,ab. (11732)

35 LTA.ti,ab. (5079)

36 or/32-35 (64097)

37 18 and 36 (232)

38 high intensity focused ultrasound/ (5645)

39 High intensity focus?ed ultrasound.ti,ab. (4434)

40 HIFU.ti,ab. (4015)

41 or/38-40 (6925)

42 18 and 41 (199)

43 cryoablation/ (7661)

44 cryotherapy/ (18674)

45 exp cryosurgery/ (8964)

46 (cryoablat$ or cryo-ablat$ or cryotherap$ or cryo-therap$ or cryosurg$ or cryo-surg$).ti,ab. (20653)

47 or/43-46 (37938)

48 18 and 47 (602)

49 alcohol/ (262986)

50 injection/ or inject$.ti,ab. (1002467)

51 49 and 50 (15476)

52 ((alcohol or ethanol) adj2 (inject$ or ablat$)).ti,ab. (7435)

53 (PEI or PEIT).ti,ab. (10707)

54 or/51-53 (27778)

55 18 and 54 (1359)

56 acetic acid/ (55062)

57 (acetic acid adj2 (inject$ or ablat$)).ti,ab. (571)

58 PAI.ti,ab. (21048)

59 PAAI.ti,ab. (26)

60 or/56-59 (76239)

61 18 and 60 (277)

62 irreversible electroporation/ (935)

63 electroporation.ti,ab. (14271)

64 IRE.ti,ab. (3451)

65 or/62-64 (16754)

66 18 and 65 (244)

67 stereotactic body radiation therapy/ (14557)

68 ((stereotactic or stereotaxic) adj3 ablat$).ti,ab. (2594)

69 ((stereotactic or stereotaxic) adj3 (radiotherap$ or radiation)).ti,ab. (18237)

70 SABR.ti,ab. (2039)

71 (SBRT or SABRT).ti,ab. (10823)

72 SABER.ti,ab. (317)

73 or/67-72 (24024)

74 18 and 73 (1111)

75 ablation therapy/ (19004)

76 tumor ablation/ (4585)

77 (ablat$ adj2 (therap$ or intervention$ or treatment$ or technique$ or method$ or procedure$)).ti,ab. (31253)

78 (ablat$ adj2 (chemical$ or thermal$)).ti,ab. (6405)

79 (ablat$ adj2 (tumour$ or tumor$)).ti,ab. (5915)

80 or/75-79 (50436)

81 18 and 80 (3419)

82 chemoembolization/ (17402)

83 (chemo-emboli$ or chemoemboli$).ti,ab. (14084)

84 (chemoembolotherap$ or chemo-embolotherap$).ti,ab. (6)

85 TACE.ti,ab. (11187)

86 cTACE.ti,ab. (355)

87 (DEBTACE or DEB-TACE).ti,ab. (714)

88 (eluting adj2 bead$).ti,ab,dq. (1453)

89 DC bead$.ti,ab. (316)

90 or/82-89 (23200)

91 18 and 90 (8472)

92 artificial embolization/ (12308)

93 (embolization$ or embolisation$ or embolize$ or embolise$ or embolizing$ or embolising$ or embolotherap$).ti,ab. (77524)

94 arterial embolization/ (4731)

95 TAE.ti,ab. (3692)

96 or/92-95 (82901)

97 18 and 96 (2800)

98 ((locoregional or loco-regional) adj2 (therap$ or intervention$ or treatment$ or technique$ or method$ or procedure$)).ti,ab,dq. (5911)

99 18 and 98 (1344)

100 (Therasphere$ or thera-sphere$).ti,ab,dv. (397)

101 (SIR-Sphere$ or SIRSphere$).ti,ab,dv. (552)

102 (QuiremSphere$ or Quirem-Sphere$).ti,ab,dv. (7)

103 brachytherapy device/ (815)

104 or/100-103 (1462)

105 18 and 104 (227)

106 microsphere/ (32856)

107 (microsphere$ or sphere$).ti,ab. (86590)

108 (microbead$ or bead$).ti,ab. (82280)

109 or/106-108 (172336)

110 yttrium/ (5289)

111 yttrium 90/ (8253)

112 (Yttrium$ or 90Yttrium$ or Y90 or Y-90 or 90Y or 90-Y).ti,ab. (13208)

113 holmium/ (1773)

114 (Holmium$ or 166Holmium$ or Ho-166 or Ho166 or 166Ho or 166-Ho).ti,ab. (5980)

115 radiopharmaceutical agent/ (29356)

116 or/110-115 (53464)

117 109 and 116 (3472)

118 radioactive microsphere/ (981)

119 ((radioactiv$ or radio-activ$ or radionuclide$ or radio-nuclide$ or radioisotope$ or radio-isotope$ or radiolabel$ or radio-label$ or radiopharmaceutic$ or radio-pharmaceutic$) adj2 (sphere$ or microsphere$ or bead$ or microbead$)).ti,ab. (4530)

120 (radiomicrosphere$ or radio-microsphere$).ti,ab. (44)

121 or/117-120 (8164)

122 18 and 121 (825)

123 brachytherapy/ (39112)

124 (brachytherap$ or brachy-therap$ or microbrachytherap$).ti,ab. (31398)

125 or/123-124 (42463)

126 125 and (116 or 118 or 119 or 120) (837)

127 18 and 126 (69)

128 radioembolization/ (2628)

129 selective internal radiation.dq. (306)

130 intra arterial brachytherapy.dq. (1)

131 transarterial radioembolization.dq. (72)

132 (radioemboli$ or radio-emboli$ or radioembolotherap$ or radio-embolotherap$).ti,ab. (3726)

133 TARE.ti,ab. (643)

134 (internal$ adj3 (radiation$ or radiotherap$ or radio-therap$ or radionuclide$ or radio-nuclide$ or radioisotope$ or radio-isotope$)).ti,ab. (3623)

135 ((intra-arterial$ or intraarterial$) adj3 (radiation$ or radiotherap$ or radio-therap$ or radionuclide$ or radio-nuclide$ or radioisotope$ or radio-isotope$)).ti,ab. (384)

136 ((intra-arterial$ or intraarterial$) adj2 (brachytherap$ or brachy-therap$)).ti,ab. (21)

137 SIRT.ti,ab. (2890)

138 (SIR adj2 (therap$ or treatment$)).ti,ab. (214)

139 (radiation adj2 (segmentectom$ or lobectom$)).ti,ab. (121)

140 or/128-139 (10380)

141 18 and 140 (1768)

142 26 or 31 or 37 or 42 or 48 or 55 or 61 or 66 or 74 or 81 (9992)

143 91 or 97 or 99 or 105 or 122 or 127 or 141 (11439)

144 randomized controlled trial/ (643723)

145 controlled clinical trial/ (466422)

146 Random$.ti,ab,ot. (1630417)

147 randomization/ (89947)

148 intermethod comparison/ (268300)

149 placebo.ti,ab,ot. (319556)

150 (compare or compared or comparison).ti,ot. (531000)

151 ((evaluated or evaluate or evaluating or assessed or assess) and (compare or compared or comparing or comparison)).ab. (2249194)

152 (open adj label).ti,ab,ot. (84895)

153 ((double or single or doubly or singly) adj (blind or blinded or blindly)).ti,ab,ot. (241487)

154 double blind procedure/ (181068)

155 parallel group$1.ti,ab,ot. (26971)

156 (crossover or cross over).ti,ab,ot. (109556)

157 ((assign$ or match or matched or allocation) adj5 (alternate or group or groups or intervention or interventions or patient or patients or subject or subjects or participant or participants)).ti,ab,ot. (348130)

158 (assigned or allocated).ti,ab,ot. (409888)

159 (controlled adj7 (study or design or trial)).ti,ab,ot. (370490)

160 (volunteer or volunteers).ti,ab,ot. (254840)

161 human experiment/ (533310)

162 trial.ti,ot. (321229)

163 or/144-162 (5292425)

164 (rat or rats or mouse or mice or swine or porcine or murine or sheep or lambs or pigs or piglets or rabbit or rabbits or cat or cats or dog or dogs or cattle or bovine or monkey or monkeys or trout or marmoset$).ti,ot. and animal experiment/ (1099056)

165 Animal experiment/ not (human experiment/ or human/) (2310394)

166 164 or 165 (2362506)

167 163 not 164 (5114838)

168 142 and 167 (2852)

169 143 and 167 (3457)

170 168 or 169 (5107)

171 limit 170 to yr="2000 -Current" (4927)

172 (conference abstract or "conference review").pt. (4020471)

173 171 not 172 (2852)

**Key:**

/ = subject heading (Emtree heading)

exp = exploded subject heading (Emtree heading)

$ = truncation

? = optional wild card character - stands for zero or one characters

ti,ab = terms in title or abstract fields

dv = terms in the device trade name field

dq = terms in the candidate term word field

adj3 = terms within three words of each other (any order)

adj = terms next to each other (in order specified)

ot = original title field

pt = publication type

Cochrane Central Register of Controlled Trials (CENTRAL)

via Wiley <http://onlinelibrary.wiley.com/>

Date range: Issue 2 of 12, February 2021

Date searched: 3^rd^ February 2021

Records retrieved: 842

#1 MeSH descriptor: [Carcinoma, Hepatocellular] this term only 1761

#2 MeSH descriptor: [Liver Neoplasms] this term only 2373

#3 ((liver or hepatocellular or hepato next cellular or hepatic*) near/3 (carcinoma* or cancer* or neoplas* or tumour* or tumor* or malign*)):ti,ab,kw 8558

#4 (hepatocellularcarcinoma* or hepatocarcinoma* or hepato next carcinoma*):ti,ab,kw 73

#5 hepatoma*:ti,ab,kw 132

#6 HCC:ti,ab,kw 3350

#7 {OR #1-#6} 8984

#8 MeSH descriptor: [Neoplasm Staging] this term only 6426

#9 (small* or early or earlystage*):ti,ab,kw 208191

#10 (((BCLC or Barcelona next Clinic next Liver next Cancer) near/3 ("0" or "A" or A1 or A2 or A3 or A4)) or "BCLC0-A"):ti,ab,kw 125

#11 (("1" or "2" or "3" or one or two or three) next (cm* or centimet*)):ti,ab,kw 10341

#12 (1cm* or 2cm* or 3cm*):ti,ab,kw 1905

#13 ((carcinoma* or tumor* or tumour* or lesion* or nodule*) near/6 (size* or diameter*)):ti,ab,kw 8277

#14 (eHCC or sHCC):ti,ab,kw 16

#15 {OR #8-#13} 226228

#16 #14 or (#7 and #15) 2262

#17 MeSH descriptor: [Radiofrequency Ablation] this term only 45

#18 MeSH descriptor: [Catheter Ablation] this term only 1454

#19 MeSH descriptor: [Radiofrequency Therapy] this term only 97

#20 ((radiofrequenc* or radio next frequenc*) near/3 ablat*):ti,ab,kw 2615

#21 RFA:ti,ab,kw 1079

#22 RF next ablation:ti,ab,kw 308

#23 RTA:ti,ab,kw 98

#24 RFTA:ti,ab,kw 8

#25 {OR #17-#24} 3957

#26 #16 and #25 355

#27 MeSH descriptor: [Microwaves] this term only 214

#28 microwave* or micro next wave*:ti,ab,kw 907

#29 (MWA or MCT or PMCT or PMWA):ti,ab,kw 1240

#30 {OR #27-#29} 2040

#31 #16 AND #30 76

#32 MeSH descriptor: [Laser Therapy] this term only 1993

#33 (laser* near/2 ablat*):ti,ab,kw 912

#34 LTA:ti,ab,kw 218

#35 {OR #32-#34} 2897

#36 #16 and #35 14

#37 MeSH descriptor: [High-Intensity Focused Ultrasound Ablation] this term only 46

#38 MeSH descriptor: [Ultrasonic Therapy] this term only 796

#39 High next intensity next focus?ed next ultrasound:ti,ab,kw 229

#40 HIFU:ti,ab,kw 175

#41 {OR #37-#40} 1032

#42 #16 AND #41 8

#43 MeSH descriptor: [Cryosurgery] this term only 348

#44 MeSH descriptor: [Cryotherapy] this term only 687

#45 (cryoablat* or cryo next ablat* or cryotherap* or cryo next therap* or cryosurg* or cryo next surg*):ti,ab,kw 2873

#46 #43 or #44 or #45 2873

#47 #16 and #46 26

#48 MeSH descriptor: [Ethanol] this term only 3460

#49 ((alcohol or ethanol) near/2 (inject* or ablat*)):ti,ab,kw 366

#50 (PEI or PEIT):ti,ab,kw 304

#51 {OR #48-#50} 3937

#52 #16 and #51 100

#53 MeSH descriptor: [Acetic Acid] this term only 200

#54 MeSH descriptor: [Acetates] this term only 1132

#55 ((acetic next acid) near/2 (inject* or ablat*)):ti,ab,kw 24

#56 PAI:ti,ab,kw 1676

#57 PAAI:ti,ab,kw 8

#58 {OR #53-#57} 2960

#59 #16 and #58 20

#60 MeSH descriptor: [Electroporation] this term only 27

#61 electroporation:ti,ab,kw 201

#62 IRE:ti,ab,kw 152

#63 {OR #60-#62} 313

#64 #16 and #63 4

#65 ((stereotactic or stereotaxic) near/3 ablat*):ti,ab,kw 203

#66 ((stereotactic or stereotaxic) near/3 (radiotherap* or radiation)):ti,ab,kw 1055

#67 (SABR or SABRT):ti,ab,kw 173

#68 SBRT:ti,ab,kw 605

#69 SABER:ti,ab,kw 526

#70 {OR #65-#69} 1679

#71 #16 and #70 54

#72 MeSH descriptor: [Ablation Techniques] this term only 87

#73 (ablat* near/2 (therap* or intervention* or treatment* or technique* or method* or procedure*)):ti,ab,kw 3189

#74 (ablat* near/2 (chemical* or thermal*)):ti,ab,kw 402

#75 (ablat* near/2 (tumour* or tumor*)):ti,ab,kw 203

#76 {OR #72-#75} 3562

#77 #16 and #76 210

#78 MeSH descriptor: [Chemoembolization, Therapeutic] this term only 292

#79 (chemo next emboli* or chemoemboli*):ti,ab,kw 1326

#80 TACE:ti,ab,kw 1129

#81 cTACE:ti,ab,kw 54

#82 (DEBTACE or DEB next TACE):ti,ab,kw 57

#83 (eluting near/2 bead*):ti,ab,kw 104

#84 DC bead*:ti,ab,kw 56

#85 {OR #78-#84} 1619

#86 #16 and #85 471

#87 MeSH descriptor: [Embolization, Therapeutic] this term only 354

#88 (embolization* or embolisation* or embolize* or embolise* or embolizing* or embolising* or embolotherap*):ti,ab,kw 2406

#89 TAE:ti,ab,kw 4102

#90 {OR #87-#89} 6409

#91 #16 and #90 184

#92 ((locoregional or loco next regional) near/2 (therap* or intervention* or treatment* or technique* or method* or procedure*)):ti,ab,kw 558

#93 #16 and #92 43

#94 (Therasphere* or Thera next sphere*):ti,ab,kw 14

#95 (SIR-Sphere* or SIR next Sphere*):ti,ab,kw 47

#96 (QuiremSphere* or Quirem next Sphere*):ti,ab,kw 1

#97 {OR #94-#96} 61

#98 #16 AND #97 9

#99 MeSH descriptor: [Microspheres] this term only 226

#100 (microsphere* or sphere*):ti,ab,kw 1485

#101 (microbead* or bead*):ti,ab,kw 1207

#102 {OR #99-#101} 2639

#103 MeSH descriptor: [Yttrium Radioisotopes] this term only 78

#104 MeSH descriptor: [Yttrium] this term only 119

#105 MeSH descriptor: [Yttrium Isotopes] this term only 8

#106 (Yttrium* or 90Yttrium* or "Y90" or "Y-90" or "90Y" or "90-Y"):ti,ab,kw 1269

#107 MeSH descriptor: [Holmium] this term only 31

#108 (Holmium* or 166Holmium* or "Ho-166" or "Ho166" or "166Ho" or "166-Ho"):ti,ab,kw 545

#109 MeSH descriptor: [Radiopharmaceuticals] this term only 1399

#110 {OR #103-#109} 3146

#111 #102 AND #110 134

#112 ((radioactiv* or (radio next activ*) or radionuclide* or (radio next nuclide*) or radioisotope* or (radio next isotope*) or radiolabel* or (radio next label*) or radiopharmaceutic* or (radio next pharmaceutic*)) near/2 (sphere* or microsphere* or bead* or microbead*)):ti,ab,kw 14

#113 (radiomicrosphere* or (radio next microsphere*)):ti,ab,kw 0

#114 #111 OR #112 OR #113 141

#115 #16 AND #114 24

#116 MeSH descriptor: [Brachytherapy] this term only 686

#117 (brachytherap* or brachy next therap* or microbrachytherap*):ti,ab,kw 2169

#118 {OR #116-#117} 2169

#119 #118 AND (#110 OR #112 OR #113) 35

#120 #16 AND #119 3

#121 (radioemboli* or (radio next emboli*) or radioembolotherap* or (radio next embolotherap*)):ti,ab,kw 114

#122 TARE:ti,ab,kw 162

#123 (internal* near/3 (radiation* or radiotherap* or (radio next therap*) or radionuclide* or (radio next nuclide*) or radioisotope* or (radio next isotope*))):ti,ab,kw 150

#124 ((intraarterial* or (intra next arterial)) near/3 (radiation* or radiotherap* or (radio next therap*) or radionuclide* or (radio next nuclide*) or radioisotope* or (radio next isotope*))):ti,ab,kw 27

#125 ((intraarterial* or (intra next arterial*)) near/2 (brachytherap* or (brachy next therap*))):ti,ab,kw 2

#126 SIRT:ti,ab,kw 140

#127 (SIR near/2 (therap* or treatment*)):ti,ab,kw 18

#128 (radiation near/2 (segmentectom* or lobectom*)):ti,ab,kw 5

#129 {OR #121-#128} 483

#130 #16 AND #129 40

#131 #26 or #31 or #36 or #42 or #47 or #52 or #59 or #64 or #71 or #77 538

#132 #86 OR #91 OR #93 OR #98 OR #115 OR #120 OR #130 581

#133 #131 OR #132 908

#134 #131 or #132 in Trials 884

#135 #131 or #132 with Publication Year from 2000 to 2021, in Trials 842

**Key:**

MeSH descriptor = subject heading (MeSH heading)

* = truncation

ti,ab,kw = terms in title, abstract or keyword fields

near/3 = terms within three words of each other (any order)

next = terms are next to each other

Science Citation Index

via Web of Science, Clarivate Analytics <https://clarivate.com/>

Date range: 1900 – 2^nd^ February 2021

Date searched: 3^rd^ February 2021

Records retrieved: 2867

# 77 2,867 #75 not #76

Timespan=2000-2021

# 76 1,380,326 TI=(animal or animals or rat or rats or mouse or mice or rodent or rodents or porcine or murine or sheep or lamb or lambs or ewe or ewes or pig or pigs or piglet or piglets or sow or sows or minipig or minipigs or rabbit or rabbits or kitten or kittens or dog or dogs or puppy or puppies or monkey or monkeys or horse or horses or foal or foals or equine or calf or calves or cattle or heifer or heifers or hamster or hamsters or chicken or chickens or livestock or alpaca* or llama*)

Timespan=2000-2021

# 75 3,059 #73 OR #72

Timespan=2000-2021

# 74 3,257 #73 OR #72

# 73 1,901 #71 AND #70

# 72 2,229 #71 AND #69

# 71 6,931,396 TS=(random* or control* or trial* or "single blind" or "double blind" or "triple blind" or placebo)

# 70 4,999 #68 OR #61 OR #57 OR #50 OR #48 OR #46 OR #44

# 69 5,936 #38 OR #36 OR #32 OR #30 OR #28 OR #26 OR #24 OR #22 OR #20 OR #18

# 68 653 #67 AND #14

# 67 9,335 #66 OR #65 OR #64 OR #63 OR #62

# 66 77 TS=(radiation NEAR/2 (segmentectom* or lobectom*) )

# 65 2,393 TS=(SIRT or (SIR NEAR/2 (therap* or treatment*) ))

# 64 24 TS=((intra-arterial* or intraarterial*) NEAR/2 (brachytherap* or brachy-therap*) )

# 63 4,602 TS=((internal* or intra-arterial* or intraarterial*) NEAR/3 (radiation* or radiotherap* or radio-therap* or radionuclide* or radio-nuclide* or radioisotope* or radio-isotope*) )

# 62 3,542 TS=(radioemboli* or radio-emboli* or radioembolotherap* or radio-embolotherap* or TARE)

# 61 80 #60 AND #14

# 60 584 #59 AND #58

# 59 52,084 #55 OR #54 OR #52

# 58 27,919 TS=(brachytherap* or brachy-therap*or microbrachytherap*)

# 57 416 #56 AND #14

# 56 5,269 #55 OR #54 OR #53

# 55 21 TS=(radiomicrosphere* or radio-microsphere*)

# 54 2,289 TS=((radioactiv* or radio-activ* or radionuclide* or radio-nuclide* or radioisotope* or radio-isotope* or radiolabel* or radio-label* or radiopharmaceutic* or radio-pharmaceutic*) NEAR/2 (sphere* or microsphere* or bead* or microbead*) )

# 53 3,104 #52 AND #51

# 52 49,922 TS=(Yttrium* or 90Yttrium* or Y90 or Y-90 or 90Y or 90-Y or Holmium* or 166Holmium* or Ho-166 or Ho166 or 166Ho or 166-Ho)

# 51 348,045 TS=(microsphere* or sphere* or microbead* or bead*)

# 50 50 #49 AND #14

# 49 319 TS=(Therasphere* or Thera-sphere* or SIR-Sphere* or SIRSphere* or QuiremSphere* or Quirem-Sphere*)

# 48 472 #47 AND #14

# 47 3,756 TS=((locoregional or loco-regional) NEAR/2 (therap* or intervention* or treatment* or technique* or method* or procedure*) )

# 46 2,006 #45 AND #14

# 45 55,271 TS=(embolization* or embolisation* or embolize* or embolise* or embolizing* or embolising* or embolotherap* or TAE)

# 44 3,676 #43 AND #14

# 43 13,768 #42 OR #41 OR #40 OR #39

# 42 185 TS=(DC NEAR/1 bead*)

# 41 1,200 TS=(eluting NEAR/2 bead*)

# 40 5,966 TS=(TACE or cTACE or DEBTACE or DEB-TACE)

# 39 11,579 TS=(chemo-emboli* or chemoemboli*)

# 38 2,729 #37 AND #14

# 37 31,588 TS=(ablat* NEAR/2 (therap* or intervention* or treatment* or technique* or method* or procedure* or chemical* or thermal* or tumour* or tumor*) )

# 36 447 #35 AND #14

# 35 21,126 #34 OR #33

# 34 10,281 TS=(SABR or SABRT or SBRT or SABER)

# 33 16,227 TS=((stereotactic or stereotaxic) NEAR/3 (ablat* or radiotherap* or radiation) )

# 32 203 #31 AND #14

# 31 22,700 TS=(electroporation or IRE)

# 30 177 #29 AND #14

# 29 17,170 TS=("acetic acid" NEAR/2 (inject* or ablat*) or PAI or PAAI)

# 28 1,645 #27 AND #14

# 27 22,485 TS=((alcohol or ethanol) NEAR/2 (inject* or ablat*) or PEI or PEIT)

# 26 339 #25 AND #14

# 25 15,464 TS=(cryoablat* or cryo-ablat* or cryotherap* or cryo-therap* or cryosurg* or cryo-surg*)

# 24 129 #23 AND #14

# 23 4,161 TS=("High intensity focused ultrasound" or "High intensity focussed ultrasound" or HIFU)

# 22 108 #21 AND #14

# 21 43,123 TS=((laser* NEAR/2 ablat*) or LTA)

# 20 1,009 #19 AND #14

# 19 252,991 TS=(microwave* or (micro NEAR/1 wave*) or MWA or MCT or PMCT or PMWA)

# 18 4,188 #17 AND #14

# 17 41,421 #16 OR #15

# 16 16,326 TS=(RFA or RTA or RFTA or (RF NEAR/1 ablation) )

# 15 32,524 TS=((radiofrequenc* or radio-frequenc*) NEAR/3 ablat*)

# 14 43,788 #13 OR #12

# 13 530 TS=(eHCC or sHCC)

# 12 43,351 #11 AND #5

# 11 5,330,461 #10 OR #9 OR #8 OR #7 OR #6

# 10 103,314 TS=((carcinoma* or tumor* or tumour* or lesion* or nodule*) NEAR/6 (size* or diameter*) )

# 9 5,764 TS=(1cm* or 2cm* or 3cm*)

# 8 635,224 TS=(("1" or "2" or "3" or one or two or three) NEAR/1 (cm or cms or centimet*) )

# 7 767 TS=(((BCLC or "Barcelona Clinic Liver Cancer") NEAR/3 ("0" or "A" or "A1" or "A2" or "A3" or "A4") ) or BCLC0-A)

# 6 4,731,138 TS=(small* or early or earlystage*)

# 5 225,123 #4 OR #3 OR #2 OR #1

# 4 57,158 TS=HCC

# 3 32,070 TS=(hepatoma*)

# 2 4,114 TS=(hepatocellularcarcinoma* or hepatocarcinoma* or hepato-carcinoma*)

# 1 192,908 TS=((liver or hepatocellular or hepato-cellular or hepatic*) NEAR/3 (carcinoma* or cancer* or neoplas* or tumour* or tumor* or malign*) )

**Key:**

TS = topic tag; searches in title, abstract, author keywords and keywords plus fields

TI = search in title field

* = truncation

NEAR/2 = terms within two words of each other (any order)

Cochrane Database of Systematic Reviews (CDSR)

via Wiley <http://onlinelibrary.wiley.com/>

Date range: Issue 2 of 12, February 2021

Date searched: 3^rd^ February 2021

Records retrieved: 43

#1 MeSH descriptor: [Carcinoma, Hepatocellular] this term only 1761

#2 MeSH descriptor: [Liver Neoplasms] this term only 2373

#3 ((liver or hepatocellular or hepato next cellular or hepatic*) near/3 (carcinoma* or cancer* or neoplas* or tumour* or tumor* or malign*)):ti,ab,kw 8558

#4 (hepatocellularcarcinoma* or hepatocarcinoma* or hepato next carcinoma*):ti,ab,kw 73

#5 hepatoma*:ti,ab,kw 132

#6 (HCC or eHCC or sHCC):ti,ab,kw 3364

#7 {OR #1-#6} 8991

#8 MeSH descriptor: [Radiofrequency Ablation] this term only 45

#9 MeSH descriptor: [Catheter Ablation] this term only 1454

#10 MeSH descriptor: [Radiofrequency Therapy] this term only 97

#11 ((radiofrequenc* or radio next frequenc*) near/3 ablat*):ti,ab,kw 2615

#12 RFA:ti,ab,kw 1079

#13 RF next ablation:ti,ab,kw 308

#14 RTA:ti,ab,kw 98

#15 RFTA:ti,ab,kw 8

#16 {OR #8-#15} 3957

#17 #7 and #16 685

#18 MeSH descriptor: [Microwaves] this term only 214

#19 microwave* or micro next wave*:ti,ab,kw 907

#20 (MWA or MCT or PMCT or PMWA):ti,ab,kw 1240

#21 {OR #18-#20} 2040

#22 #7 AND #21 172

#23 MeSH descriptor: [Laser Therapy] this term only 1993

#24 (laser* near/2 ablat*):ti,ab,kw 912

#25 LTA:ti,ab,kw 218

#26 {OR #23-#25} 2897

#27 #7 and #26 23

#28 MeSH descriptor: [High-Intensity Focused Ultrasound Ablation] this term only 46

#29 MeSH descriptor: [Ultrasonic Therapy] this term only 796

#30 High next intensity next focus?ed next ultrasound:ti,ab,kw 229

#31 HIFU:ti,ab,kw 175

#32 {OR #28-#31} 1032

#33 #7 AND #32 19

#34 MeSH descriptor: [Cryosurgery] this term only 348

#35 MeSH descriptor: [Cryotherapy] this term only 687

#36 (cryoablat* or cryo next ablat* or cryotherap* or cryo next therap* or cryosurg* or cryo next surg*):ti,ab,kw 2873

#37 #34 or #35 or #36 2873

#38 #7 and #37 72

#39 MeSH descriptor: [Ethanol] this term only 3460

#40 ((alcohol or ethanol) near/2 (inject* or ablat*)):ti,ab,kw 366

#41 (PEI or PEIT):ti,ab,kw 304

#42 {OR #39-#41} 3937

#43 #7 and #42 204

#44 MeSH descriptor: [Acetic Acid] this term only 200

#45 MeSH descriptor: [Acetates] this term only 1132

#46 ((acetic next acid) near/2 (inject* or ablat*)):ti,ab,kw 24

#47 PAI:ti,ab,kw 1676

#48 PAAI:ti,ab,kw 8

#49 {OR #44-#48} 2960

#50 #7 and #49 28

#51 MeSH descriptor: [Electroporation] this term only 27

#52 electroporation:ti,ab,kw 201

#53 IRE:ti,ab,kw 152

#54 {OR #51-#53} 313

#55 #7 and #54 17

#56 ((stereotactic or stereotaxic) near/3 ablat*):ti,ab,kw 203

#57 ((stereotactic or stereotaxic) near/3 (radiotherap* or radiation)):ti,ab,kw 1055

#58 (SABR or SABRT):ti,ab,kw 173

#59 SBRT:ti,ab,kw 605

#60 SABER:ti,ab,kw 526

#61 {OR #56-#60} 1679

#62 #7 and #61 133

#63 MeSH descriptor: [Ablation Techniques] this term only 87

#64 (ablat* near/2 (therap* or intervention* or treatment* or technique* or method* or procedure*)):ti,ab,kw 3189

#65 (ablat* near/2 (chemical* or thermal*)):ti,ab,kw 402

#66 (ablat* near/2 (tumour* or tumor*)):ti,ab,kw 203

#67 {OR #63-#66} 3562

#68 #7 and #67 395

#69 MeSH descriptor: [Chemoembolization, Therapeutic] this term only 292

#70 (chemo next emboli* or chemoemboli*):ti,ab,kw 1326

#71 TACE:ti,ab,kw 1129

#72 cTACE:ti,ab,kw 54

#73 (DEBTACE or DEB next TACE):ti,ab,kw 57

#74 (eluting near/2 bead*):ti,ab,kw 104

#75 DC bead*:ti,ab,kw 56

#76 {OR #69-#75} 1619

#77 #7 and #76 1455

#78 MeSH descriptor: [Embolization, Therapeutic] this term only 354

#79 (embolization* or embolisation* or embolize* or embolise* or embolizing* or embolising* or embolotherap*):ti,ab,kw 2406

#80 TAE:ti,ab,kw 4102

#81 {OR #78-#80} 6409

#82 #7 and #81 553

#83 ((locoregional or loco next regional) near/2 (therap* or intervention* or treatment* or technique* or method* or procedure*)):ti,ab,kw 558

#84 #7 and #83 144

#85 (Therasphere* or Thera next sphere*):ti,ab,kw 14

#86 (SIR-Sphere* or SIR next Sphere*):ti,ab,kw 47

#87 (QuiremSphere* or Quirem next Sphere*):ti,ab,kw 1

#88 {OR #85-#87} 61

#89 #7 AND #88 47

#90 MeSH descriptor: [Microspheres] this term only 226

#91 (microsphere* or sphere*):ti,ab,kw 1485

#92 (microbead* or bead*):ti,ab,kw 1207

#93 {OR #90-#92} 2639

#94 MeSH descriptor: [Yttrium Radioisotopes] this term only 78

#95 MeSH descriptor: [Yttrium] this term only 119

#96 MeSH descriptor: [Yttrium Isotopes] this term only 8

#97 (Yttrium* or 90Yttrium* or "Y90" or "Y-90" or "90Y" or "90-Y"):ti,ab,kw 1269

#98 MeSH descriptor: [Holmium] this term only 31

#99 (Holmium* or 166Holmium* or "Ho-166" or "Ho166" or "166Ho" or "166-Ho"):ti,ab,kw 545

#100 MeSH descriptor: [Radiopharmaceuticals] this term only 1399

#101 {OR #94-#100} 3146

#102 #93 AND #101 134

#103 ((radioactiv* or (radio next activ*) or radionuclide* or (radio next nuclide*) or radioisotope* or (radio next isotope*) or radiolabel* or (radio next label*) or radiopharmaceutic* or (radio next pharmaceutic*)) near/2 (sphere* or microsphere* or bead* or microbead*)):ti,ab,kw 14

#104 (radiomicrosphere* or (radio next microsphere*)):ti,ab,kw 0

#105 #102 OR #103 OR #104 141

#106 #7 AND #105 104

#107 MeSH descriptor: [Brachytherapy] this term only 686

#108 (brachytherap* or brachy next therap* or microbrachytherap*):ti,ab,kw 2169

#109 {OR #107-#108} 2169

#110 #109 AND (#101 OR #103 OR #104) 35

#111 #7 AND #110 9

#112 (radioemboli* or (radio next emboli*) or radioembolotherap* or (radio next embolotherap*)):ti,ab,kw 114

#113 TARE:ti,ab,kw 162

#114 (internal* near/3 (radiation* or radiotherap* or (radio next therap*) or radionuclide* or (radio next nuclide*) or radioisotope* or (radio next isotope*))):ti,ab,kw 150

#115 ((intraarterial* or (intra next arterial)) near/3 (radiation* or radiotherap* or (radio next therap*) or radionuclide* or (radio next nuclide*) or radioisotope* or (radio next isotope*))):ti,ab,kw 27

#116 ((intraarterial* or (intra next arterial*)) near/2 (brachytherap* or (brachy next therap*))):ti,ab,kw 2

#117 SIRT:ti,ab,kw 140

#118 (SIR near/2 (therap* or treatment*)):ti,ab,kw 18

#119 (radiation near/2 (segmentectom* or lobectom*)):ti,ab,kw 5

#120 {OR #112-#119} 483

#121 #7 AND #120 164

#122 #17 or #22 or #27 or #33 or #38 or #43 or #50 or #55 or #62 or #68 1155

#123 #77 OR #82 OR #84 OR #89 OR #106 OR #111 OR #121 1882

#124 #122 OR #123 in Cochrane Reviews, Cochrane Protocols 43

**Key:**

MeSH descriptor = subject heading (MeSH heading)

* = truncation

ti,ab,kw = terms in title or abstract or keyword fields

near/3 = terms within three words of each other (any order)

next = terms are next to each other

Database of Abstracts of Reviews of Effects (DARE)

via <http://www.crd.york.ac.uk/CRDWeb/>

Date range: Inception – 31^st^ March 2015

Date searched: 3^rd^ February 2021

Records retrieved: 155

1 MeSH DESCRIPTOR Carcinoma, Hepatocellular IN DARE 288

2 MeSH DESCRIPTOR Liver Neoplasms IN DARE 393

3 ((liver or hepatocellular or hepato-cellular or hepatic*) adj3 (carcinoma* or cancer* or neoplas* or tumour* or tumor* or malign*)) IN DARE 506

4 ((carcinoma* or cancer* or neoplas* or tumour* or tumor* or malign*) adj3 (liver or hepatocellular or hepato-cellular or hepatic*) ) IN DARE 384

5 (hepatocellularcarcinoma* or hepatocarcinoma* or hepato-carcinoma* ) IN DARE 0

6 (hepatoma*) IN DARE 2

7 (HCC or eHCC or sHCC) IN DARE 25

8 #1 OR #2 OR #3 OR #4 OR #5 OR #6 OR #7 526

9 MeSH DESCRIPTOR Radiofrequency Ablation EXPLODE ALL TREES IN DARE 179

10 MeSH DESCRIPTOR Catheter Ablation IN DARE 179

11 MeSH DESCRIPTOR Radiofrequency Therapy IN DARE 0

12 ((radiofrequency* or radio-frequenc*) adj3 ablat*) OR (ablat* adj3 (radiofrequency* or radio-frequenc*) ) IN DARE 115

13 (RFA or "RF ablation" or RTA or RFTA) IN DARE 19

14 MeSH DESCRIPTOR Microwaves IN DARE 7

15 (microwave* or micro-wave* or MWA or MCT or PMCT or PMWA) IN DARE 33

16 MeSH DESCRIPTOR Laser Therapy EXPLODE ALL TREES IN DARE 200

17 (laser* adj2 ablat*) OR (ablat* adj2 laser*) OR (LTA) IN DARE 38

18 MeSH DESCRIPTOR High-Intensity Focused Ultrasound Ablation IN DARE 5

19 MeSH DESCRIPTOR Ultrasonic Therapy IN DARE 53

20 ((High intensity focus*) AND (ultrasound) ) IN DARE 15

21 (HIFU) IN DARE 5

22 MeSH DESCRIPTOR Cryosurgery IN DARE 15

23 MeSH DESCRIPTOR Cryotherapy IN DARE 26

24 (cryoablat* or cryo-ablat* or cryotherap* or cryo-therap* or cryosurg* or cryo-surg* ) IN DARE 109

25 MeSH DESCRIPTOR Ethanol IN DARE 28

26 MeSH DESCRIPTOR Acetic Acid IN DARE 8

27 MeSH DESCRIPTOR Acetates IN DARE 26

28 ((alcohol or ethanol or "acetic acid") adj2 (inject* or ablat*)) OR ((inject* or ablat*) adj2 (alcohol or ethanol or "acetic acid")) IN DARE 24

29 (PEI or PEIT) OR (PAI or PAAI) IN DARE 99

30 MeSH DESCRIPTOR Electroporation IN DARE 2

31 (electroporation or IRE ) IN DARE 3

32 ((stereotactic or stereotaxic) adj3 (ablat* or radiotherap* or radiation*) ) OR ((ablat* or radiotherap* or radiation*) adj3 (stereotactic or stereotaxic) ) IN DARE 29

33 MeSH DESCRIPTOR Ablation Techniques IN DARE 20

34 (ablat* adj2 (therap* or intervention* or treatment* or technique* or method* or procedure* or chemical* or thermal* or tumour* or tumor*)) OR ((therap* or intervention* or treatment* or technique* or method* or procedure* or chemical* or thermal* or tumour* or tumor*) adj2 ablat*) IN DARE 215

35 #9 OR #10 OR #11 OR #12 OR #13 OR #14 OR #15 OR #16 OR #17 OR #18 OR #19 OR #20 OR #21 OR #22 OR #23 OR #24 OR #25 OR #26 OR #27 OR #28 OR #29 OR #30 OR #31 OR #32 OR #33 OR #34 791

36 MeSH DESCRIPTOR Chemoembolization, Therapeutic IN DARE 67

37 (chemo-emboli* or chemoemboli* or chemoembolotherap* or chemo-embolotherap* or TACE or cTACE or DEBTACE or DEB-TACE ) IN DARE 85

38 (eluting adj2 bead*) OR (bead* adj2 eluting) IN DARE 5

39 (DC bead*) IN DARE 0

40 MeSH DESCRIPTOR Embolization, Therapeutic IN DARE 65

41 (embolization* or embolisation* or embolize* or embolise* or embolizing* or embolising* or embolotherap* or TAE) IN DARE 126

42 ((locoregional or loco-regional) adj2 (therap* or intervention* or treatment* or technique* or method* or procedure*) ) OR ((therap* or intervention* or treatment* or technique* or method* or procedure*) adj2 (locoregional or loco-regional) ) IN DARE 18

43 #36 OR #37 OR #38 OR #39 OR #40 OR #41 OR #42 209

44 MeSH DESCRIPTOR Microspheres IN DARE 7

45 ((microsphere* or sphere* or micro-sphere* or microbead* or micro-bead* or bead*) ) IN DARE 36

46 ((Therasphere* or Thera-sphere* or SIR-Sphere* or SIRSphere* or QuiremSphere* or Quirem-Sphere*) ) IN DARE 2

47 #44 OR #45 36

48 MeSH DESCRIPTOR Yttrium Radioisotopes IN DARE 6

49 MeSH DESCRIPTOR Yttrium EXPLODE ALL TREES IN DARE 7

50 (Yttrium* or 90Yttrium* or Y90 or Y-90 or 90Y or 90-Y) IN DARE 18

51 MeSH DESCRIPTOR Holmium IN DARE 5

52 (Holmium* or 166Holmium* or Ho-166 or Ho166 or 166Ho or 166-Ho) IN DARE 21

53 MeSH DESCRIPTOR Radiopharmaceuticals IN DARE 184

54 #48 OR #49 OR #50 OR #51 OR #52 OR #53 219

55 #47 AND #54 3

56 ((radioactiv* or radio-activ* or radionuclide* or radio-nuclide* or radioisotope* or radio-isotope* or radiolabel* or radio-label* or radiopharmaceutic* or radio-pharmaceutic*) adj2 (sphere* or microsphere* or bead* or microbead*)) IN DARE 1

57 ((sphere* or microsphere* or bead* or microbead*) adj2 (radioactiv* or radio-activ* or radionuclide* or radio-nuclide* or radioisotope* or radio-isotope* or radiolabel* or radio-label* or radiopharmaceutic* or radio-pharmaceutic*)) IN DARE 1

58 ((radiomicrosphere* or radio-microsphere*)) IN DARE 0

59 #46 OR #55 OR #56 OR #57 OR #58 5

60 MeSH DESCRIPTOR Brachytherapy IN DARE 49

61 ((brachytherap* or brachy-therap* or microbrachytherap*) ) IN DARE 84

62 #60 OR #61 84

63 #54 OR #56 OR #57 OR #58 220

64 #62 AND #63 2

65 (radioemboli* or radio-emboli* or radioembolotherap* or radio-embolotherap*) IN DARE 7

66 (TARE) IN DARE 0

67 (internal* adj3 (radiation* or radiotherap* or radio-therap* or radionuclide* or radio-nuclide* or radioisotope* or radio-isotope*) ) OR ((radiation* or radiotherap* or radio-therap* or radionuclide* or radio-nuclide* or radioisotope* or radio-isotope*) adj3 internal* ) IN DARE 4

68 ((intra-arterial* or intraarterial*) adj3 (radiation* or radiotherap* or radio-therap* or radionuclide* or radio-nuclide* or radioisotope* or radio-isotope*)) OR ((radiation* or radiotherap* or radio-therap* or radionuclide* or radio-nuclide* or radioisotope* or radio-isotope*) adj3 (intra-arterial* or intraarterial*)) IN DARE 2

69 (SIRT) IN DARE 3

70 (SIR adj2 (therap* or treatment*)) OR ((therap* or treatment*) adj2 SIR ) IN DARE 0

71 (radiation adj2 (segmentectom* or lobectom*)) OR ((segmentectom* or lobectom*) adj2 radiation ) IN DARE 0

72 #43 OR #59 OR #64 OR #65 OR #66 OR #67 OR #68 OR #69 OR #70 OR #71 214

73 #8 AND #35 94

74 #8 AND #72 97

75 #73 OR #74 160

76 * IN DARE FROM 2000 TO 2015 43354

77 #75 AND #76 155

**Key:**

MeSH DESCRIPTOR = subject heading (MeSH heading)

* = truncation

adj3 = terms within three words of each other (order specified)

International Health Technology Assessment (HTA) database

via <https://database.inahta.org/>

Date range: Inception – 3^rd^ February 2021

Date searched: 4^th^ February 2021

Records retrieved: 83

1. 49 hits retrieved with date limit 2000-2021

(((ablat* OR radiofrequenc* OR "radio-frequency" OR "radio frequency" OR RFA OR RTA OR RFTA OR microwave* OR "micro-wave" OR "micro wave" OR MWA OR MCT OR PMCT OR PMWA OR laser* OR LTA OR "high intensity focused ultrasound" OR "High intensity focussed ultrasound" OR HIFU OR cryoablat* OR "cryo-ablation" OR "cryo ablation" OR cryotherap* OR "cryo-therapy" OR "cryo therapy" OR cryosurg* OR "cryo-surgery" OR "cryo surgery" OR "alcohol injection" OR "ethanol injection" OR "alcohol injections" OR "ethanol injections" OR PEI OR PEIT OR "acetic acid injection" OR "acetic acid injections" OR PAI OR PAII OR electroporation OR IRE OR stereotactic OR stereotaxic OR SABR OR SABRT OR SBRT OR SABER)[Keywords]) OR ((ablat* OR radiofrequenc* OR "radio-frequency" OR "radio frequency" OR RFA OR RTA OR RFTA OR microwave* OR "micro-wave" OR "micro wave" OR MWA OR MCT OR PMCT OR PMWA OR laser* OR LTA OR "high intensity focused ultrasound" OR "High intensity focussed ultrasound" OR HIFU OR cryoablat* OR "cryo-ablation" OR "cryo ablation" OR cryotherap* OR "cryo-therapy" OR "cryo therapy" OR cryosurg* OR "cryo-surgery" OR "cryo surgery" OR "alcohol injection" OR "ethanol injection" OR "alcohol injections" OR "ethanol injections" OR PEI OR PEIT OR "acetic acid injection" OR "acetic acid injections" OR PAI OR PAII OR electroporation OR IRE OR stereotactic OR stereotaxic OR SABR OR SABRT OR SBRT OR SABER)[abs]) OR ((ablat* OR radiofrequenc* OR "radio-frequency" OR "radio frequency" OR RFA OR RTA OR RFTA OR microwave* OR "micro-wave" OR "micro wave" OR MWA OR MCT OR PMCT OR PMWA OR laser* OR LTA OR "high intensity focused ultrasound" OR "High intensity focussed ultrasound" OR HIFU OR cryoablat* OR "cryo-ablation" OR "cryo ablation" OR cryotherap* OR "cryo-therapy" OR "cryo therapy" OR cryosurg* OR "cryo-surgery" OR "cryo surgery" OR "alcohol injection" OR "ethanol injection" OR "alcohol injections" OR "ethanol injections" OR PEI OR PEIT OR "acetic acid injection" OR "acetic acid injections" OR PAI OR PAII OR electroporation OR IRE OR stereotactic OR stereotaxic OR SABR OR SABRT OR SBRT OR SABER)[Title])) AND (((hepatoma*)[Title] OR (hepatoma*)[abs] OR (hepatoma*)[Keywords] OR (HCC)[Title] OR (HCC)[abs] OR (HCC)[Keywords] OR (eHCC)[Title] OR (eHCC)[abs] OR (eHCC)[keywords] OR (sHCC)[Title] OR (sHCC)[abs] OR (sHCC)[keywords]) OR ((hepatocellularcarcinoma*)[Title] OR (hepatocellularcarcinoma*)[abs] OR (hepatocellularcarcinoma*)[Keywords] OR (hepatocarcinoma*)[Title] OR (hepatocarcinoma*)[abs] OR (hepatocarcinoma*)[Keywords]) OR (((carcinoma*)[Keywords] OR (cancer*)[Keywords] OR (neoplas*)[Keywords] OR (tumour*)[Keywords] OR (tumor*)[Keywords] OR (malign*)[Keywords]) AND ((liver)[Keywords] OR (hepatocellular)[Keywords] OR ("hepato-cellular")[Keywords] OR ("hepato cellular")[Keywords] OR (hepatic*)[Keywords])) OR (((carcinoma*)[abs] OR (cancer*)[abs] OR (neoplas*)[abs] OR (tumour*)[abs] OR (tumor*)[abs] OR (malign*)[abs]) AND ((liver)[abs] OR (hepatocellular)[abs] OR ("hepato-cellular")[abs] OR ("hepato cellular")[abs] OR (hepatic*)[abs])) OR (((carcinoma*)[Title] OR (cancer*)[Title] OR (neoplas*)[Title] OR (tumour*)[Title] OR (tumor*)[Title] OR (malign*)[Title]) AND ((liver)[Title] OR (hepatocellular)[Title] OR ("hepato-cellular")[Title] OR ("hepato cellular")[Title] OR (hepatic*)[Title])) OR ("Liver Neoplasms"[mh]) OR ("Carcinoma, Hepatocellular"[mh]))

2. 34 hits retrieved with date limit 2000-2021

(((chemoemboli* OR "chemo-embolization" OR "chemo-embolisation" OR "chemo embolization" OR "chemo embolisation" OR TACE OR cTACE OR DEBTACE OR "DEB-TACE" OR embolization* OR embolisation* OR embolize* OR embolise* OR embolizing* OR embolising* OR embolotherap* OR TAE OR locoregional OR "loco-regional" OR "loco regional" OR Therasphere* OR "Thera-sphere" OR "Thera-spheres" OR "Thera sphere" OR "Thera spheres" OR "SIR-Sphere" OR "SIR-Spheres" OR SIRSphere* OR "SIR sphere" OR "SIR spheres" OR QuiremSphere* OR "Quirem-Sphere" OR "Quirem-Spheres" OR "Quirem sphere" OR "Quirem spheres" OR microsphere* OR sphere* OR microbead* OR bead* OR radiomicrosphere* OR "radio-microsphere" OR "radio-microspheres" OR "radio microsphere" OR "radio microspheres" OR brachytherap* OR "brachy-therapy" OR "brachy therapy" OR microbrachytherap* OR radioemboli* OR "radio-embolisation" OR "radio-embolization" OR "radio embolization" OR "radio embolization" OR radioembolotherap* OR "radio-embolotherapy" OR "radio-embolotherapies" OR "radio embolotherapy" OR "radio embolotherapies" OR TARE OR SIRT OR "SIR therapy" OR "SIR therapies" OR "SIR treatment" OR "SIR treatments" OR "selective internal radiation" OR segmentectom* OR lobectom*)[Keywords]) OR ((chemoemboli* OR "chemo-embolization" OR "chemo-embolisation" OR "chemo embolization" OR "chemo embolisation" OR TACE OR cTACE OR DEBTACE OR "DEB-TACE" OR embolization* OR embolisation* OR embolize* OR embolise* OR embolizing* OR embolising* OR embolotherap* OR TAE OR locoregional OR "loco-regional" OR "loco regional" OR Therasphere* OR "Thera-sphere" OR "Thera-spheres" OR "Thera sphere" OR "Thera spheres" OR "SIR-Sphere" OR "SIR-Spheres" OR SIRSphere* OR "SIR sphere" OR "SIR spheres" OR QuiremSphere* OR "Quirem-Sphere" OR "Quirem-Spheres" OR "Quirem sphere" OR "Quirem spheres" OR microsphere* OR sphere* OR microbead* OR bead* OR radiomicrosphere* OR "radio-microsphere" OR "radio-microspheres" OR "radio microsphere" OR "radio microspheres" OR brachytherap* OR "brachy-therapy" OR "brachy therapy" OR microbrachytherap* OR radioemboli* OR "radio-embolisation" OR "radio-embolization" OR "radio embolization" OR "radio embolization" OR radioembolotherap* OR "radio-embolotherapy" OR "radio-embolotherapies" OR "radio embolotherapy" OR "radio embolotherapies" OR TARE OR SIRT OR "SIR therapy" OR "SIR therapies" OR "SIR treatment" OR "SIR treatments" OR "selective internal radiation" OR segmentectom* OR lobectom*)[abs]) OR ((chemoemboli* OR "chemo-embolization" OR "chemo-embolisation" OR "chemo embolization" OR "chemo embolisation" OR TACE OR cTACE OR DEBTACE OR "DEB-TACE" OR embolization* OR embolisation* OR embolize* OR embolise* OR embolizing* OR embolising* OR embolotherap* OR TAE OR locoregional OR "loco-regional" OR "loco regional" OR Therasphere* OR "Thera-sphere" OR "Thera-spheres" OR "Thera sphere" OR "Thera spheres" OR "SIR-Sphere" OR "SIR-Spheres" OR SIRSphere* OR "SIR sphere" OR "SIR spheres" OR QuiremSphere* OR "Quirem-Sphere" OR "Quirem-Spheres" OR "Quirem sphere" OR "Quirem spheres" OR microsphere* OR sphere* OR microbead* OR bead* OR radiomicrosphere* OR "radio-microsphere" OR "radio-microspheres" OR "radio microsphere" OR "radio microspheres" OR brachytherap* OR "brachy-therapy" OR "brachy therapy" OR microbrachytherap* OR radioemboli* OR "radio-embolisation" OR "radio-embolization" OR "radio embolization" OR "radio embolization" OR radioembolotherap* OR "radio-embolotherapy" OR "radio-embolotherapies" OR "radio embolotherapy" OR "radio embolotherapies" OR TARE OR SIRT OR "SIR therapy" OR "SIR therapies" OR "SIR treatment" OR "SIR treatments" OR "selective internal radiation" OR segmentectom* OR lobectom*)[Title])) AND (((hepatoma*)[Title] OR (hepatoma*)[abs] OR (hepatoma*)[Keywords] OR (HCC)[Title] OR (HCC)[abs] OR (HCC)[Keywords] OR (eHCC)[Title] OR (eHCC)[abs] OR (eHCC)[keywords] OR (sHCC)[Title] OR (sHCC)[abs] OR (sHCC)[keywords]) OR ((hepatocellularcarcinoma*)[Title] OR (hepatocellularcarcinoma*)[abs] OR (hepatocellularcarcinoma*)[Keywords] OR (hepatocarcinoma*)[Title] OR (hepatocarcinoma*)[abs] OR (hepatocarcinoma*)[Keywords]) OR (((carcinoma*)[Keywords] OR (cancer*)[Keywords] OR (neoplas*)[Keywords] OR (tumour*)[Keywords] OR (tumor*)[Keywords] OR (malign*)[Keywords]) AND ((liver)[Keywords] OR (hepatocellular)[Keywords] OR ("hepato-cellular")[Keywords] OR ("hepato cellular")[Keywords] OR (hepatic*)[Keywords])) OR (((carcinoma*)[abs] OR (cancer*)[abs] OR (neoplas*)[abs] OR (tumour*)[abs] OR (tumor*)[abs] OR (malign*)[abs]) AND ((liver)[abs] OR (hepatocellular)[abs] OR ("hepato-cellular")[abs] OR ("hepato cellular")[abs] OR (hepatic*)[abs])) OR (((carcinoma*)[Title] OR (cancer*)[Title] OR (neoplas*)[Title] OR (tumour*)[Title] OR (tumor*)[Title] OR (malign*)[Title]) AND ((liver)[Title] OR (hepatocellular)[Title] OR ("hepato-cellular")[Title] OR ("hepato cellular")[Title] OR (hepatic*)[Title])) OR ("Liver Neoplasms"[mh]) OR ("Carcinoma, Hepatocellular"[mh]))

**Key:**

[Keywords] = search of keywords field

[abs] = search of abstract field

[Title] = search of title field

[mh] = subject heading search

* = truncation

Epistemonikos

via <https://www.epistemonikos.org/>

Date range: Inception – 4^th^ February 2021

Date searched: 4^th^ February 2021

Records retrieved: 376

(title:((title:(liver OR hepatocellular OR hepato-cellular OR hepatic*) OR abstract:(liver OR hepatocellular OR hepato-cellular OR hepatic*)) AND (title:(hepatocellularcarcinoma* OR hepatocarcinoma* OR hepato-carcinoma* OR hepatoma* OR HCC OR eHCC OR sHCC) OR abstract:(hepatocellularcarcinoma* OR hepatocarcinoma* OR hepato-carcinoma* OR hepatoma* OR HCC OR eHCC OR sHCC))) OR abstract:((title:(liver OR hepatocellular OR hepato-cellular OR hepatic*) OR abstract:(liver OR hepatocellular OR hepato-cellular OR hepatic*)) AND (title:(hepatocellularcarcinoma* OR hepatocarcinoma* OR hepato-carcinoma* OR hepatoma* OR HCC OR eHCC OR sHCC) OR abstract:(hepatocellularcarcinoma* OR hepatocarcinoma* OR hepato-carcinoma* OR hepatoma* OR HCC OR eHCC OR sHCC)))) AND (title:(ablat* OR radiofrequenc* OR radio-frequenc* OR "radio frequency" OR RFA OR RTA OR RFTA OR microwave* OR micro-wave* OR "micro wave" OR MWA OR MCT OR PMCT OR PMWA OR laser* OR LTA OR "High intensity focused ultrasound" OR "High intensity focussed ultrasound" OR HIFU OR cryoablat* OR cryo-ablat* OR cryotherap* OR cryo-therap* OR cryosurg* OR cryo-surg* OR "alcohol injection" OR "ethanol injection" OR "alcohol injections" OR "ethanol injections" OR PEI OR PEIT OR "acetic acid injection" OR "acetic acid injections" OR PAI OR PAII OR electroporation OR IRE OR stereotactic OR stereotaxic OR SABR OR SABRT OR SBRT OR SABER OR chemo-emboli* OR chemoemboli* OR "chemo embolization" OR "chemo embolisation" OR TACE OR cTACE OR DEBTACE OR DEB-TACE OR embolization* OR embolisation* OR embolize* OR embolise* OR embolizing* OR embolising* OR embolotherap* OR TAE OR locoregional OR loco-regional OR "loco regional" OR Therasphere* OR Thera-sphere* OR "Thera sphere" OR "Thera spheres" OR SIR-Sphere* OR SIRSphere* OR "SIR sphere" OR "SIR spheres" OR QuiremSphere* OR Quirem-Sphere* OR "Quirem sphere" OR "Quirem spheres" OR microsphere* OR sphere* OR microbead* OR bead* OR radiomicrosphere* OR radio-microsphere* OR "radio microsphere" OR "radio microspheres" OR brachytherap* OR brachy-therap* OR "brachy therapy" OR microbrachytherap* OR radioemboli* OR radio-emboli* OR radioembolotherap* OR radio-embolotherap* OR TARE OR SIRT OR "SIR therapy" OR "SIR therapies" OR "SIR treatment" OR "SIR treatments" OR "selective internal radiation" OR segmentectom* OR lobectom*) OR abstract:(ablat* OR radiofrequenc* OR radio-frequenc* OR "radio frequency" OR RFA OR RTA OR RFTA OR microwave* OR micro-wave* OR "micro wave" OR MWA OR MCT OR PMCT OR PMWA OR laser* OR LTA OR "High intensity focused ultrasound" OR "High intensity focussed ultrasound" OR HIFU OR cryoablat* OR cryo-ablat* OR cryotherap* OR cryo-therap* OR cryosurg* OR cryo-surg* OR "alcohol injection" OR "ethanol injection" OR "alcohol injections" OR "ethanol injections" OR PEI OR PEIT OR "acetic acid injection" OR "acetic acid injections" OR PAI OR PAII OR electroporation OR IRE OR stereotactic OR stereotaxic OR SABR OR SABRT OR SBRT OR SABER OR chemo-emboli* OR chemoemboli* OR "chemo embolization" OR "chemo embolisation" OR TACE OR cTACE OR DEBTACE OR DEB-TACE OR embolization* OR embolisation* OR embolize* OR embolise* OR embolizing* OR embolising* OR embolotherap* OR TAE OR locoregional OR loco-regional OR "loco regional" OR Therasphere* OR Thera-sphere* OR "Thera sphere" OR "Thera spheres" OR SIR-Sphere* OR SIRSphere* OR "SIR sphere" OR "SIR spheres" OR QuiremSphere* OR Quirem-Sphere* OR "Quirem sphere" OR "Quirem spheres" OR microsphere* OR sphere* OR microbead* OR bead* OR radiomicrosphere* OR radio-microsphere* OR "radio microsphere" OR "radio microspheres" OR brachytherap* OR brachy-therap* OR "brachy therapy" OR microbrachytherap* OR radioemboli* OR radio-emboli* OR radioembolotherap* OR radio-embolotherap* OR TARE OR SIRT OR "SIR therapy" OR "SIR therapies" OR "SIR treatment" OR "SIR treatments" OR "selective internal radiation" OR segmentectom* OR lobectom*))

Filtered by date – 2000-2021

Filtered by publication type – SRs

**Key**:

* = truncation

International Prospective Register of Systematic Reviews (PROSPERO)

via <https://www.crd.york.ac.uk/prospero/>

Date range: Inception – 2^nd^ February 2021

Date searched: 3^rd^ February 2021

Records retrieved: 213

#1 MeSH DESCRIPTOR Carcinoma, Hepatocellular 173

#2 MeSH DESCRIPTOR Liver Neoplasms 248

#3 ((liver or hepatocellular or hepato-cellular or hepatic*) AND (carcinoma* or cancer* or neoplas* or tumour* or tumor* or malign*)):TI,KW 476

#4 (HCC or sHCC or eHCC or hepatocellularcarcinoma* or hepatocarcinoma* or hepato-carcinoma* or hepatoma*):TI,KW 52

#5 #1 OR #2 OR #3 OR #4 512

#6 MeSH DESCRIPTOR Radiofrequency Ablation 4

#7 MeSH DESCRIPTOR Catheter Ablation 91

#8 MeSH DESCRIPTOR Radiofrequency Therapy 2

#9 MeSH DESCRIPTOR Microwaves 10

#10 MeSH DESCRIPTOR Laser therapy 67

#11 MeSH DESCRIPTOR High-Intensity Focused Ultrasound Ablation 3

#12 MeSH DESCRIPTOR Ultrasonic therapy 9

#13 MeSH DESCRIPTOR Cryosurgery 19

#14 MeSH DESCRIPTOR Cryotherapy 20

#15 MeSH DESCRIPTOR Ethanol 112

#16 MeSH DESCRIPTOR acetic acid 6

#17 MeSH DESCRIPTOR Acetates 11

#18 MeSH DESCRIPTOR electroporation 3

#19 MeSH DESCRIPTOR Ablation Techniques 18

#20 ablat* OR radiofrequenc* OR radio-frequenc* OR RFA OR RTA OR RFTA 929

#21 microwave* OR micro-wave* OR MWA OR MCT OR PMCT OR PMWA 158

#22 laser* OR LTA OR "High intensity focused ultrasound" OR "High intensity focussed ultrasound" OR HIFU 1675

#23 cryoablat* OR cryo-ablat* OR cryotherap* OR cryo-therap* OR cryosurg* OR cryo-surg* 300

#24 "alcohol injection" OR "ethanol injection" OR "alcohol injections" OR "ethanol injections" OR PEI OR PEIT 296

#25 "acetic acid injection" OR "acetic acid injections" OR PAI OR PAII 143

#26 electroporation OR IRE OR stereotactic OR stereotaxic OR SABR OR SABRT OR SBRT OR SABER 320

#27 #26 OR #25 OR #24 OR #23 OR #22 OR #21 OR #20 OR #19 OR #18 OR #17 OR #16 OR #15 OR #14 OR #13 OR #12 OR #11 OR #10 OR #9 OR #8 OR #7 OR #6 3340

#28 #5 AND #27 133

#29 MeSH DESCRIPTOR Chemoembolization, Therapeutic 26

#30 MeSH DESCRIPTOR Embolization, Therapeutic 42

#31 MeSH DESCRIPTOR Microspheres 5

#32 MeSH DESCRIPTOR Brachytherapy 22

#33 chemo-emboli* OR chemoemboli* OR TACE OR cTACE OR DEBTACE OR DEB-TACE 130

#34 embolization* OR embolisation* OR embolize* OR embolise* OR embolizing* OR embolising* OR embolotherap* OR TAE OR locoregional OR loco-regional 676

#35 Therasphere* OR Thera-sphere* OR SIR-Sphere* OR SIRSphere* OR QuiremSphere* OR Quirem-Sphere* 3

#36 microsphere* OR sphere* OR microbead* OR bead* OR radiomicrosphere* OR radio-microsphere* 231

#37 brachytherap* OR brachy-therap* OR microbrachytherap* OR radioemboli* OR radio-emboli* OR radioembolotherap* OR radio-embolotherap* 181

#38 TARE OR SIRT OR "SIR therapy" OR "SIR therapies" OR "SIR treatment" OR "SIR treatments" OR "selective internal radiation" OR segmentectom* OR lobectom* 160

#39 #29 OR #30 OR #31 OR #32 OR #33 OR #34 OR #35 OR #36 OR #37 OR #38 1245

#40 #39 AND #5 154

#41 #40 OR #28 213

**Key:**

MeSH DESCRIPTOR = subject heading (MeSH heading)

* = truncation

adj3 = terms within 3 words of each other (order specified)

## Search strategies for wider radiotherapy techniques (March 2021)

MEDLINE ALL

(includes: Epub Ahead of Print, In-Process & Other Non-Indexed Citations, Ovid MEDLINE Daily and Ovid MEDLINE)

via Ovid <http://ovidsp.ovid.com/>

Date range: 1946 to 16^th^ March 2021

Date searched: 17^th^ March 2021

Records retrieved: 399

The MEDLINE strategy below includes a search filter to limit retrieval to RCTs using the Cochrane Highly Sensitive Search Strategy for identifying randomized trials in MEDLINE: sensitivity-maximizing version (2008 revision); Ovid format.

Lefebvre C, Glanville J, Briscoe S, Littlewood A, Marshall C, Metzendorf M-I, Noel-Storr A, Rader T, Shokraneh F, Thomas J, Wieland LS. Technical Supplement to Chapter 4: Searching for and selecting studies. In: Higgins JPT, Thomas J, Chandler J, Cumpston MS, Li T, Page MJ, Welch VA (eds). *Cochrane Handbook for Systematic Reviews of Interventions* Version 6.2 (updated February 2021). Cochrane, 2021. Available from: [www.training.cochrane.org/handbook](http://www.training.cochrane.org/handbook).

1 Carcinoma, Hepatocellular/ (87724)

2 Liver Neoplasms/ (152378)

3 ((liver or hepatocellular or hepato-cellular or hepatic$) adj3 (carcinoma$ or cancer$ or neoplas$ or tumour$ or tumor$ or malign$)).ti,ab. (151424)

4 (hepatocellularcarcinoma$ or hepatocarcinoma$ or hepato-carcinoma$).ti,ab. (4202)

5 hepatoma$.ti,ab. (28603)

6 HCC.ti,ab. (59303)

7 or/1-6 (235478)

8 Neoplasm Staging/ (178539)

9 (small$ or early or earlystage?).ti,ab. (3174425)

10 (((BCLC or Barcelona-Clinic Liver Cancer) adj3 ("0" or A or A1 or A2 or A3 or A4)) or BCLC0-A).ti,ab. (588)

11 (("1" or "2" or "3" or one or two or three) adj (cm$ or centimet$)).ti,ab. (77490)

12 (1cm$ or 2cm$ or 3cm$).ti,ab. (4785)

13 ((carcinoma$ or tumor$ or tumour$ or lesion$ or nodule$) adj6 (size$ or diameter$)).ti,ab. (125055)

14 (eHCC or sHCC).ti,ab. (254)

15 or/8-13 (3436575)

16 14 or (7 and 15) (50862)

17 Electrochemotherapy/ (673)

18 (electrochemotherap* or electro-chemotherap* or electro chemotherap* or electropermeabili?ation).ti,ab. (1115)

19 (electric* adj2 stimulat* adj2 (therap* or chemotherap* or chemo-therap* or chemo therap* or treat*)).ti,ab. (1260)

20 or/17-19 (2670)

21 histotripsy.ti,ab. (209)

22 Radiotherapy/ or Radiotherapy, Conformal/ or Radiotherapy, Intensity-Modulated/ or Radiotherapy, High-Energy/ or Radiotherapy, Image-Guided/ (72234)

23 (radiotherap* or radiation-therap* or radiation therap*).ti,ab. (237815)

24 ((intensity-modulat* or intensity modulat* or volumetric-modulat* or volumetric modulat*) adj4 (arc therap* or arc-therap*)).ti,ab. (2469)

25 (helical* adj4 tomotherap*).ti,ab. (1214)

26 or/22-25 (267755)

27 Proton Therapy/ (3960)

28 (proton* adj4 therap*).ti,ab. (7126)

29 or/27-28 (8349)

30 20 or 21 or 26 or 29 (275176)

31 16 and 30 (1887)

32 randomized controlled trial.pt. (525223)

33 controlled clinical trial.pt. (94097)

34 randomized.ab. (512974)

35 placebo.ab. (216151)

36 drug therapy.fs. (2290533)

37 randomly.ab. (353254)

38 trial.ab. (543763)

39 groups.ab. (2167571)

40 or/32-39 (4942795)

41 31 and 40 (496)

42 exp animals/ not humans.sh. (4800681)

43 41 not 42 (478)

44 limit 43 to yr="2000 -Current" (399)

**Key:**

/ = subject heading (MeSH heading)

sh = subject heading (MeSH heading)

exp = exploded subject heading (MeSH heading)

$ = truncation

? = optional wild card character - stands for zero or one characters

ti,ab = terms in title or abstract fields

adj3 = terms within three words of each other (any order)

pt = publication type

fs = floating subheading

Embase

via Ovid <http://ovidsp.ovid.com/>

Date range: 1974 to 16^th^ March 2021

Date searched: 17^th^ March 2021

Records retrieved: 413

The Embase strategy below includes the Cochrane Embase RCT filter (Ovid format).

Glanville J, Foxlee R, Wisniewski S, Noel-Storr A, Edwards M, Dooley G. [Translating the Cochrane EMBASE RCT filter from the Ovid interface to Embase.com: a case study.](https://www.google.com/url?q=https%3A%2F%2Fwww.ncbi.nlm.nih.gov%2Fpubmed%2F%3Fterm%3DTranslating%2Bthe%2BCochrane%2BEMBASE%2BRCT%2Bfilter%2Bfrom%2Bthe%2BOvid%2Binterface%2Bto%2BEmbase.com&sa=D&sntz=1&usg=AFQjCNH0CofWYrMngcNicVE34LrrrxeXlQ) *Health Info Libr J*. 2019 Jul 22. doi: 10.1111/hir.12269

1 liver cell carcinoma/ (164908)

2 liver cancer/ (34269)

3 ((liver or hepatocellular or hepato-cellular or hepatic$) adj3 (carcinoma$ or cancer$ or neoplas$ or tumour$ or tumor$ or malign$)).ti,ab. (217280)

4 (hepatocellularcarcinoma$ or hepatocarcinoma$ or hepato-carcinoma$).ti,ab. (5825)

5 hepatoma$.ti,ab. (32625)

6 HCC.ti,ab. (96792)

7 or/1-6 (291316)

8 early cancer/ (18991)

9 cancer size/ (69849)

10 cancer staging/ (357155)

11 (small$ or early or earlystage?).ti,ab. (4129554)

12 (((BCLC or Barcelona-Clinic Liver Cancer) adj3 ("0" or A or A1 or A2 or A3 or A4)) or BCLC0-A).ti,ab. (1747)

13 (("1" or "2" or "3" or one or two or three) adj (cm$ or centimet$)).ti,ab. (119627)

14 (1cm$ or 2cm$ or 3cm$).ti,ab. (15837)

15 ((carcinoma$ or tumor$ or tumour$ or lesion$ or nodule$) adj6 (size$ or diameter$)).ti,ab. (201318)

16 (eHCC or sHCC).ti,ab. (390)

17 or/8-15 (4607533)

18 16 or (7 and 17) (77624)

19 electrochemotherapy/ (1342)

20 (electrochemotherap* or electro-chemotherap* or electro chemotherap* or electropermeabili?ation).ti,ab. (1430)

21 (electric* adj2 stimulat* adj2 (therap* or chemotherap* or chemo-therap* or chemo therap* or treat*)).ti,ab. (1792)

22 or/19-21 (3602)

23 histotripsy.ti,ab. (307)

24 radiotherapy/ or conformal radiotherapy/ or intensity modulated radiation therapy/ or megavoltage radiotherapy/ or image guided radiotherapy/ (181651)

25 (radiotherap* or radiation-therap* or radiation therap*).ti,ab. (362654)

26 ((intensity-modulat* or intensity modulat* or volumetric-modulat* or volumetric modulat*) adj4 (arc therap* or arc-therap*)).ti,ab. (4871)

27 (helical* adj4 tomotherap*).ti,ab. (2361)

28 or/24-27 (405023)

29 proton therapy/ (9165)

30 (proton* adj4 therap*).ti,ab. (12175)

31 or/29-30 (14852)

32 22 or 23 or 28 or 31 (416863)

33 18 and 32 (3418)

34 randomized controlled trial/ (652616)

35 controlled clinical trial/ (466860)

36 Random$.ti,ab,ot. (1650636)

37 randomization/ (90688)

38 intermethod comparison/ (270029)

39 placebo.ti,ab,ot. (322542)

40 (compare or compared or comparison).ti,ot. (535093)

41 ((evaluated or evaluate or evaluating or assessed or assess) and (compare or compared or comparing or comparison)).ab. (2280196)

42 (open adj label).ti,ab,ot. (86377)

43 ((double or single or doubly or singly) adj (blind or blinded or blindly)).ti,ab,ot. (243561)

44 double blind procedure/ (182913)

45 parallel group$1.ti,ab,ot. (27255)

46 (crossover or cross over).ti,ab,ot. (110369)

47 ((assign$ or match or matched or allocation) adj5 (alternate or group or groups or intervention or interventions or patient or patients or subject or subjects or participant or participants)).ti,ab,ot. (352016)

48 (assigned or allocated).ti,ab,ot. (414659)

49 (controlled adj7 (study or design or trial)).ti,ab,ot. (375207)

50 (volunteer or volunteers).ti,ab,ot. (256628)

51 human experiment/ (540618)

52 trial.ti,ot. (325654)

53 or/34-52 (5353148)

54 33 and 53 (913)

55 (rat or rats or mouse or mice or swine or porcine or murine or sheep or lambs or pigs or piglets or rabbit or rabbits or cat or cats or dog or dogs or cattle or bovine or monkey or monkeys or trout or marmoset$).ti,ot. and animal experiment/ (1106807)

56 Animal experiment/ not (human experiment/ or human/) (2323653)

57 55 or 56 (2376844)

58 54 not 57 (878)

59 limit 58 to yr="2000 -Current" (854)

60 (conference abstract or "conference review").pt. (4075985)

61 59 not 60 (413)

**Key:**

/ = subject heading (Emtree heading)

exp = exploded subject heading (Emtree heading)

$ = truncation

? = optional wild card character - stands for zero or one characters

ti,ab = terms in title or abstract fields

adj3 = terms within three words of each other (any order)

adj = terms next to each other (in order specified)

ot = original title field

pt = publication type

Cochrane Central Register of Controlled Trials (CENTRAL)

via Wiley <http://onlinelibrary.wiley.com/>

Date searched: 17^th^ March 2021

Records retrieved: 206

#1 MeSH descriptor: [Carcinoma, Hepatocellular] this term only 1776

#2 MeSH descriptor: [Liver Neoplasms] this term only 2383

#3 ((liver or hepatocellular or hepato next cellular or hepatic*) near/3 (carcinoma* or cancer* or neoplas* or tumour* or tumor* or malign*)):ti,ab,kw 8607

#4 (hepatocellularcarcinoma* or hepatocarcinoma* or hepato next carcinoma*):ti,ab,kw 73

#5 hepatoma*:ti,ab,kw 132

#6 HCC:ti,ab,kw 3379

#7 {OR #1-#6} 9034

#8 MeSH descriptor: [Neoplasm Staging] this term only 6470

#9 (small* or early or earlystage*):ti,ab,kw 209536

#10 (((BCLC or Barcelona next Clinic next Liver next Cancer) near/3 ("0" or "A" or A1 or A2 or A3 or A4)) or "BCLC0-A"):ti,ab,kw 126

#11 (("1" or "2" or "3" or one or two or three) next (cm* or centimet*)):ti,ab,kw 10404

#12 (1cm* or 2cm* or 3cm*):ti,ab,kw 1918

#13 ((carcinoma* or tumor* or tumour* or lesion* or nodule*) near/6 (size* or diameter*)):ti,ab,kw 8304

#14 (eHCC or sHCC):ti,ab,kw 16

#15 {OR #8-#13} 227675

#16 #14 or (#7 and #15) 2276

#17 MeSH descriptor: [Electrochemotherapy] this term only 10

#18 (electrochemotherap* or electro next chemotherap* or electropermeabili?ation):ti,ab,kw 50

#19 (electric* near/2 stimulat* near/2 (therap* or chemotherap* or chemo next therap* or treat*)):ti,ab,kw 2662

#20 {OR #17-#19} 2711

#21 histotripsy:ti,ab,kw 1

#22 MeSH descriptor: [Radiotherapy] this term only 1193

#23 MeSH descriptor: [Radiotherapy, Conformal] this term only 355

#24 MeSH descriptor: [Radiotherapy, Intensity-Modulated] this term only 261

#25 MeSH descriptor: [Radiotherapy, High-Energy] this term only 297

#26 MeSH descriptor: [Radiotherapy, Image-Guided] this term only 61

#27 (radiotherap* or radiation next therap*):ti,ab,kw 36588

#28 ((intensity next modulat* or volumetric next modulat*) near/4 (arc next therap*)):ti,ab,kw 188

#29 (helical* near/4 tomotherap*):ti,ab,kw 64

#30 {OR #22-#29} 36609

#31 MeSH descriptor: [Proton Therapy] this term only 44

#32 (proton* near/4 therap*):ti,ab,kw 1352

#33 {OR #31-#32} 1352

#34 #20 or #21 or #30 or #33 40339

#35 #16 and #34 221

with Publication Year from 2000 to 2021, in Trials 206

**Key:**

MeSH descriptor = indexing term (MeSH)

* = truncation

ti,ab,kw = terms in either title or abstract or keyword fields

near/3 = terms within three words of each other (any order)

next = terms are next to each other.

Science Citation Index

via Web of Science, Clarivate Analytics <https://clarivate.com/>

Date range searched: 1900 – 18^th^ March 2021

Date searched: 18^th^ March 2021

Records retrieved: 746

# 30 #28 NOT #29 746

Indexes=SCI-EXPANDED Timespan=2000-2021

# 29 TI=(animal or animals or rat or rats or mouse or mice or rodent or rodents or porcine or murine or sheep or lamb or lambs or ewe or ewes or pig or pigs or piglet or piglets or sow or sows or minipig or minipigs or rabbit or rabbits or kitten or kittens or dog or dogs or puppy or puppies or monkey or monkeys or horse or horses or foal or foals or equine or calf or calves or cattle or heifer or heifers or hamster or hamsters or chicken or chickens or livestock or alpaca* or llama*) 1,391,130

Indexes=SCI-EXPANDED Timespan=2000-2021

# 28 #26 AND #25 773

Indexes=SCI-EXPANDED Timespan=2000-2021

# 27 #26 AND #25 825

Indexes=SCI-EXPANDED Timespan=1900-2021

# 26 TS=(random* or control* or trial* or "single blind" or "double blind" or "triple blind" or placebo) 6,988,447

Indexes=SCI-EXPANDED Timespan=1900-2021

# 25 #24 AND #14 1,713

Indexes=SCI-EXPANDED Timespan=1900-2021

# 24 #23 OR #22 OR #18 292,868

Indexes=SCI-EXPANDED Timespan=1900-2021

# 23 TS=(proton* NEAR/4 therap*) 11,388

Indexes=SCI-EXPANDED Timespan=1900-2021

# 22 #21 OR #20 OR #19 283,146

Indexes=SCI-EXPANDED Timespan=1900-2021

# 21 TS=(helical* NEAR/4 tomotherap*) 2,521

Indexes=SCI-EXPANDED Timespan=1900-2021

# 20 TS=((intensity-modulat* or "intensity modulat*" or volumetric-modulat* or "volumetric modulat*") NEAR/4 ("arc therap*" or arc-therap*) ) 3,054

Indexes=SCI-EXPANDED Timespan=1900-2021

# 19 TS=((radiotherap* or radiation-therap* or "radiation therap*")) 281,566

Indexes=SCI-EXPANDED Timespan=1900-2021

# 18 #17 OR #16 OR #15 3,090

Indexes=SCI-EXPANDED Timespan=1900-2021

# 17 TS=(histotripsy) 323

Indexes=SCI-EXPANDED Timespan=1900-2021

# 16 TS=((electric-stimulation or "electric stimulation") NEAR/2 (therap* or chemotherap* or chemo-therap* or "chemo therap*" or treat*) ) 346

Indexes=SCI-EXPANDED Timespan=1900-2021

# 15 TS=(electrochemotherap* or electro-chemotherap* or "electro chemotherap*" or electropermeabili*) 2,421

Indexes=SCI-EXPANDED Timespan=1900-2021

# 14 #13 OR #12 44,159

Indexes=SCI-EXPANDED Timespan=1900-2021

# 13 TS=(eHCC or sHCC) 540

Indexes=SCI-EXPANDED Timespan=1900-2021

# 12 #11 AND #5 43,714

Indexes=SCI-EXPANDED Timespan=1900-2021

# 11 #10 OR #9 OR #8 OR #7 OR #6 5,372,729

Indexes=SCI-EXPANDED Timespan=1900-2021

# 10 TS=((carcinoma* or tumor* or tumour* or lesion* or nodule*) NEAR/6 (size* or diameter*) ) 104,187

Indexes=SCI-EXPANDED Timespan=1900-2021

# 9 TS=(1cm* or 2cm* or 3cm*) 5,803

Indexes=SCI-EXPANDED Timespan=1900-2021

# 8 TS=(("1" or "2" or "3" or one or two or three) NEAR/1 (cm or cms or centimet*) ) 640,471

Indexes=SCI-EXPANDED Timespan=1900-2021

# 7 TS=(((BCLC or "Barcelona Clinic Liver Cancer") NEAR/3 ("0" or "A" or "A1" or "A2" or "A3" or "A4") ) or BCLC0-A) 778

Indexes=SCI-EXPANDED Timespan=1900-2021

# 6 TS=(small* or early or earlystage$) 4,768,362

Indexes=SCI-EXPANDED Timespan=1900-2021

# 5 #4 OR #3 OR #2 OR #1 227,136

Indexes=SCI-EXPANDED Timespan=1900-2021

# 4 TS=HCC 57,992

Indexes=SCI-EXPANDED Timespan=1900-2021

# 3 TS=(hepatoma*) 32,148

Indexes=SCI-EXPANDED Timespan=1900-2021

# 2 TS=(hepatocellularcarcinoma* or hepatocarcinoma* or hepato-carcinoma*) 4,143

Indexes=SCI-EXPANDED Timespan=1900-2021

# 1 TS=((liver or hepatocellular or hepato-cellular or hepatic*) NEAR/3 (carcinoma* or cancer* or neoplas* or tumour* or tumor* or malign*) ) 194,773

Indexes=SCI-EXPANDED Timespan=1900-2021

**Key:**

TS= terms in either title, abstract, author keywords, and keywords plus fields

TI= search in title field

NEAR/3  = terms within three words of each other (any order).

* = truncation

International HTA database

via <https://database.inahta.org/>

Date range: Inception – 16^th^ March 2021

Date searched: 17^th^ March 2021

Records retrieved: 189

((Carcinoma, Hepatocellular)[mh] OR (Liver Neoplasms)[mh] OR (((liver or hepatocellular or hepato-cellular or hepatic*) and (carcinoma* or cancer* or neoplas* or tumour* or tumor* or malign*)))[Title] OR (((liver or hepatocellular or hepato-cellular or hepatic*) and (carcinoma* or cancer* or neoplas* or tumour* or tumor* or malign*)))[abs] OR (((liver or hepatocellular or hepato-cellular or hepatic*) and (carcinoma* or cancer* or neoplas* or tumour* or tumor* or malign*)))[Keywords] OR ((hepatocellularcarcinoma* or hepatocarcinoma* or hepato-carcinoma*))[Title] OR ((hepatocellularcarcinoma* or hepatocarcinoma* or hepato-carcinoma*))[abs] OR ((hepatocellularcarcinoma* or hepatocarcinoma* or hepato-carcinoma*))[Keywords] OR (hepatoma*)[Title] OR (hepatoma*)[abs] OR (hepatoma*)[Keywords] OR ((HCC or eHCC or sHCC))[Title] OR ((HCC or eHCC or sHCC))[abs] OR ((HCC or eHCC or sHCC))[Keywords] FROM 2000 TO 2021) AND ((Electrochemotherapy)[mh] OR ((electrochemotherap* or electro-chemotherap* or "electro chemotherap*" or electropermeabili*))[Title] OR ((electrochemotherap* or electro-chemotherap* or "electro chemotherap*" or electropermeabili*))[abs] OR ((electrochemotherap* or electro-chemotherap* or "electro chemotherap*" or electropermeabili*))[Keywords] OR (((electric-stimulation or "electric stimulation") and (therap* or chemotherap* or chemo-therap* or chemo therap* or treat*)))[Title] OR (((electric-stimulation or "electric stimulation") and (therap* or chemotherap* or chemo-therap* or chemo therap* or treat*)))[abs] OR (((electric-stimulation or "electric stimulation") and (therap* or chemotherap* or chemo-therap* or chemo therap* or treat*)))[Keywords] OR (histotripsy)[Title] OR (histotripsy)[abs] OR (histotripsy)[Keywords] OR (Radiotherapy)[mh] OR (Radiotherapy, Conformal)[mh] OR (Radiotherapy, Intensity-Modulated)[mh] OR (Radiotherapy, High-Energy)[mh] OR (Radiotherapy, Image-Guided)[mh] OR ((radiotherap* or radiation-therap* or "radiation therap*"))[Title] OR ((radiotherap* or radiation-therap* or "radiation therap*"))[abs] OR ((radiotherap* or radiation-therap* or "radiation therap*"))[Keywords] OR (((intensity-modulat* or "intensity modulat*" or volumetric-modulat* or "volumetric modulat*") and ("arc therap*" or arc-therap*)))[Title] OR (((intensity-modulat* or "intensity modulat*" or volumetric-modulat* or "volumetric modulat*") and ("arc therap*" or arc-therap*)))[abs] OR (((intensity-modulat* or "intensity modulat*" or volumetric-modulat* or "volumetric modulat*") and ("arc therap*" or arc-therap*)))[Keywords] OR (("helical tomotherap*"))[Title] OR (("helical tomotherap*"))[abs] OR (("helical tomotherap*"))[Keywords] OR (Proton Therapy)[mh] OR ("proton therap*")[Title] OR ("proton therap*")[abs] OR ("proton therap*")[Keywords] FROM 2000 TO 2021) with date limit applied: **189** references

**Key:**

[mh] = indexing term: Medical Subject Heading (MeSH)

[mhe] exploded MeSH heading

[Keywords] = search of keywords field

[abs] = search of abstract field

[Title] = search of title field

* = truncation

Cochrane Database of Systematic Reviews (CDSR)

via Wiley <http://onlinelibrary.wiley.com/>

Date searched: 17^th^ March 2021

Records retrieved: 1

#1 MeSH descriptor: [Carcinoma, Hepatocellular] this term only 1776

#2 MeSH descriptor: [Liver Neoplasms] this term only 2383

#3 ((liver or hepatocellular or hepato next cellular or hepatic*) near/3 (carcinoma* or cancer* or neoplas* or tumour* or tumor* or malign*)):ti,ab,kw 8607

#4 (hepatocellularcarcinoma* or hepatocarcinoma* or hepato next carcinoma*):ti,ab,kw 73

#5 hepatoma*:ti,ab,kw 132

#6 HCC:ti,ab,kw 3379

#7 {OR #1-#6} 9034

#8 MeSH descriptor: [Neoplasm Staging] this term only 6470

#9 (small* or early or earlystage*):ti,ab,kw 209536

#10 (((BCLC or Barcelona next Clinic next Liver next Cancer) near/3 ("0" or "A" or A1 or A2 or A3 or A4)) or "BCLC0-A"):ti,ab,kw 126

#11 (("1" or "2" or "3" or one or two or three) next (cm* or centimet*)):ti,ab,kw 10404

#12 (1cm* or 2cm* or 3cm*):ti,ab,kw 1918

#13 ((carcinoma* or tumor* or tumour* or lesion* or nodule*) near/6 (size* or diameter*)):ti,ab,kw 8304

#14 (eHCC or sHCC):ti,ab,kw 16

#15 {OR #8-#13} 227675

#16 #14 or (#7 and #15) 2276

#17 MeSH descriptor: [Electrochemotherapy] this term only 10

#18 (electrochemotherap* or electro next chemotherap* or electropermeabili?ation):ti,ab,kw 50

#19 (electric* near/2 stimulat* near/2 (therap* or chemotherap* or chemo next therap* or treat*)):ti,ab,kw 2662

#20 {OR #17-#19} 2711

#21 histotripsy:ti,ab,kw 1

#22 MeSH descriptor: [Radiotherapy] this term only 1193

#23 MeSH descriptor: [Radiotherapy, Conformal] this term only 355

#24 MeSH descriptor: [Radiotherapy, Intensity-Modulated] this term only 261

#25 MeSH descriptor: [Radiotherapy, High-Energy] this term only 297

#26 MeSH descriptor: [Radiotherapy, Image-Guided] this term only 61

#27 (radiotherap* or radiation next therap*):ti,ab,kw 36588

#28 ((intensity next modulat* or volumetric next modulat*) near/4 (arc next therap*)):ti,ab,kw 188

#29 (helical* near/4 tomotherap*):ti,ab,kw 64

#30 {OR #22-#29} 36609

#31 MeSH descriptor: [Proton Therapy] this term only 44

#32 (proton* near/4 therap*):ti,ab,kw 1352

#33 {OR #31-#32} 1352

#34 #20 or #21 or #30 or #33 40339

#35 #16 and #34 221

with Cochrane Library publication date from Jan 2020 to Mar 2021, in Cochrane Reviews 1

**Key:**

MeSH descriptor = subject heading (MeSH heading)

* = truncation

ti,ab,kw = terms in title, abstract or keyword fields

near/3 = terms within three words of each other (any order)

next = terms are next to each other

Database of Abstracts of Reviews of Effects (DARE)

via <https://www.crd.york.ac.uk/CRDWeb/>

Date range searched: Inception to 31^st^ March 2015.

Date searched: 17^th^ March 2021

Records retrieved: 17

1 MeSH DESCRIPTOR Carcinoma, Hepatocellular IN DARE 288

2 MeSH DESCRIPTOR Liver Neoplasms IN DARE 393

3 ((liver or hepatocellular or hepato-cellular or hepatic*) adj3 (carcinoma* or cancer* or neoplas* or tumour* or tumor* or malign*)) IN DARE 506

4 ((carcinoma* or cancer* or neoplas* or tumour* or tumor* or malign*) adj3 (liver or hepatocellular or hepato-cellular or hepatic*)) IN DARE 384

5 (hepatocellularcarcinoma* or hepatocarcinoma* or hepato-carcinoma*) IN DARE 0

6 (hepatoma*) IN DARE 2

7 (HCC or eHCC or sHCC) IN DARE 25

8 #1 OR #2 OR #3 OR #4 OR #5 OR #6 OR #7 527

9 MeSH DESCRIPTOR Electrochemotherapy IN DARE 4

10 (electrochemotherap* or electro-chemotherap* or electro chemotherap* or electropermeabili*) IN DARE 4

11 (electric* NEAR2 stimulat* NEAR2 (therap* or chemotherap* or chemo-therap* or chemo therap* or treat*)) IN DARE 188

12 #9 OR #10 OR #11 192

13 (histotripsy) IN DARE 0

14 MeSH DESCRIPTOR Radiotherapy IN DARE 100

15 MeSH DESCRIPTOR Radiotherapy, Conformal IN DARE 15

16 MeSH DESCRIPTOR Radiotherapy, Intensity-Modulated IN DARE 17

17 MeSH DESCRIPTOR Radiotherapy, High-Energy IN DARE 2

18 MeSH DESCRIPTOR Radiotherapy, Image-Guided IN DARE 0

19 (radiotherap* or radiation-therap* or radiation therap*) IN DARE 1031

20 ((intensity-modulat* or intensity modulat* or volumetric-modulat* or volumetric modulat*) NEAR4 (arc therap* or arc-therap*)) IN DARE 0

21 (helical* NEAR4 tomotherap*) IN DARE 1

22 #14 OR #15 OR #16 OR #17 OR #18 OR #19 OR #20 OR #21 1031

23 MeSH DESCRIPTOR Proton Therapy IN DARE 2

24 (proton* NEAR4 (therap* or radiotherap* or radiation-therap* or radiation therap*)) IN DARE 109

25 #23 OR #24 109

26 #12 OR #13 OR #22 OR #25 1320

27 #8 AND #26 19

28 (#8 and #26) IN DARE FROM 2000 TO 2021 17

**Key:**

MeSH DESCRIPTOR = indexing term: Medical Subject Heading (MeSH)

EXPLODE ALL TREES = exploded indexing term (MeSH)

* = truncation

NEAR3 = terms within three words of each other (only in the order specified).

Epistemonikos

via <https://www.epistemonikos.org/>

Date searched: 17^th^ March 2021

Records retrieved: 38

(title:((title:((title:(liver OR hepatocellular OR hepato-cellular OR hepatic*) OR abstract:(liver OR hepatocellular OR hepato-cellular OR hepatic*)) AND (title:(hepatocellularcarcinoma* OR hepatocarcinoma* OR hepato-carcinoma* OR hepatoma* OR HCC OR eHCC OR sHCC) OR abstract:(hepatocellularcarcinoma* OR hepatocarcinoma* OR hepato-carcinoma* OR hepatoma* OR HCC OR eHCC OR sHCC))) OR abstract:((title:(liver OR hepatocellular OR hepato-cellular OR hepatic*) OR abstract:(liver OR hepatocellular OR hepato-cellular OR hepatic*)) AND (title:(hepatocellularcarcinoma* OR hepatocarcinoma* OR hepato-carcinoma* OR hepatoma* OR HCC OR eHCC OR sHCC) OR abstract:(hepatocellularcarcinoma* OR hepatocarcinoma* OR hepato-carcinoma* OR hepatoma* OR HCC OR eHCC OR sHCC))))) OR abstract:((title:((title:(liver OR hepatocellular OR hepato-cellular OR hepatic*) OR abstract:(liver OR hepatocellular OR hepato-cellular OR hepatic*)) AND (title:(hepatocellularcarcinoma* OR hepatocarcinoma* OR hepato-carcinoma* OR hepatoma* OR HCC OR eHCC OR sHCC) OR abstract:(hepatocellularcarcinoma* OR hepatocarcinoma* OR hepato-carcinoma* OR hepatoma* OR HCC OR eHCC OR sHCC))) OR abstract:((title:(liver OR hepatocellular OR hepato-cellular OR hepatic*) OR abstract:(liver OR hepatocellular OR hepato-cellular OR hepatic*)) AND (title:(hepatocellularcarcinoma* OR hepatocarcinoma* OR hepato-carcinoma* OR hepatoma* OR HCC OR eHCC OR sHCC) OR abstract:(hepatocellularcarcinoma* OR hepatocarcinoma* OR hepato-carcinoma* OR hepatoma* OR HCC OR eHCC OR sHCC)))))) AND (title:((title:((electrochemotherap* OR electro-chemotherap* OR "electro chemotherap*" OR electropermeabili*)) OR abstract:((electrochemotherap* OR electro-chemotherap* OR "electro chemotherap*" OR electropermeabili*))) OR (title:(((electric-stimulation OR "electric stimulation") AND (therap* OR chemotherap* OR chemo-therap* OR chemo therap* OR treat*))) OR abstract:(((electric-stimulation OR "electric stimulation") AND (therap* OR chemotherap* OR chemo-therap* OR chemo therap* OR treat*)))) OR (title:((histotripsy)) OR abstract:((histotripsy))) OR (title:((radiotherap* OR radiation-therap* OR "radiation therap*")) OR abstract:((radiotherap* OR radiation-therap* OR "radiation therap*"))) OR (title:(((intensity-modulat* OR "intensity modulat*" OR volumetric-modulat* OR "volumetric modulat*") AND ("arc therap*" OR arc-therap*))) OR abstract:(((intensity-modulat* OR "intensity modulat*" OR volumetric-modulat* OR "volumetric modulat*") AND ("arc therap*" OR arc-therap*)))) OR (title:(("helical tomotherap*")) OR abstract:(("helical tomotherap*"))) OR (title:(("proton therap*")) OR abstract:(("proton therap*")))) OR abstract:((title:((electrochemotherap* OR electro-chemotherap* OR "electro chemotherap*" OR electropermeabili*)) OR abstract:((electrochemotherap* OR electro-chemotherap* OR "electro chemotherap*" OR electropermeabili*))) OR (title:(((electric-stimulation OR "electric stimulation") AND (therap* OR chemotherap* OR chemo-therap* OR chemo therap* OR treat*))) OR abstract:(((electric-stimulation OR "electric stimulation") AND (therap* OR chemotherap* OR chemo-therap* OR chemo therap* OR treat*)))) OR (title:((histotripsy)) OR abstract:((histotripsy))) OR (title:((radiotherap* OR radiation-therap* OR "radiation therap*")) OR abstract:((radiotherap* OR radiation-therap* OR "radiation therap*"))) OR (title:(((intensity-modulat* OR "intensity modulat*" OR volumetric-modulat* OR "volumetric modulat*") AND ("arc therap*" OR arc-therap*))) OR abstract:(((intensity-modulat* OR "intensity modulat*" OR volumetric-modulat* OR "volumetric modulat*") AND ("arc therap*" OR arc-therap*)))) OR (title:(("helical tomotherap*")) OR abstract:(("helical tomotherap*"))) OR (title:(("proton therap*")) OR abstract:(("proton therap*")))))

38 results when narrowed to custom range 2000 – 2021, filtered by publication type: systematic reviews

**Key:**

* = truncation

title: = searches in title field

abstract: = searches in abstract field

PROSPERO

via <https://www.crd.york.ac.uk/prospero/>

Date range: Inception – 16^th^ March 2021

Date searched: 17^th^ March 2021

Records retrieved: 27

#1 MeSH DESCRIPTOR Carcinoma, Hepatocellular 178

#2 MeSH DESCRIPTOR Liver Neoplasms 254

#3 ((liver or hepatocellular or hepato-cellular or hepatic*) AND (carcinoma* or cancer* or neoplas* or tumour* or tumor* or malign*)):TI,KW 493

#4 (HCC or sHCC or eHCC or hepatocellularcarcinoma* or hepatocarcinoma* or hepato-carcinoma* or hepatoma*):TI,KW 54

#5 #1 OR #2 OR #3 OR #4 530

#6 MeSH DESCRIPTOR Electrochemotherapy 2

#7 (electrochemotherap* or electro-chemotherap* or electro chemotherap* or electropermeabili*):TI,KW 7

#8 (electric* adj2 stimulat* adj2 (therap* or chemotherap* or chemo-therap* or chemo therap* or treat*)):TI,KW 59

#9 #6 OR #7 OR #8 66

#10 histotripsy:TI,KW 0

#11 MeSH DESCRIPTOR Radiotherapy 68

#12 MeSH DESCRIPTOR Radiotherapy, Conformal 2

#13 MeSH DESCRIPTOR Radiotherapy, Intensity-Modulated 6

#14 MeSH DESCRIPTOR Radiotherapy, High-Energy 0

#15 MeSH DESCRIPTOR Radiotherapy, Image-Guided 4

#16 (radiotherap* or radiation-therap* or radiation therap*):TI,KW 542

#17 ((intensity-modulat* or intensity modulat* or volumetric-modulat* or volumetric modulat*) adj4 (arc therap* or arc-therap*)):TI,KW 1

#18 (helical* adj4 tomotherap*):TI,KW 0

#19 #11 OR #12 OR #13 OR #14 OR #15 OR #16 OR #17 OR #18 566

#20 MeSH DESCRIPTOR Proton Therapy 9

#21 (proton* adj4 (therap* or radiotherap* or radiation-therap* or radiation therap*)):TI,KW 24

#22 #20 OR #21 24

#23 #9 OR #10 OR #19 OR #22 646

#24 #5 AND #23 27

**Key:**

MeSH DESCRIPTOR = indexing term: Medical Subject Heading (MeSH)

EXPLODE ALL TREES = exploded indexing term (MeSH)

* = truncation

adj3 = terms within three words of each other (order specified).

:TI,KW = terms in either title or keyword fields

## Search strategies for clinical trial registries

ClinicalTrials.gov

via <https://clinicaltrials.gov/>

Date searched: 27^th^ April 2021

Three separate searches were used, retrieving 149 records in total, which were imported into EndNote 20 and deduplicated.

Search Strategies:

1. eHCC OR sHCC **= 3 hits**

**Limited to:** Interventional Studies

**First Posted:** 01/01/2010 to 03/04/2023

1. (hepatocellularcarcinoma OR hepatoma OR HCC OR hepatocarcinoma OR “hepatocellular carcinoma” OR “liver cancer” OR “hepatic tumor”) AND (small OR early OR "early stage") **= 104 hits**

**Limited to:** Interventional Studies

**First Posted:** 01/01/2010 to 03/04/2023

1. (hepatocellularcarcinoma OR hepatoma OR HCC OR hepatocarcinoma OR “hepatocellular carcinoma” OR “liver cancer” OR “hepatic tumor”) AND (less OR "equal to" OR diameter OR measure OR "no more than" OR size OR under OR "≤" OR "<" OR cm OR mm) **= 42 hits**

**Limited to:** Interventional Studies

**First Posted:** 01/01/2010 to 03/04/2023

European Union Clinical Trials Register

via [www.clinicaltrialsregister.eu/ctr-search/search](http://www.clinicaltrialsregister.eu/ctr-search/search)

Date searched: 27^th^ April 2021

Two separate searches were used, retrieving 64 records in total, which were imported into EndNote 20 and deduplicated.

Search Strategies:

1. (eHCC OR sHCC) = **0 hits**

**Limited to:** 01/01/2010 - 27/04/2021

1. (hepatocellularcarcinoma OR hepatoma OR HCC OR hepatocarcinoma OR "hepatocellular carcinoma" OR "liver cancer" OR "hepatic tumor") AND (small OR early) = **64 hits**

**Limited to:** 01/01/2010 - 27/04/2021

# Study characteristics, results and risk of bias

## Study characteristics and results

**Table A1:** **Characteristics and results of randomised controlled trials (RCTs) included in the review**

| **Study name and location** | **Participant characteristics** | **Intervention** | **Comparator** | **Main results** |
| --- | --- | --- | --- | --- |
| Abdelaziz, 2014^1^  Egypt | 111 patients (with 128 tumours) ≤5cm; subgroup of 87 tumours ≤3cm | RFA (n=32 tumours ≤3cm) | MWA (n=55 tumours ≤3cm) | **Response:**  RFA: Complete ablation: 30/32 (93.8%) tumours; partial ablation: 2/32 (6.2%) tumours.  MWA: Complete ablation: 54/55 (98.2%) tumours; partial ablation: 1/55 (1.8%) tumours.  **Adverse events:**  There were no major complications or deaths in either group.  No other results reported for ≤3cm tumour subgroup. |
| Aikata, 2006 (conference abstract) ^2^  Authors from Japan | 44 patients with tumours <3cm | RFA+TACE (n=21 patients) | RFA alone (n=23 patients) | **Overall survival:**  RFA+TACE: 1 year: 95.2%; 2 years: 95.2%; 3 years: 84%.  RFA alone: 1 year: 100%; 2 years: 82.6%; 3 years: 73.9%.  **Local tumour progression:**  RFA+TACE: 1 year: 9.5%; 2 years: 9.5%; 3 years: 9.5%.  RFA alone: 1 year: 4.3%; 2 years: 8.7%; 3 years: 8.7%.  **Adverse events:**  There were no major complications in either group. |
| Azab, 2011^3^  Egypt | 90 patients (with 98 tumours) ≤5cm; subgroup of 48 tumours ≤3cm | PEI+RFA (n=16 tumours ≤3cm) | RFA alone (n=16 tumours ≤3cm)  PEI alone (n=16 tumours ≤3cm) | **Response:**  PEI+RFA: 15/16 (93.8%) nodules had complete ablation and 1/16 (6.2%) had partial ablation after one session; 16/16 (100%) nodules had complete ablation after two sessions.  RFA alone: 12/16 (75%) nodules had complete ablation and 4/16 (25%) had partial ablation after one session; 14/16 (87.5%) nodules had complete ablation and 2/16 (12.5%) had partial ablation after two sessions.  PEI alone: 0% nodules had complete ablation after two sessions; 13/16 (81.25%) nodules had complete ablation and 3/16 (18.75%) had partial ablation after all sessions.  **Adverse events:**  There were no mortalities related to any of the techniques.  No other results reported for ≤3cm tumour subgroup. |
| Bian, 2014^4^  China | 127 patients with BCLC stage 0-B; subgroup of 78 patients with tumours <3cm | RFA+Iodine-131 metuximab (n=38 patients with tumours <3cm) | RFA alone (n=40 patients with tumours <3cm) | **Recurrence:**  Comparison between groups:  HR: 0.46 (95% CI: 0.21 to 1.01).  **Adverse events:**  There were no serious adverse events or treatment-related deaths in either group.  No other results reported for <3cm tumour subgroup. |
| Brunello, 2008^5^  Italy | 139 patients with tumours ≤3cm | RFA (n=70 patients) | PEI (n=69 patients) | **Overall survival:**  RFA: Number of events (death): 26.  PEI: Number of events (death): 28.  Comparison between groups:  HR: 0.82 (95% CI: 0.48 to 1.41).  Adjusted HR: 0.88 (95% CI: 0.50 to 1.53).  **Distant intrahepatic recurrence:**  RFA: Number of events: 32.  PEI: Number of events: 35.  **Response:**  RFA: 1-year response: 46/70 (65.7%). Early complete response (30-50 days): 67/70 (95.7%).  PEI: 1-year response: 25/69 (36.2%). Early complete response (30-50 days, patients with 1-year follow-up only): 42/64 (65.6%).  **Adverse events:**  RFA: Treatment emergent AEs: 10 (14.3%) patients; major complications: 2 patients.  PEI: Treatment emergent AEs: 12 (17.4%) patients; major complications: 2 patients.  **Economic outcomes:**  Mean direct medical costs were €4097 for PEI and €6540 for RFA. ICER for using RFA instead of PEI was €8286 (95% CI: 2742 to 20917). |
| Chen, 2005a (reported in Chinese) ^6^  China | 132 patients with tumours ≤5cm; subgroup of 55 patients with tumours ≤3cm | Resection (n=31 patients with tumours ≤3cm) | RFA (n=24 patients with tumours ≤3cm) | No results reported for ≤3cm subgroup, other than Kaplan-Meier curve. |
| Chen, 2005b (reported in Chinese) ^7^  China | 86 patients with tumours ≤5cm; subgroup of 47 patients with tumours ≤3cm | RFA+PEI (n=24 patients with tumours ≤3cm) | RFA alone (n=23 patients with tumours ≤3cm) | **Overall survival:**  RFA+PEI: 1 year: 86.8%; 2 years: 79.0%.  RFA alone: 1 year: 81.2%; 2 years: 64.9%.  **Local recurrence:**  RFA+PEI: 1 year: 4.3%; 2 years: 20.9%.  RFA alone: 1 year: 16.8%; 2 years: 34.1%.  **Adverse events:**  There were no serious adverse events in either group. |
| Chen, 2006^8^  China | 180 patients with tumours ≤5cm; subgroup of 79 patients with tumours ≤3cm | Percutaneous local ablative therapy (initial RFA followed by RFA/PEI if residual tumour, and TACE if residual tumour remained) (n=37 patients with tumours ≤3cm) | Partial hepatectomy (n=42 patients with tumours ≤3cm) | No results reported for ≤3cm subgroup, other than Kaplan-Meier curve.  No significant difference in overall and disease-free survival between the 2 treatment groups in ≤3cm subgroup. |
| Chen, 2014^9^  China | 136 patients with tumours ≤3cm | RFA+Iodine-125 (n=68 patients) | RFA alone (n=68 patients) | **Overall survival:**  RFA+Iodine-125: Mean 95.8 months.  RFA alone: Mean 70.8 months.  RFA+Iodine-125: 1 year: 100%; 2 years: 95.6%; 3 years: 86.7%; 4 years: 73.5%; 5 years: 66.1%.  RFA alone: 1 year: 95.6%; 2 years: 85.2%; 3 years: 75.0%; 4 years: 58.8%; 5 years: 47.0%.  RFA+Iodine-125: Number of events (death): 23.  RFA alone: Number of events (death): 36.  Comparison between groups:  HR: 0.502 (95% CI: 0.313 to 0.806).  **Recurrence:**  RFA+Iodine-125: 1 year: 4.5%; 2 years: 11.8%; 3 years: 22.1%; 4 years: 32.4%; 5 years: 39.8%.  RFA alone: 1 year: 14.8%; 2 years: 25.0%; 3 years: 35.3%; 4 years: 47.1%; 5 years: 57.4%.  RFA+Iodine-125: Number of events: 27.  RFA alone: Number of events: 39.  Comparison between groups:  HR: 0.508 (95% CI: 0.317 to 0.815).  RFA+Iodine-125: Mean time to recurrence: 93 months.  RFA alone: Mean time to recurrence: 66.8 months.  **Response:**  RFA+Iodine-125: Complete ablation after one treatment: 56/68; complete response after two treatments: 12/68.  RFA alone: Complete ablation after one treatment: 49/68; complete response after two treatments: 19/68.  **Adverse events:**  RFA+Iodine-125: AEs grade ≥3: 15 events (not patient numbers)  RFA alone: AEs grade ≥3: 11 events (not patient numbers)  No procedure-related mortalities and no Iodine-125 seed migration from the liver to the heart or other organs. |
| Fang, 2014^10^  China | 120 patients with tumours ≤3cm | RFA (n=60 patients) | Hepatectomy (n=60 patients) | **Overall survival:**  RFA: 1 year: 97.5%; 2 years: 91.2%; 3 years: 82.5%.  Hepatectomy: 1 year: 93.7%; 2 years: 86.2%; 3 years: 77.5%.  **Disease-free survival:**  RFA: 1 year: 91.6%; 2 years: 87.4%; 3 years: 55.4%.  Hepatectomy: 1 year: 90.4%; 2 years: 85.2%; 3 years: 41.3%.  **Recurrence:**  RFA: Number of events: 22.  Hepatectomy: Number of events: 21.  **Response:**  RFA: Complete tumour treatment: 57/60 (95.0%).  Hepatectomy: Complete tumour treatment: 58/60 (96.7%).  **Adverse events:**  RFA: Post-operative complications: 2. Major complications: 1. Hospital mortality: 0. Serious pain requiring analgesic (not included in post-operative complications): 3.  Hepatectomy: Post-operative complications: 17. Major complications: 14. Hospital mortality: 0. Serious pain requiring analgesic (not included in post-operative complications): 43.  **Economic outcomes:**  RFA: ICU stay: 0. Hospital days (mean): 4.3 (SD 1.5).  Hepatectomy: ICU stay: 6 (10%). Hospital days (mean): 11.8 (SD 3.1). |
| Feng, 2012^11^  China | 168 patients with tumours <4cm; subgroup of 56 patients with tumours ≤2cm | RFA (n=31 patients with tumours ≤2cm) | Surgical resection (n=25 patients with tumours ≤2cm) | No results reported for ≤2cm subgroup.  **Adverse events:**  No treatment-related mortality. |
| Ferrari, 2007^12^  Authors from Italy | 81 patients with tumours ≤4cm; subgroup of 28 patients with tumours ≤2.5cm | Laser ablation (n=12 patients with tumours ≤2.5cm) | RFA (n=16 patients with tumours ≤2.5cm) | **Adverse events:**  No deaths or major or minor complications occurred during the procedures in either group and no cases of neoplastic seeding observed.  No other results reported for ≤2.5cm subgroup, other than Kaplan-Meier curve. |
| Gan, 2004 (reported in Chinese) ^13^  China | 38 patients with tumours ≤3cm | RFA alone (n=18 patients) | RFA+  Chemotherapy (n=20 patients) | **Recurrence:**  RFA alone: 1 year: 50% (6/12 analysed).  RFA+Chemotherapy: 1 year: 27% (4/15 analysed).  RFA: Number of events: 6 (1 original location recurrence + 5 other location recurrence).  RFA+Chemotherapy: Number of events: 4 (4 other location recurrence).  **Adverse events:**  There were no serious adverse events in either group. |
| Giorgio, 2011^14^  Italy | 285 patients with tumours ≤3cm | RFA (n=142 patients) | PEI (n=143 patients) | **Overall survival:**  RFA: 1 year: 95%; 2 years: 90%; 3 years: 83%; 4 years: 73%; 5 years: 70%.  PEI: 1 year: 95%; 2 years: 83%; 3 years: 78%; 4 years: 70%; 5 years: 68%.  Comparison between groups:  HR: 0.81 (95% CI: 0.46 to 1.39).  **Local recurrence:**  RFA: 1 year: 4.1%; 2 years: 5.7%; 3 years: 7.8%; 4 years: 8.9%; 5 years: 11.7%.  PEI: 1 year: 5.2%; 2 years: 6.7%; 3 years: 9.4%; 4 years: 11.5%; 5 years: 12.8%.  **Adverse events:**  RFA: Major complication: 0.9%.  PEI: Major complication: 1.9%.  No deaths related to the procedure in either group and no cases of seeding.  **Economic outcomes:**  In Italy the medical costs of both procedures were the same so cost calculated only on basis of technical material. The overall cost for PEI needles plus ethanol was €1359 plus VAT, the cost of the generator plus the RFA electrode needles was €171.000 plus VAT, with a statistically significant difference (p<0.001). |
| Huang, 2005^15^  Taiwan | 82 patients with tumours ≤3cm | PEI (n=40 patients) | Resection (n=42 patients) | **Overall survival:**  PEI: 1 year: 100%; 2 years: 100%; 3 years: 96.7%; 4 years: 92.1%; 5 years: 46.0%.  Resection: 1 year: 97.4%; 2 years: 91.3%; 3 years: 88.1%; 4 years: 88.1%; 5 years: 81.8%.  **Progression-free survival:**  PEI: 1 year: 76.1%; 2 years: 64.5%; 3 years: 49.1%; 4 years: 44.6%; 5 years: 44.6%.  Resection: 1 year: 89.5%; 2 years: 71.3%; 3 years: 60.9%; 4 years: 56.2%; 5 years: 48.2%.  **Recurrence:**  PEI: Number of events: 18.  Resection: Number of events: 15.  **Adverse events:**  PEI: 3 patients had adverse effects (1 decreased blood pressure, 2 wound pain).  Resection: No significant complications. |
| Huang, 2010^16^  China | 230 patients with tumours ≤5cm; subgroup of 159 patients with tumours ≤3cm | RFA (n=88 patients with tumours ≤3cm) | Resection (n=71 patients with tumours ≤3cm) | **Overall survival:**  RFA in patients with solitary tumours ≤3cm (n=57): 1 year: 91.2%; 2 years: 84.2%; 3 years: 77.2%; 4 years: 71.9%; 5 years: 61.4%.  RFA in patients with multifocal tumours ≤3cm (n=31): 1 year: 77.4%; 2 years: 64.5%; 3 years: 58.1%; 4 years: 58.1%; 5 years: 45.2%.  Resection in patients with solitary tumours ≤3cm (n=45): 1 year: 100%; 2 years: 97.8%; 3 years: 95.6%; 4 years: 86.7%; 5 years: 82.2%.  Resection in patients with multifocal tumours ≤3cm (n=26): 1 year: 92.3%; 2 years: 88.5%; 3 years: 80.8%; 4 years: 73.1%; 5 years: 69.2%.  **Adverse events:**  No deaths within same hospital admission.  No other results reported for ≤3cm subgroup, other than separate Kaplan-Meier curves for solitary tumours ≤3cm and multifocal tumours ≤3cm. |
| Huo, 2003^17^  Taiwan | 108 patients with tumours ≤5cm; subgroup of 55 patients with tumours ≤3cm | Sequential TACE and PAI (n=24 patients with tumours ≤3cm) | PAI alone (n=31 patients with tumours ≤3cm) | **Overall survival:**  Sequential TACE and PAI: 1 year: 100%; 3 years: 73%.  PAI alone: 1 year: 100%; 3 years: 49%.  **Cancer-free survival:**  There were no significant differences in cancer-free survival between the two groups of patients with tumour sizes ≤3 cm (p=0.217).  **Adverse events:**  Sequential TACE and PAI: Transient fever, abdominal pain and elevation of liver enzymes were present in the majority of patients (in whole sample) after TACE. No serious complications that necessitated intensive care.  PAI alone: Side-effects relatively mild. Most patients (in whole sample) experienced transient mild to moderate local pain during or after acetic acid injection, which could be controlled with additional analgesics. No serious complications that necessitated intensive care. |
| Izumi, 2019^18^ (conference abstract)  Japan | 308 patients with tumours ≤3cm | RFA (n=148 analysed) | Surgery (n=145 analysed) | **Recurrence-free survival:**  RFA: 3 years: 47.7%.  Surgery: 3 years: 49.8%.  Comparison between groups:  HR: 0.96.  **Adverse events:**  No perioperative mortality in either group. |
| Kim, 2021^19^  South Korea | 144 patients with recurrent/residual tumours <3cm | Proton beam radiotherapy (n=72 patients) | RFA (n=72 patients) | **Overall survival:**  Proton beam radiotherapy: 2 years: 91.7%; 3 years: 80.8%; 4 years: 75.4%.  RFA: 2 years: 90.3%; 3 years: 86.0%; 4 years: 77.0%.  Comparison between groups:  HR (2 years): 1.07 (95% CI: 0.58 to 1.98).  **Progression-free survival:**  Proton beam radiotherapy: Median 13.4 months (90% CI: 7.69 to 16.76).  RFA: Median 13.7 months (90% CI: 9.86 to 18.89).  Proton beam radiotherapy: 2 years: 31.9%; 3 years: 26.3%; 4 years: 18.7%.  RFA: 2 years: 31.9%; 3 years: 17.9%; 4 years: 12.6%.  HR (2 years): 0.99 (95% CI 0.70-1.41)  Proton beam radiotherapy: Number of events (progression): 56  RFA: Number of events (progression): 62  **Adverse events:**  Proton beam radiotherapy: AEs grade ≥3: 0.  RFA: AEs grade ≥3: 9 (16%)  No treatment-related late hepatic failure and death without evidence of disease progression and/or subsequent treatment. |
| Koda, 2001^20^  Japan | 52 patients with tumours <3cm | TACE+PEI (n=26 patients) | PEI alone (n=26 patients) | **Overall survival:**  TACE+PEI: 1 year: 100%; 2 years: 94.4%; 3 years: 80.8%; 5 years: 40.4%.  PEI alone: 1 year: 91.3%; 2 years: 81.6%; 3 years: 65.9%; 5 years: 37.7%.  TACE+PEI: Number of events: 4  PEI alone: Number of events: 8  **Recurrence:**  TACE+PEI local residual disease: 1 year: 3.7%; 2 years: 19.3%; 3 years: 19.3%, 5 years: 19.3%.  TACE+PEI new nodular recurrence: 1 year: 8.7%; 2 years: 41.9%; 3 years: 50.2%; 5 years: 50.2%.  PEI alone local residual disease: 1 year: 34.2%; 2 years: 39.3%; 3 years: 39.3%, 5 years: 39.3%.  PEI alone new nodular recurrence: 1 year: 26.9%; 2 years: 60.1%; 3 years: 80.1%; 5 years: 100%.  TACE+PEI local residual disease: Number of events: 4 nodules (of 31)  PEI alone local residual disease: Number of events: 11 nodules (of 34)  TACE+PEI new nodular recurrence: Number of events: 9  PEI alone new nodular recurrence: Number of events: 17  **Adverse events:**  TACE+PEI: Major complications: 2.  PEI alone: Major complications: 0.  TACE + PEI: After TACE: Continuous abdominal pain: 6; Severe abdominal pain: 6; Fever: 19; High-grade fever: 10; liver dysfunction: 3; leukocytosis: 11; C-reactive protein: 22. After PEI: Continuous abdominal pain: 16; Severe abdominal pain: 7; Fever: 20; High-grade fever: 10; liver dysfunction: 3; leukocytosis: 4; C-reactive protein: 20.  PEI alone: Continuous abdominal pain: 11; Severe abdominal pain: 5; Fever: 22; High-grade fever: 5; liver dysfunction: 3; leukocytosis: 5; C-reactive protein: 15. |
| Lencioni, 2003^21^  Authors from Italy and Germany | 104 patients with tumours ≤5cm (large proportion had tumours ≤3cm) | PEI (n=50 patients with mean tumour size 2.8cm; 84% had tumours ≤3cm) | RFA (n=54 patients with mean tumour size 2.8cm; 88% had tumours ≤3cm) | **Overall survival:**  PEI: 1 year: 96%; 2 years: 88%.  RFA: 1 year: 100%; 2 years: 98%.  PEI: Number of events (death): 5.  RFA: Number of events (death): 1.  **Event-free survival:**  PEI: 1 year: 77%; 2 years: 43%.  RFA: 1 year: 86%; 2 years: 64%.  **Local recurrence:**  PEI: Number of events: 13.  RFA: Number of events: 3.  **Response:**  PEI: 60/73 (82%) tumours had complete response after one cycle; 69/73 (94.5%) tumours had complete response after two cycles.  RFA: 63/69 (91%) tumours had complete response after one session; 68/69 (98.6%) tumours had complete response after two sessions.  **Adverse events:**  PEI: No procedure-related death, haemorrhage, infection, needle-track seeding or hepatic failure. Mild-to-moderate pain requiring analgesics: 13; fever: 5; chemical thrombosis of a portal venous branch: 1.  RFA: No procedure-related death, haemorrhage, infection, needle-track seeding or hepatic failure. Mild-to-moderate pain requiring analgesics: 15; Fever: 10; pleural effusions: 4 lesions; asymptomatic arteriovenous shunts: 3. |
| Lin, 2004^22^  Authors from Taiwan | 157 patients with tumours ≤4cm; subgroup of 114 patients with tumours ≤3cm | RFA (n=37 patients with tumours ≤3cm) | Low dose PEI (n=38 patients with tumours ≤3cm)  High dose PEI (n=39 patients with tumours ≤3cm) | **Overall survival:**  RFA in patients with tumours 1-2cm: 1 year: 96%; 2 years: 84%; 3 years: 78%.  RFA in patients with tumours 2.1-3cm: 1 year: 89%; 2 years: 78%; 3 years: 73%.  Low dose PEI in patients with tumours 1-2cm: 1 year: 94%; 2 years: 78%; 3 years: 70%.  Low dose PEI in patients with tumours 2.1-3cm: 1 year: 84%; 2 years: 70%; 3 years: 62%.  High dose PEI in patients with tumours 1-2cm: 1 year: 93%; 2 years: 80%; 3 years: 72%.  High dose PEI in patients with tumours 2.1-3cm: 1 year: 83%; 2 years: 71%; 3 years: 64%.  **Cancer-free survival:**  RFA in patients with tumours 1-2cm: 1 year: 84%; 2 years: 64%; 3 years: 49%.  RFA in patients with tumours 2.1-3cm: 1 year: 76%; 2 years: 53%; 3 years: 40%.  Low dose PEI in patients with tumours 1-2cm: 1 year: 73%; 2 years: 57%; 3 years: 41%.  Low dose PEI in patients with tumours 2.1-3cm: 1 year: 68%; 2 years: 57%; 3 years: 30%.  High dose PEI in patients with tumours 1-2cm: 1 year: 71%; 2 years: 61%; 3 years: 43%.  High dose PEI in patients with tumours 2.1-3cm: 1 year: 63%; 2 years: 51%; 3 years: 32%.  **Local tumour progression:**  RFA in patients with tumours 1-2cm: 1 year: 4%; 2 years: 9%; 3 years: 9%.  RFA in patients with tumours 2.1-3cm: 1 year: 11%; 2 years: 18%; 3 years: 18%.  Low dose PEI in patients with tumours 1-2cm: 1 year: 8%; 2 years: 13%; 3 years: 13%.  Low dose PEI in patients with tumours 2.1-3cm: 1 year: 18%; 2 years: 37%; 3 years: 37%.  High dose PEI in patients with tumours 1-2cm: 1 year: 7%; 2 years: 12%; 3 years: 12%.  High dose PEI in patients with tumours 2.1-3cm: 1 year: 21%; 2 years: 33%; 3 years: 33%.  **Adverse events:**  RFA: One patient (1.9%) had transient pleural effusion, although not reported whether their tumour was <3cm or not. No other severe adverse effect was observed.  Low dose PEI: No severe adverse effect was observed.  High dose PEI: No severe adverse effect was observed.  **Economic outcomes:**  RFA: Mean hospital stay: 4.4 days (range 3-15).  Low dose PEI: Mean hospital stay: 1.6 days (range 2-3).  High dose PEI: Mean hospital stay: 2.1 days (range 2-4). |
| Lin, 2005^23^  Taiwan | 187 patients with tumours ≤3cm | RFA (n=62 patients) | PEI (n=62 patients)  PAI (n=63 patients) | **Overall survival:**  RFA: 1 year: 93%; 2 years: 81%; 3 years: 74%.  PEI: 1 year: 88%; 2 years: 66%: 3 years: 51%.  PAI: 1 year: 90%; 2 years: 67%; 3 years: 53%.  RFA: Number of events (death): 10 (16.1%).  PEI: Number of events (death): 17 (27.4%).  PAI: Number of events (death): 15 (23.8%).  **Cancer-free survival:**  RFA: 1 year: 74%; 2 years: 60%; 3 years: 43%.  PEI: 1 year: 70%; 2 years: 41%: 3 years: 21%.  PAI: 1 year: 71%; 2 years: 43%; 3 years: 23%.  **Recurrence:**  RFA: 1 year: 10%; 2 years: 14%; 3 years: 14%.  PEI: 1 year: 16%; 2 years: 34%: 3 years: 34%.  PAI: 1 year: 14%; 2 years: 31%; 3 years: 31%.  RFA: Number of local recurrence events: 8/60 (13.3%).  PEI: Number of local recurrence events: 19/55 (34.5%).  PAI: Number of local recurrence events: 17/58 (29.3%).  **Complete response:**  RFA: 96.1% (75/78 tumours).  PEI: 88.1% (67/76 tumours).  PAI: 92.4% (73/79 tumours).  **Adverse events:**  RFA: 4.8% (3/62 patients) had serious adverse events (2 patients with haemothorax and 1 with gastric bleeding and perforation).  PEI: No serious adverse events.  PAI: No serious adverse events.  **Economic outcomes:**  RFA: Mean hospitalisation: 4.2 days (range 3-18).  PEI: Mean hospitalisation: 1.7 days (range 2-3).  PAI: Mean hospitalisation: 2.2 days (range 2-5). |
| Liu, 2016^24^  China | 200 patients with tumours ≤5cm; subgroup of 135 patients with tumours ≤3cm | Partial hepatectomy (n=66 patients with tumours ≤3cm) | TACE+RFA (n=69 patients with tumours ≤3cm) | No results reported for ≤3cm subgroup, other than Kaplan-Meier curve.  **Adverse events:**  No 30- or 90-day mortality in either group. |
| Mizuki, 2010^25^  Japan | 30 patients with tumours ≤4cm (large proportion had tumours ≤3cm) | PEI alone (n=14 patients with average tumour size 2.64cm) | TACE+PEI (n=16 patients with average tumour size 2.65cm) | **Overall survival:**  PEI alone: Mean 57.2 months (95% CI: 37.2 to 77.2).  TACE+PEI: Mean 42.4 months (95% CI: 29.2 to 55.6).  PEI alone: Number of events (death): 6/14 (44%).  TACE+PEI: Number of events (death): 8/13 (61.5%)  **Cancer-free survival:**  PEI alone: Mean 16.7 months (95% CI: 7.3 to 26.0).  TACE+PEI: Mean 22.9 months (95% CI: 12.4 to 33.4).  **Recurrence:**  PEI alone: Number of events: 10 (71.4%).  TACE+PEI: Number of events: 11 (84.6%).  **Adverse events:**  In all 30 cases, serious adverse effects or complications were not related to treatment with TACE and/or PEI. |
| Ng, 2017^26^  China | 218 patients with tumours ≤5cm; subgroup of 55 patients with tumours ≤2cm | Resection (n=29 patients with tumours ≤2cm) | RFA (n=26 patients with tumours ≤2cm) | **Overall survival:**  Resection: 1 year: 100%; 3 years: 93%; 5 years: 76%.  RFA: 1 year: 100%; 3 years: 89%: 5 years: 69%.  **Disease-free survival:**  Resection: 1 year: 83%; 3 years: 66%; 5 years: 52%.  RFA: 1 year: 77%; 3 years: 62%: 5 years: 46%.  **Economic outcomes:**  Resection: Median hospital stay: 7 days.  RFA: Median hospital stay: 4 days. |
| Orlacchio, 2014^27^  Italy | 30 patients with tumours ≤4cm (mean tumour size 2.4cm) | Laser ablation (n=15 patients with mean tumour size 2.34cm) | RFA (n=15 patients with mean tumour size 2.41cm) | **Overall survival:**  Laser ablation: Number of events (death): 0  RFA: Number of events (death): 0  **Progression-free survival:**  Laser ablation: 3 months: 85%; 6 months: 62%; 1 year: 54%.  RFA: 3 months: 92%; 6 months: 86%; 1 year: 86%.  **Recurrence:**  Laser ablation: Number of events (local disease progression): 6 (40%).  RFA: Number of events (local disease progression): 2 (13.3%).  **Complete response:**  Laser ablation: 66.7% nodules (10/15) after first procedure; 3/5 patients after a second procedure (87% in total).  RFA: 86.7% nodules (13/15) after first procedure; 1/2 patients after a second procedure (93% in total).  **Adverse events:**  Laser ablation: 2 (13.3%) (1 vasovagal reaction and 1 postablation syndrome).  RFA: 14 (93.3%) (2 vasovagal reactions and 12 postablation syndrome).  No major complications in either arm. |
| Peng, 2012^28^  China | 139 patients with recurrent HCC tumours ≤5cm; subgroup of 87 patients with tumours ≤3cm. | RFA+TACE (n=41 patients with tumours ≤3cm) | RFA alone (n=46 patients with tumours ≤3cm) | **Overall survival:**  RFA+TACE: 1 year: 98%; 3 years: 70%; 5 years: 50%.  RFA alone: 1 year: 83%; 3 years: 60%: 5 years: 50%.  **Recurrence-free survival:**  RFA+TACE: 1 year: 90%; 3 years: 48%; 5 years: 48%.  RFA alone: 1 year: 86%; 3 years: 26%: 5 years: 26%. |
| Shibata, 2002^29^  Japan | 72 patients (with 94 tumours) <4cm; subgroup of 88 tumours ≤3cm | RFA (n=36 patients, 3 of which had tumours 3-4cm) | MWA (n=36 patients, 3 of which had tumours 3-4cm) | **Response:**  RFA: 46/48 (96%) nodules showed complete response and 2 (4%) had residual lesions or incomplete response.  MWA: 41/46 (89%) nodules showed complete response and 5 (11%) showed incomplete response.  **Adverse events:**  RFA: 1 major complication (3%) (segmental hepatic infarction).  MWA: 4 major complications (11%) (liver abscess, cholangitis, subcutaneous abscess with skin burn and subcapsular hematoma).  No other results reported, other than Kaplan-Meier curve. |
| Shibata, 2009^30^  Japan | 89 patients with tumours ≤3cm | RFA+TACE (n=46 patients) | RFA alone (n=43 patients) | **Overall survival:**  RFA+TACE: 1 year: 100%; 2 years: 100%; 3 years: 84.8%; 4 years: 72.7%.  RFA alone: 1 year: 100%; 2 years: 88.8%: 3 years: 84.5%; 4 years: 74%.  RFA+TACE: Number of events (death): 5 (10.9%).  RFA alone: Number of events (death): 6 (13.9%).  **Event-free survival:**  RFA+TACE: 1 year: 71.3%; 2 years: 59.9%; 3 years: 48.8%; 4 years: 36.6%.  RFA alone: 1 year: 74.3%; 2 years: 52.4%; 3 years: 29.7%; 4 years: 29.7%.  **Local progression-free survival** RFA+TACE: 1 year: 84.6%; 2 years: 81.1%; 3 years: 69.7%; 4 years: 55.8%.  RFA alone: 1 year: 88.4%; 2 years: 74.1%; 3 years: 74.1%; 4 years: 61.7%.  **Local tumour progression:**  RFA+TACE: 1 year: 14.4%; 2 years: 17.6%; 3 years: 17.6%; 4 years: 17.6%.  RFA alone: 1 year: 11.4%; 2 years: 14.4%; 3 years: 14.4%; 4 years: 14.4%.  RFA+TACE: Number of recurrence events: 8 (17.4%).  RFA alone: Number of recurrence events: 6 (13.9%).  **Complete response:**  RFA+TACE: 100%.  RFA alone: 100%.  **Adverse events:**  RFA+TACE: 1 major complication (2%) (segmental hepatic infarction).  RFA alone: 1 major complication (2%) (pseudoaneurysm of the anterosuperior branch of the right hepatic artery). |
| Shiina, 2005^31^  Japan | 232 patients with tumours ≤3cm | RFA (n=118 patients) | PEI (n=114 patients) | **Overall survival:**  RFA: 4 years: 74% (95% CI: 65% to 84%).  PEI: 4 years: 57% (95% CI: 45% to 71%).  RFA: Number of events (death): 25 (21.2%).  PEI: Number of events (death): 40 (35.1%).  Comparison between groups: RR: 0.54 (95% CI: 0.33 to 0.89).  **Recurrence:**  RFA: Number of events: 78 (66.1%) (new lesion: 74; local tumour progression: 2; extrahepatic recurrence: 2).  PEI: Number of events: 90 (78.9%) (new lesion: 73; local tumour progression: 13; extrahepatic recurrence: 4).  Comparison between groups: RR: 0.57 (95% CI: 0.41 to 0.80).  Local tumour progression: RR: 0.12 (95% CI 0.03 to 0.55)  **Adverse events:**  RFA: AEs grade ≥3: 6 (5.1%) (1 transient jaundice, 1 skin burn, 1 hepatic infarction and 3 seeding of malignant cells).  PEI: AEs grade ≥3: 3 (2.6%) (1 liver abscess and 2 neoplastic seeding).  **Economic outcomes:**  RFA: Length of hospitalisation: 10.8 ± 5.5 days.  PEI: Length of hospitalisation: 26.1 ± 9.9 days. |
| Vietti Violi, 2018^32^  Switzerland and France | 152 patients with tumours ≤4cm (mean tumour size 1.8cm, <8% patients had tumours >3cm) | MWA (n=76 patients, 6 of which had tumours 3-4cm) | RFA (n=76 patients, 5 of which had tumours 3-4cm) | **Overall survival:**  MWA: 2 years: 86%.  RFA: 2 years: 84%.  **Recurrence:**  MWA: 2 years: 6% (local tumour progression).  RFA: 2 years: 12% (local tumour progression).  Comparison between groups: RR: 1.62 (95% CI: 0.66 to 3.94).  **Time to progression:**  MWA: Median 12 months (95% CI: 5 to 28).  RFA: Median 16 months (95% CI: 4 to 24).  Comparison between groups: HR: 0.72 (95% CI: 0.44 to 1.18).  **Response:**  MWA: 95% achieved complete response after 1 treatment; 5% achieved complete response after 2 treatments.  RFA: 96% achieved complete response after 1 treatment; 4% achieved complete response after 2 treatments.  **Adverse events:**  MWA: 2 (2%) grade 4 adverse events; 5 (5%) grade 1-2 adverse events.  RFA: 3 (3%) grade 3 adverse events; 12 (11.5%) grade 1-2 adverse events.  No treatment related deaths in either group. |
| Xia, 2020^33^  China | 240 patients with recurrent HCC tumours ≤5cm; subgroup of 159 patients with tumours ≤3cm | RFA (78 patients with tumours ≤3cm) | Repeat hepatectomy (81 patients with tumours ≤3cm) | **Overall survival:**  Comparison between group: In HR: 0.05 (95% CI: -0.4 to 0.5).  HR: 1.05 (95% CI: 0.67 to 1.65) (Calculated by CRD)  **Repeat-recurrence-free survival:**  Comparison between group: In HR: 0.07 (95% CI: -0.34 to 0.47).  HR: 1.07 (95% CI: 0.71 to 1.6) (Calculated by CRD) |
| Yan, 2016^34^  China | 120 patients with tumours ≤3cm | Resection (n=60 patients) | MWA+  Sorafenib (n=60 patients) | **Overall survival:**  Resection: Mean 51.2 ± 1.5 months.  MWA+Sorafenib: Mean 64.6 ± 2.4 months.  Resection: 1 year: 90.7%; 3 years: 71.5%; 5 years: 56.7%.  MWA+Sorafenib: 1 year: 91.1%; 3 years: 72.8%; 5 years: 57.5%.  **Tumour-free survival:**  Resection: 1 year: 87.8%; 3 years: 44.3%; 5 years: 33.2%.  MWA+Sorafenib: 1 year: 86.2%; 3 years: 48.3%; 5 years: 34.6%.  **Recurrence:**  Resection: 5 years: 18.3%.  MWA+Sorafenib: 5 years: 38.3%.  Resection: Number of events: 11 (2 local recurrence, 9 distant recurrence).  MWA+Sorafenib: Number of events: 23 (7 local recurrence, 16 distant recurrence).  **Adverse events:**  Resection: Pain: 38 (63.3%); fever: 29 (48.3%); abdominal bleeding: 7 (11%); infection: 18 (30%).  MWA+Sorafenib: Pain: 14 (23.3%); fever: 15 (25%); abdominal bleeding: 2 (3.3%); infection: 1 (1.7%). |
| Zhang, 2007^35^  China | 133 patients with tumours ≤7cm; subgroup of 60 patients with tumours ≤3cm | RFA+PEI (29 patients with tumours ≤3cm) | RFA alone (31 patients with tumours ≤3cm) | **Overall survival:**  RFA+PEI: 1 year: 96.5%; 2 years: 92.9%; 3 years: 83.6%; 4 years: 77.4%; 5 years: 55.3%.  RFA alone: 1 year: 93.5%; 2 years: 80.7%; 3 years: 76.6%; 4 years: 70.2%; 5 years: 50.2%.  **Response:**  RFA+PEI: 52/66 (78.8%) had complete tumour ablation after one treatment, 14 (21.2%) had complete tumour ablation after a second treatment.  RFA alone: 48/67 (71.6%) had complete tumour ablation after one treatment, 18 (26.9%) had complete tumour ablation after a second treatment and 1 patient (1.5%) had viable tumour cells after 2 treatment sessions.  Tumour diameter was a significant prognostic factor for overall recurrence, intrahepatic recurrence and local recurrence. |
| Zhu, 2021 (protocol) ^36^  China | Ongoing RCT | RFA | Laparoscopic hepatectomy | N/A |
| Zou, 2017^37^  China | 74 patients with tumours ≤3cm | Laser ablation (n=35 patients) | RFA (n=39 patients) | **Response:**  Laser ablation: 31 (88.6%) had a complete response; 4 (11.4%) had a partial response.  RFA: 36 (92.3%) had a complete response; 3 (7.7%) had a partial response.  **Patient satisfaction:**  Self-made satisfaction questionnaire including intraoperative discomfort, post-operative therapy effects, adverse reactions and physical recovery. Maximum score of 100, 81-100 = great satisfaction, 61-80 = general satisfaction, <60 = dissatisfaction.  Laser ablation: Great satisfaction: 30 (85.7%), general satisfaction: 3 (8.6%), dissatisfaction: 2 (5.7%).  RFA: Great satisfaction: 25 (64.1%), general satisfaction: 2 (5.1%), dissatisfaction: 12 (30.8%).  **Adverse events:**  Laser ablation: Fever: 4 (11.4%); nausea: 17 (48.6%); vomiting: 10 (28.6%); diarrhoea: 1 (2.9%); abdominal pain: 26 (74.3%); skin rash: 1 (2.9%).  RFA: Fever: 5 (12.8%); nausea: 19 (48.7%); vomiting: 12 (30.8%); diarrhoea: 1 (2.6%); abdominal pain: 29 (74.4%); skin rash: 3 (7.7%). |

AEs, adverse events; CI, confidence interval; HCC, hepatocellular carcinoma; HR, hazard ratio; ICER, incremental cost-effectiveness ratio; ICU, intensive care unit; MWA, microwave ablation; PAI, percutaneous acid injection; PEI, percutaneous ethanol injection; RCT, randomised controlled trial; RFA, radiofrequency ablation; RR, relative risk; TACE, transarterial chemoembolization

## Risk of bias


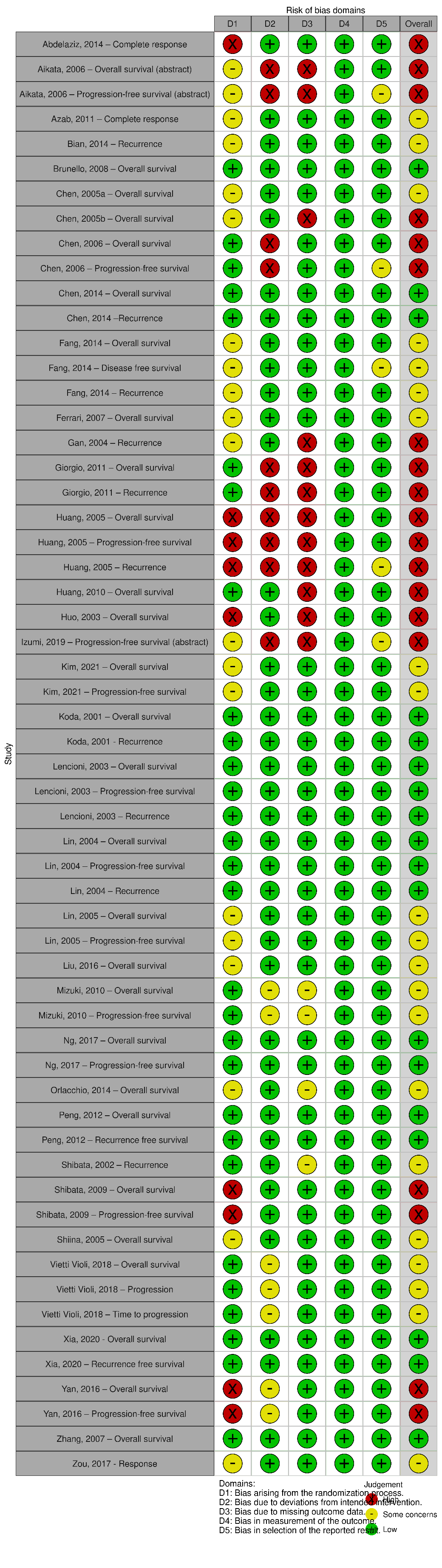
Figure A1: ****Risk of bias assessment results (created using**** ****the *robvis* tool(38))****


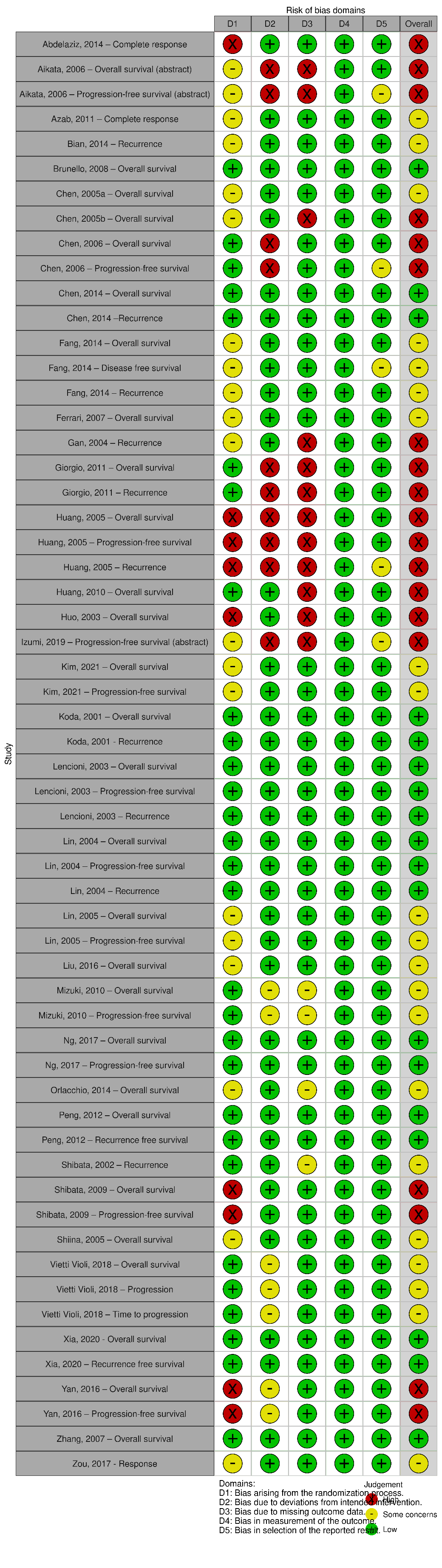


| **Judgement** | | **D1: Risk of bias arising from the randomisation process**  **D2: Risk of bias due to deviations from the intended intervention**  **D3: Risk of bias due to missing outcome data**  **D4: Risk of bias in measurement of the outcome**  **D5: Risk of bias in selection of the reported result** |
| --- | --- | --- |
| 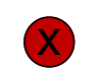 | **High** |  |
| 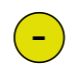 | **Some Concerns** |  |
| 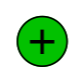 | **Low** |  |

# Ongoing trials

Published protocols

**Zhu F, Chang Q, Duan S, Leng W. Efficacy and safety of radiofrequency ablation versus laparoscopic hepatectomy for small hepatocellular carcinoma: a protocol for a randomized controlled trial. Medicine (Baltimore). 2021 2021;100:e23678.**

Single-centre (The Ninth People’s Hospital of Chongqing, China) RCT comparing radiofrequency ablation with laparoscopic hepatectomy for small HCC (three or fewer tumours ≤3cm in diameter).

Clinical trial register records

Four clinical trial register records (ClinicalTrials.gov) were identified in the searches:

**NCT04727307**

Multicentre RCT comparing atezolizumab + bevacizumab combined with radiofrequency ablation versus radiofrequency ablation alone for small HCC (1-3 nodules <3cm).

*Sponsor*: University Hospital, Montpellier, France.

*Actual study start date*: 26 January 2021.

*Estimated primary completion date*: January 2025.

*Estimated study completion date*: July 2027.

**NCT03790059**

Multicentre RCT comparing radiofrequency ablation combined with recombinant human adenovirus Type 5 (H101) injection versus radiofrequency ablation alone for small HCC (single lesion ≤3cm in diameter).

*Sponsor:* Southwest Hospital, China.

*Study start date:* October 2016.

*Estimated primary completion date*: September 2020.

*Estimated study completion date*: September 2020.

**NCT04235660**

Single-centre pilot RCT comparing Y90 radioembolisation versus stereotactic body radiation therapy for solitary early stage (≤3cm) HCC.

*Sponsor*: Indiana University, USA.

*Actual study start date*: 22 July 2020.

*Estimated primary completion date*: May 2024.

*Estimated study completion date*: May 2024.

**NCT04663035**

Clinical trial register record describing a single-centre RCT comparing ablation followed by tislelizumab (immunotherapy) versus ablation alone for early recurrent HCC.

*Sponsor*: Sun Yat-sen University, China.

*Actual study start date*: 21 December 2020.

*Estimated primary completion date*: December 2023.

*Estimated study completion date*: December 2025.

# Model fit parameters

Table A2: Model fit parameters for fixed- and random-effects models for network meta-analyses

|  | **Overall Survival**  (17 data-points) | **Progression-free Survival**  (7 data-points) | **Overall Recurrence**  (7 data-points) | **Local Recurrence**  (12 data-points) |
| --- | --- | --- | --- | --- |
| **DIC** | | | | |
| FE Model | 29.88 | 10.42 | 12.01 | 21.15 |
| RE Model: Half-Normal (0, 0.19^2^) Prior | 29.87 | 10.94 | 12.53 | 20.95 |
| RE Model: Half-Normal(0, 0.50^2^) Prior | 30.20 | 11.45 | 12.93 | 21.12 |
| **Total Residual Deviance^†^, Mean** | | | | |
| FE Model | 19.88 | 6.43 | 6.01 | 13.15 |
| RE Model: Half-Normal (0, 0.19^2^) Prior | 18.31 | 6.16 | 6.27 | 12.36 |
| RE Model: Half-Normal(0, 0.50^2^) Prior | 17.42 | 6.19 | 6.47 | 11.68 |
| **Between-study SD, Median (95% CrI)** | | | | |
| FE Model | -- | -- | -- | -- |
| RE Model: Half-Normal (0, 0.19^2^) Prior | 0.13 (0.01, 0.38) | 0.11 (0.01, 0.35) | 0.11 (0.005, 0.38) | 0.14 (0.01, 0.42) |
| RE Model: Half-Normal(0, 0.50^2^) Prior | 0.22 (0.01, 0.63) | 0.16 (0.01, 0.65) | 0.20 (0.01, 0.90) | 0.30 (0.02, 0.88) |

^†^ compared to the number of data-points

CrI, credible intervals; DIC, deviance information criteria; FE, fixed-effect; RE, random-effects; SD, standard deviation

# Inconsistency checks

Each data point’s contribution to the residual deviance in the unrelated mean effects (UME) inconsistency model was compared to its contribution to the residual deviance in the network meta-analysis (NMA) model. Potential inconsistency was assessed through node-splitting.

## Deviance contribution plots

Figure A2 shows the deviance contribution plot for overall survival. The points for two studies: Fang (2014) ^10^ (which compares resection and radiofrequency ablation [RFA]) and Shibata (2009) ^30^ (which compares RFA and RFA+ transarterial chemoembolization [TACE]) lie below the line of inequality.

**Figure A2: Deviance contribution plot for overall survival for the fixed-effect model**


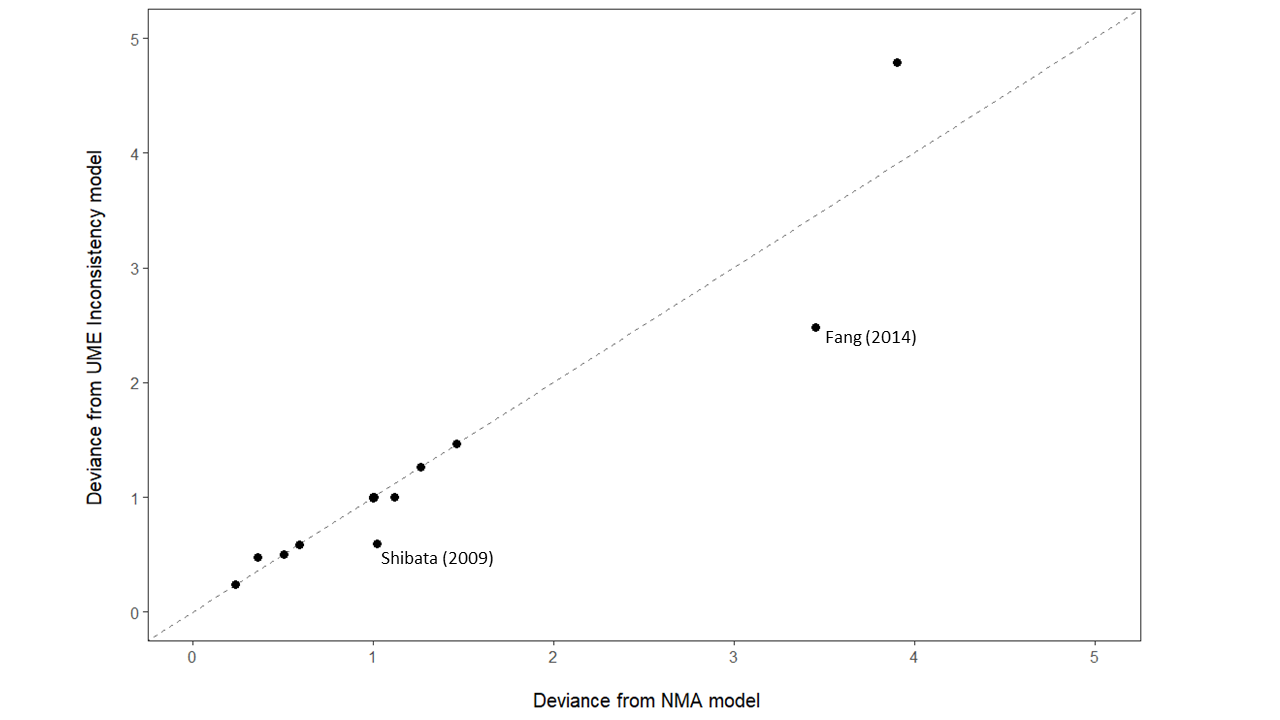


Source: Wade R, South E, Anwer S, Sharif-Hurst S, Harden M, Fulbright H, et al. Ablative and non-surgical therapies for early and very early hepatocellular carcinoma: a systematic review and network meta-analysis. *Health Technology Assessment (NIHR Journals Library)* (in press).

Figure A3 shows the deviance contribution plot for overall recurrence. No points in the plot lie below the line of inequality.

**Figure A3: Deviance contribution plot for overall recurrence for the fixed-effect model**


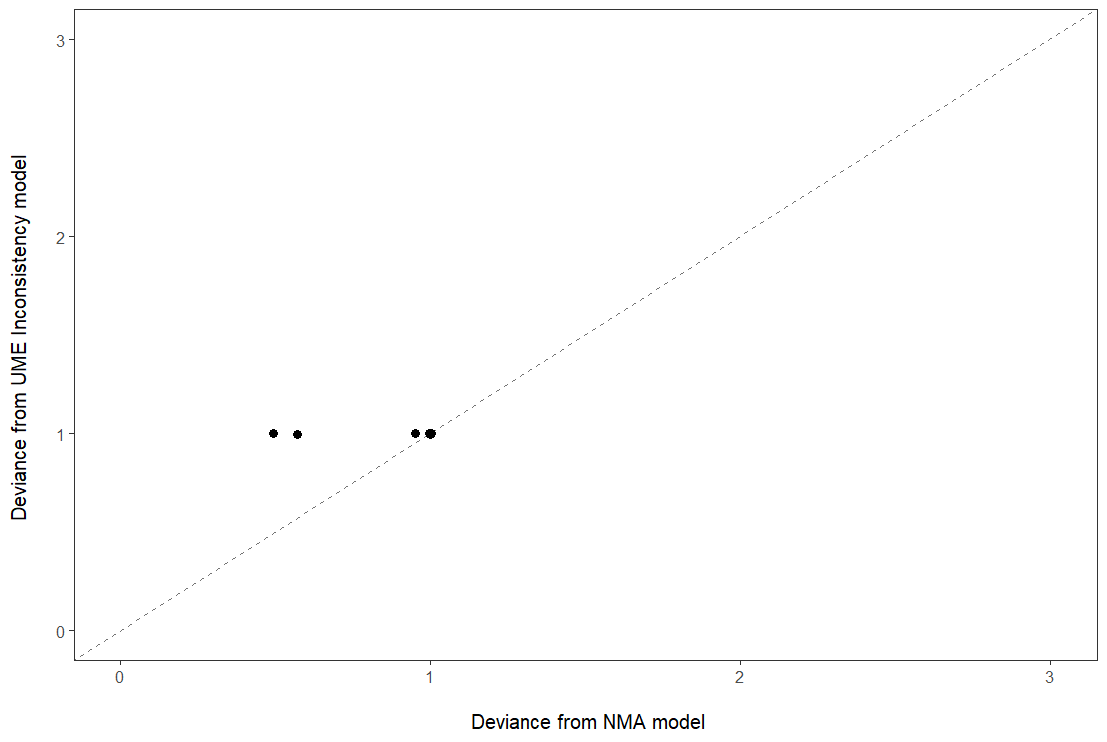


Source: Wade R, South E, Anwer S, Sharif-Hurst S, Harden M, Fulbright H, et al. Ablative and non-surgical therapies for early and very early hepatocellular carcinoma: a systematic review and network meta-analysis. *Health Technology Assessment (NIHR Journals Library)* (in press).

## Node splitting

Table A3 and Table A4 give the results of node-splitting for overall survival and overall recurrence, respectively. There was no evidence of inconsistency in either network.

**Table A3: Node-splitting results for overall survival for the fixed-effect model**

| **Comparison** | **LHRs, (95% CrI)** | | | ***p*** |
| --- | --- | --- | --- | --- |
|  | **Direct Model** | **Indirect Model** | **Network Model** |  |
| Resection vs. RFA | -0.044 (-0.445, 0.356) | -0.718 (-1.725, 0.291) | -0.137 (-0.511, 0.237) | 0.222 |
| RFA + TACE vs. RFA | -0.332 (-1.186, 0.531) | 0.346 (-0.328, 1.011) | 0.086 (-0.435, 0.614) | 0.223 |
| RFA + TACE vs. Resection | 0.389 (-0.147, 0.926) | -0.287 (-1.225, 0.657) | 0.224 (-0.238, 0.687) | 0.220 |

Negative valued LHRs favour the first named treatment
CrI, credible interval; LHR, log-hazard ratio; RFA, radiofrequency ablation; TACE, transarterial chemoembolization

**Table A4: Node-splitting results for overall recurrence for the fixed-effect model**

| **Comparison** | **LHRs, (95% CrI)** | | | ***p*** |
| --- | --- | --- | --- | --- |
|  | **Direct Model** | **Indirect Model** | **Network Model** |  |
| PEI vs. RFA | 0.177 (0.017, 0.340) | 0.134 (-0.571, 0.840) | 0.176 (0.018, 0.333) | 0.906 |
| Resection vs. RFA | -0.046 (-0.526, 0.433) | -0.005 (-0.548, 0.540) | -0.029 (-0.385, 0.331) | 0.911 |
| Resection vs. PEI | -0.182 (-0.702, 0.339) | -0.222 (-0.729, 0.273) | -0.204 (-0.565, 0.160) | 0.913 |

Negative valued LRRs favour the first named treatment
CrI: credible interval; LHR, log-relative risk; PEI, percutaneous ethanol injection; RFA, radiofrequency ablation

# NMA results for comparisons not involving RFA

Results of the fixed-effects model are presented below. The results of the random-effects model can be found in the full report. ^38^

## Overall survival

**Table A5: Hazard Ratios (HRs) for overall survival for fixed-effects model (non-RFA comparisons)**

| **Comparator** | **Baseline Intervention** | **Hazard Ratios (95% CrI)** |
| --- | --- | --- |
| PAI | PEI | 1.24 (0.69, 2.22) |
| Resection | PEI | **0.60 (0.39, 0.92)** |
| MWA | PEI | 0.64 (0.28, 1.45) |
| TACE + PEI | PEI | 0.70 (0.28, 1.73) |
| TACE + PAI | PEI | 1.29 (0.51, 3.22) |
| RFA + TACE | PEI | 0.75 (0.42, 1.33) |
| RFA + Iodine-125 | PEI | **0.35 (0.20, 0.58)** |
| RFA + PEI | PEI | 0.62 (0.20, 1.96) |
| Laser | PEI | 1.00 (0.54, 1.86) |
| Resection | PAI | 0.48 (0.23, 1.00) |
| MWA | PAI | 0.52 (0.19, 1.41) |
| TACE + PEI | PAI | 0.57 (0.19, 1.66) |
| TACE + PAI | PAI | 1.04 (0.51, 2.11) |
| RFA + TACE | PAI | 0.60 (0.27, 1.37) |
| RFA + Iodine-125 | PAI | **0.28 (0.13, 0.61)** |
| RFA + PEI | PAI | 0.50 (0.14, 1.82) |
| Laser | PAI | 0.81 (0.35, 1.89) |
| MWA | Resection | 1.07 (0.45, 2.56) |
| TACE + PEI | Resection | 1.17 (0.43, 3.19) |
| TACE + PAI | Resection | 2.15 (0.78, 5.92) |
| RFA + TACE | Resection | 1.25 (0.79, 1.99) |
| RFA + Iodine-125 | Resection | 0.58 (0.32, 1.05) |
| RFA + PEI | Resection | 1.03 (0.31, 3.39) |
| Laser | Resection | 1.68 (0.84, 3.32) |
| TACE + PEI | MWA | 1.09 (0.32, 3.68) |
| TACE + PAI | MWA | 2.01 (0.59, 6.82) |
| RFA + TACE | MWA | 1.17 (0.45, 3.00) |
| RFA + Iodine-125 | MWA | 0.54 (0.21, 1.34) |
| RFA + PEI | MWA | 0.96 (0.24, 3.81) |
| Laser | MWA | 1.56 (0.59, 4.12) |
| TACE + PAI | TACE + PEI | 1.84 (0.50, 6.66) |
| RFA + TACE | TACE + PEI | 1.07 (0.37, 3.12) |
| RFA + Iodine-125 | TACE + PEI | 0.49 (0.17, 1.40) |
| RFA + PEI | TACE + PEI | 0.88 (0.20, 3.81) |
| Laser | TACE + PEI | 1.43 (0.48, 4.26) |
| RFA + TACE | TACE + PAI | 0.58 (0.20, 1.71) |
| RFA + Iodine-125 | TACE + PAI | **0.27 (0.09, 0.77)** |
| RFA + PEI | TACE + PAI | 0.48 (0.11, 2.10) |
| Laser | TACE + PAI | 0.78 (0.26, 2.36) |
| RFA + Iodine-125 | RFA + TACE | **0.46 (0.23, 0.93)** |
| RFA + PEI | RFA + TACE | 0.83 (0.24, 2.87) |
| Laser | RFA + TACE | 1.34 (0.61, 2.91) |
| RFA + PEI | RFA + Iodine-125 | 1.80 (0.53, 6.11) |
| Laser | RFA + Iodine-125 | **2.91 (1.39, 6.11)** |
| Laser | RFA + PEI | 1.62 (0.46, 5.79) |

HRs less than one favour the comparator treatment. **Treatment comparisons in bold do not include the “null” effect**CrI, credible interval; MWA, microwave ablation; PEI, percutaneous ethanol injection; PAI, percutaneous acid injection; RFA, radiofrequency ablation; TACE, transarterial chemoembolization

## Progression-free survival

**Table A6: Hazard Ratios (HRs) for progression-free survival for the fixed-effects model (non-RFA comparisons)**

| **Comparator** | **Baseline Intervention** | **Hazard Ratios (95% CrI)** |
| --- | --- | --- |
| PAI | PEI | 1.19 (0.80, 1.78) |
| Resection | PEI | 0.74 (0.55, 1.01) |
| RFA + TACE | PEI | 0.58 (0.31, 1.09) |
| Resection | PAI | 0.62 (0.38, 1.02) |
| RFA + TACE | PAI | 0.49 (0.23, 1.02) |
| RFA + TACE | Resection | 0.79 (0.42, 1.49) |

HRs less than one favour the comparator treatment. **Treatment comparisons in bold do not include the “null” effect**CrI, credible interval; PEI, percutaneous ethanol injection; PAI, percutaneous acid injection; RFA, radiofrequency ablation; TACE, transarterial chemoembolization

## Overall recurrence

**Table A7: Relative Risks (RRs) for overall recurrence for the fixed-effects model (non-RFA comparisons)**

| **Comparator** | **Baseline Intervention** | **Relative Risks (95% CrI)** |
| --- | --- | --- |
| Resection | PEI | 0.82 (0.57, 1.17) |
| TACE + PEI | PEI | 1.18 (0.79, 1.77) |
| RFA + Iodine-125 | PEI | **0.58 (0.39, 0.86)** |
| MWA + Sorafenib | PEI | 1.70 (0.83, 3.50) |
| RFA + Systemic chemotherapy | PEI | 0.45 (0.16, 1.26) |
| TACE + PEI | Resection | 1.45 (0.84, 2.49) |
| RFA + Iodine-125 | Resection | 0.71 (0.43, 1.19) |
| MWA + Sorafenib | Resection | **2.09 (1.12, 3.89)** |
| RFA + Systemic chemotherapy | Resection | 0.55 (0.19, 1.62) |
| RFA + Iodine-125 | TACE + PEI | **0.49 (0.28, 0.86)** |
| MWA + Sorafenib | TACE + PEI | 1.44 (0.63, 3.29) |
| RFA + Systemic chemotherapy | TACE + PEI | 0.38 (0.13, 1.15) |
| MWA + Sorafenib | RFA + Iodine-125 | **2.93 (1.31, 6.56)** |
| RFA + Systemic chemotherapy | RFA + Iodine-125 | 0.77 (0.26, 2.28) |
| RFA + Systemic chemotherapy | MWA + Sorafenib | **0.26 (0.08, 0.92)** |

RRs less than one favour the comparator treatment. **Treatment comparisons in bold do not include the “null” effect**CrI, credible interval; MWA, microwave ablation; PEI, percutaneous ethanol injection; RFA, radiofrequency ablation; TACE, transarterial chemoembolization

## Local recurrence

**Table A8: Relative risks (RRs) for local recurrence for the fixed-effects model (non-RFA comparisons)**

| **Comparator** | **Baseline Intervention** | **Relative Risks (95% CrI)** |
| --- | --- | --- |
| PAI | PEI | 0.94 (0.56, 1.60) |
| MWA | PEI | 0.90 (0.34, 2.41) |
| TACE + PEI | PEI | 0.36 (0.13, 1.01) |
| RFA + TACE | PEI | 0.67 (0.26, 1.75) |
| Laser ablation | PEI | 1.66 (0.38, 7.36) |
| RFA + PEI | PEI | **0.33 (0.12, 0.94)** |
| High dose PEI | PEI | 0.90 (0.43, 1.88) |
| MWA | PAI | 0.96 (0.32, 2.79) |
| TACE + PEI | PAI | 0.39 (0.12, 1.21) |
| RFA + TACE | PAI | 0.71 (0.25, 2.05) |
| Laser ablation | PAI | 1.77 (0.37, 8.29) |
| RFA + PEI | PAI | 0.35 (0.11, 1.10) |
| High dose PEI | PAI | 0.95 (0.39, 2.33) |
| TACE + PEI | MWA | 0.40 (0.10, 1.65) |
| RFA + TACE | MWA | 0.75 (0.22, 2.59) |
| Laser ablation | MWA | 1.85 (0.34, 10.04) |
| RFA + PEI | MWA | 0.37 (0.10, 1.37) |
| High dose PEI | MWA | 1.00 (0.30, 3.28) |
| RFA + TACE | TACE + PEI | 1.85 (0.46, 7.45) |
| Laser ablation | TACE + PEI | 4.58 (0.76, 27.67) |
| RFA + PEI | TACE + PEI | 0.92 (0.21, 3.92) |
| High dose PEI | TACE + PEI | 2.47 (0.70, 8.68) |
| Laser ablation | RFA + TACE | 2.47 (0.46, 13.18) |
| RFA + PEI | RFA + TACE | 0.50 (0.14, 1.79) |
| High dose PEI | RFA + TACE | 1.34 (0.42, 4.26) |
| RFA + PEI | Laser ablation | 0.20 (0.04, 1.12) |
| High dose PEI | Laser ablation | 0.54 (0.11, 2.75) |
| High dose PEI | RFA + PEI | 2.70 (0.78, 9.27) |

RRs less than one favour the comparator treatment. **Treatment comparisons in bold do not include the “null” effect**CrI, credible interval; MWA, microwave ablation; PEI, percutaneous ethanol injection; PAI, percutaneous acid injection; RFA, radiofrequency ablation; TACE, transarterial chemoembolization

# Mean and median treatment ranks

## Overall survival

Table A9: Mean and median treatment ranks for the fixed-effect model, with corresponding 95% CrIs for overall survival, sorted by mean rank out of 11 treatments.

| **Treatments** | **Mean Rank** | **Median Rank** | **95% CrI for the rank** |
| --- | --- | --- | --- |
| RFA + Iodine-125 | 1.42 | 1 | (1.00, 3.00) |
| Resection | 3.84 | 4 | (2.00, 7.00) |
| MWA | 4.81 | 4 | (1.00, 11.00) |
| RFA + PEI | 4.82 | 4 | (1.00, 11.00) |
| RFA | 4.98 | 5 | (3.00, 7.00) |
| TACE + PEI | 5.42 | 5 | (1.00, 11.00) |
| RFA + TACE | 5.90 | 6 | (2.00, 10.00) |
| Laser | 8.07 | 8 | (3.00, 11.00) |
| PEI | 8.28 | 8 | (6.00, 11.00) |
| TACE + PAI | 9.11 | 10 | (3.00, 11.00) |
| PAI | 9.34 | 10 | (5.00, 11.00) |

CrI: credible intervals**;** MWA, microwave ablation; PAI, percutaneous acid injection; PEI, percutaneous ethanol injection; RFA, radiofrequency ablation; TACE, transarterial chemoembolization

Source: Wade R, South E, Anwer S, Sharif-Hurst S, Harden M, Fulbright H, et al. Ablative and non-surgical therapies for early and very early hepatocellular carcinoma: a systematic review and network meta-analysis. *Health Technology Assessment (NIHR Journals Library)* (in press).

## Progression-free survival

Table A10: Mean and median ranks for the fixed-effect model, with the corresponding 95% CrIs for progression-free survival, sorted by mean rank out of 5 treatments.

| **Treatments** | **Mean Rank** | **Median Rank** | **95% CrI for the rank** |
| --- | --- | --- | --- |
| RFA + TACE | 1.53 | 1 | (1.00, 4.00) |
| RFA | 2.24 | 2 | (1.00, 3.00) |
| Resection | 2.38 | 2 | (1.00, 4.00) |
| PEI | 4.12 | 4 | (3.00, 5.00) |
| PAI | 4.73 | 5 | (3.00, 5.00) |

CrI: credible intervals**;** PAI, percutaneous acid injection; PEI, percutaneous ethanol injection; RFA, radiofrequency ablation; TACE, transarterial chemoembolization

Source: Wade R, South E, Anwer S, Sharif-Hurst S, Harden M, Fulbright H, et al. Ablative and non-surgical therapies for early and very early hepatocellular carcinoma: a systematic review and network meta-analysis. *Health Technology Assessment (NIHR Journals Library)* (in press).

## Overall recurrence

Table A11: Mean and median ranks for the fixed-effect model, with corresponding 95% CrIs for overall recurrence, sorted by mean rank, out of 7 treatments.

| **Treatments** | **Mean Rank** | **Median Rank** | **95% CrI for the rank** |
| --- | --- | --- | --- |
| RFA + Systemic Chemotherapy | 1.70 | 1 | (1.00, 6.00) |
| RFA + Iodine-125 | 1.81 | 2 | (1.00, 3.00) |
| Resection | 3.44 | 3 | (2.00, 6.00) |
| RFA | 3.52 | 4 | (2.00, 5.00) |
| PEI | 5.06 | 5 | (4.00, 6.00) |
| TACE + PEI | 5.79 | 6 | (3.00, 7.00) |
| MWA + Sorafenib | 6.67 | 7 | (4.00, 7.00) |

CrI: credible intervals**;** MWA, microwave ablation; PEI, percutaneous ethanol injection; RFA, radiofrequency ablation; TACE, transarterial chemoembolization

Source: Wade R, South E, Anwer S, Sharif-Hurst S, Harden M, Fulbright H, et al. Ablative and non-surgical therapies for early and very early hepatocellular carcinoma: a systematic review and network meta-analysis. *Health Technology Assessment (NIHR Journals Library)* (in press).

## Local recurrence

Table A12: Mean and median ranks for the fixed-effect model, with corresponding 95% CrIs for local recurrence, sorted by mean rank, out of 9 treatments

| **Treatments** | **Mean Rank** | **Median Rank** | **95% CrI of the rank** |
| --- | --- | --- | --- |
| RFA + PEI | 1.96 | 2 | (1.00, 6.00) |
| TACE + PEI | 2.27 | 2 | (1.00, 7.00) |
| RFA | 3.33 | 3 | (2.00, 5.00) |
| RFA + TACE | 4.59 | 4 | (1.00, 9.00) |
| MWA | 5.98 | 6 | (2.00, 9.00) |
| High dose PEI | 6.01 | 6 | (2.00, 9.00) |
| PAI | 6.33 | 6 | (3.00, 9.00) |
| PEI | 6.78 | 7 | (4.00, 9.00) |
| Laser ablation | 7.75 | 9 | (2.00, 9.00) |

CrI: credible intervals**;** MWA, microwave ablation; PAI, percutaneous acid injection; PEI, percutaneous ethanol injection; RFA, radiofrequency ablation; TACE, transarterial chemoembolization

Source: Wade R, South E, Anwer S, Sharif-Hurst S, Harden M, Fulbright H, et al. Ablative and non-surgical therapies for early and very early hepatocellular carcinoma: a systematic review and network meta-analysis. *Health Technology Assessment (NIHR Journals Library)* (in press).

# Threshold analysis results for overall survival

Figure A4: Forest plot for threshold analysis results for overall survival


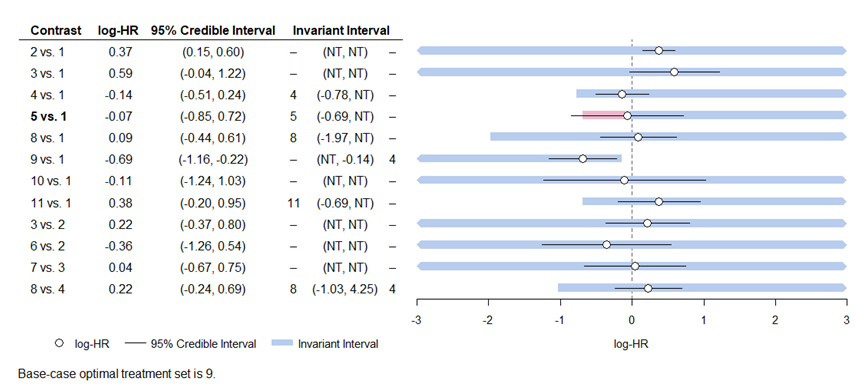


The blue shaded bars represent the invariant interval; where the bars are shown in red, credible intervals for the comparison extend beyond the invariant interval. **Treatment Codes:** 1: RFA, 2: PEI, 3: PAI, 4: Resection, 5: MWA, 6: TACE + PEI, 7: TACE + PAI, 8: RFA + TACE, 9: RFA + Iodine-125, 10: RFA + PEI, 11: Laser. The optimum treatment for this analysis was RFA + Iodine-125.

Source: Wade R, South E, Anwer S, Sharif-Hurst S, Harden M, Fulbright H, et al. Ablative and non-surgical therapies for early and very early hepatocellular carcinoma: a systematic review and network meta-analysis. *Health Technology Assessment (NIHR Journals Library)* (in press).

References

1. Abdelaziz A, Elbaz T, Shousha HI, Mahmoud S, Ibrahim M, Abdelmaksoud A*, et al.* Efficacy and survival analysis of percutaneous radiofrequency versus microwave ablation for hepatocellular carcinoma: an Egyptian multidisciplinary clinic experience. *Surg Endosc* 2014;**28**:3429-34. https://dx.doi.org/10.1007/s00464-014-3617-4

2. Aikata H, Shirakawa H, Takaki S, Uka K, Miki D, Yamashina K. Radiofrequency ablation combined with transcatheter arterial chemoembolization for small hepatocellular carcinomas. *Hepatology* 2006;**44**:494A.

3. Azab M, Zaki S, El-Shetey AG, Abdel-Moty MF, Alnoomani NM, Gomaa AA*, et al.* Radiofrequency ablation combined with percutaneous ethanol injection in patients with hepatocellular carcinoma. *Arab J Gastroenterol* 2011;**12**:113-8. https://dx.doi.org/10.1016/j.ajg.2011.07.005

4. Bian H, Zheng JS, Nan G, Li R, Chen C, Hu CX*, et al.* Randomized trial of [^131^I] metuximab in treatment of hepatocellular carcinoma after percutaneous radiofrequency ablation. *J Natl Cancer Inst* 2014;**106**:dju239. https://dx.doi.org/10.1093/jnci/dju239

5. Brunello F, Veltri A, Carucci P, Pagano E, Ciccone G, Moretto P*, et al.* Radiofrequency ablation versus ethanol injection for early hepatocellular carcinoma: a randomized controlled trial. *Scand J Gastroenterol* 2008;**43**:727-35. https://dx.doi.org/10.1080/00365520701885481

6. Chen MS, Li JQ, Liang HH, Lin XJ, Guo RP, Zheng Y*, et al.* [Comparison of effects of percutaneous radiofrequency ablation and surgical resection on small hepatocellular carcinoma]. *Zhonghua Yi Xue Za Zhi* 2005;**85**:80-3.

7. Chen MS, Zhang YJ, Li JQ, Liang HH, Zhang YQ, Zheng Y. [Randomized clinical trial of percutaneous radiofrequency ablation plus absolute ethanol injection compared with radiofrequency ablation alone for small hepatocellular carcinoma]. *Zhonghua Zhong Liu Za Zhi* 2005;**27**:623-5.

8. Chen MS, Li JQ, Zheng Y, Guo RP, Liang HH, Zhang YQ*, et al.* A prospective randomized trial comparing percutaneous local ablative therapy and partial hepatectomy for small hepatocellular carcinoma. *Ann Surg* 2006;**243**:321-8.

9. Chen K, Chen G, Wang H, Li H, Xiao J, Duan X*, et al.* Increased survival in hepatocellular carcinoma with iodine-125 implantation plus radiofrequency ablation: a prospective randomized controlled trial. *J Hepatol* 2014;**61**:1304-11. https://dx.doi.org/10.1016/j.jhep.2014.07.026

10. Fang Y, Chen W, Liang X, Li D, Lou H, Chen R*, et al.* Comparison of long-term effectiveness and complications of radiofrequency ablation with hepatectomy for small hepatocellular carcinoma. *J Gastroenterol Hepatol* 2014;**29**:193-200. https://dx.doi.org/10.1111/jgh.12441

11. Feng K, Yan J, Li X, Xia F, Ma K, Wang S*, et al.* A randomized controlled trial of radiofrequency ablation and surgical resection in the treatment of small hepatocellular carcinoma. *J Hepatol* 2012;**57**:794-802. https://dx.doi.org/10.1016/j.jhep.2012.05.007

12. Ferrari FS, Megliola A, Scorzelli A, Stella A, Vigni F, Drudi FM*, et al.* Treatment of small HCC through radiofrequency ablation and laser ablation. Comparison of techniques and long-term results. *Radiol Med* 2007;**112**:377-93. https://doi.org/10.1007/s11547-007-0148-2

13. Gan YH, Yie SL, Ren ZG, Xia JL, Zhang BH, Wang YH*, et al.* [Prospective randomized trial of RFA and chemotherapy for unresectable small hepatocellular carcinoma]. *Zhonghua Zhong Liu Za Zhi* 2004;**26**:496-8.

14. Giorgio A, Di Sarno A, De Stefano G, Scognamiglio U, Farella N, Mariniello A*, et al.* Percutaneous radiofrequency ablation of hepatocellular carcinoma compared to percutaneous ethanol injection in treatment of cirrhotic patients: an Italian randomized controlled trial. *Anticancer Res* 2011;**31**:2291-5.

15. Huang GT, Lee PH, Tsang YM, Lai MY, Yang PM, Hu RH*, et al.* Percutaneous ethanol injection versus surgical resection for the treatment of small hepatocellular carcinoma: a prospective study. *Ann Surg* 2005;**242**:36-42. https://doi.org/10.1097/01.sla.0000167925.90380.fe

16. Huang J, Yan L, Cheng Z, Wu H, Du L, Wang J*, et al.* A randomized trial comparing radiofrequency ablation and surgical resection for HCC conforming to the Milan criteria. *Ann Surg* 2010;**252**:903-12. https://dx.doi.org/10.1097/SLA.0b013e3181efc656

17. Huo TI, Huang YH, Wu JC, Chiang JH, Lee PC, Chang FY*, et al.* Sequential transarterial chemoembolization and percutaneous acetic acid injection therapy versus repeated percutaneous acetic acid injection for unresectable hepatocellular carcinoma: a prospective study. *Ann Oncol* 2003;**14**:1648-53.

18. Izumi N, Hasegawa K, Nishioka Y, Takayama T, Yamanaka N, Kudo M*, et al.* A multicenter randomized controlled trial to evaluate the efficacy of surgery vs. radiofrequency ablation for small hepatocellular carcinoma (SURF trial). *J Clin Oncol* 2019;**37**. https://doi.org/10.1200/JCO.2019.37.15_suppl.4002

19. Kim TH, Koh YH, Kim BH, Kim MJ, Lee JH, Park B*, et al.* Proton beam radiotherapy vs. radiofrequency ablation for recurrent hepatocellular carcinoma: a randomized phase III trial. *J Hepatol* 2021;**74**:603-12. https://doi.org/10.1016/j.jhep.2020.09.026

20. Koda M, Murawaki Y, Mitsuda A, Oyama K, Okamoto K, Idobe Y*, et al.* Combination therapy with transcatheter arterial chemoembolization and percutaneous ethanol injection compared with percutaneous ethanol injection alone for patients with small hepatocellular carcinoma: a randomized control study. *Cancer* 2001;**92**:1516-24. https://doi.org/10.1002/1097-0142(20010915)92:6%3C1516::AID-CNCR1477%3E3.0.CO;2-I

21. Lencioni RA, Allgaier HP, Cioni D, Olschewski M, Deibert P, Crocetti L*, et al.* Small hepatocellular carcinoma in cirrhosis: randomized comparison of radio-frequency thermal ablation versus percutaneous ethanol injection. *Radiology* 2003;**228**:235-40. https://doi.org/10.1148/radiol.2281020718

22. Lin SM, Lin CJ, Lin CC, Hsu CW, Chen YC. Radiofrequency ablation improves prognosis compared with ethanol injection for hepatocellular carcinoma < or =4 cm. *Gastroenterology* 2004;**127**:1714-23. https://doi.org/10.1053/j.gastro.2004.09.003

23. Lin SM, Lin CJ, Lin CC, Hsu CW, Chen YC. Randomised controlled trial comparing percutaneous radiofrequency thermal ablation, percutaneous ethanol injection, and percutaneous acetic acid injection to treat hepatocellular carcinoma of 3 cm or less. *Gut* 2005;**54**:1151-6. http://dx.doi.org/10.1136/gut.2004.045203

24. Liu H, Wang ZG, Fu SY, Li AJ, Pan ZY, Zhou WP*, et al.* Randomized clinical trial of chemoembolization plus radiofrequency ablation versus partial hepatectomy for hepatocellular carcinoma within the Milan criteria. *Br J Surg* 2016;**103**:348-56. https://dx.doi.org/10.1002/bjs.10061

25. Mizuki A, Tatemichi M, Tsukada N, Nagamatsu R, Kawaguchi M, Itoshima T*, et al.* Addition of transcatheter arterial chemoembolization decreased local recurrence but had no survival benefit to percutaneous ethanol injection therapy for patients with small hepatocellular carcinoma: a multicenter randomized control study. *Oncol Lett* 2010;**1**:855-9. https://doi.org/10.3892/ol_00000151

26. Ng KK, Chok KS, Chan AC, Cheung TT, Wong TC, Fung JY*, et al.* Randomized clinical trial of hepatic resection versus radiofrequency ablation for early-stage hepatocellular carcinoma. *Br J Surg* 2017;**104**:1775-84. https://dx.doi.org/10.1002/bjs.10677

27. Orlacchio A, Bolacchi F, Chegai F, Bergamini A, Costanzo E, Del Giudice C*, et al.* Comparative evaluation of percutaneous laser and radiofrequency ablation in patients with HCC smaller than 4 cm. *Radiol Med* 2014;**119**:298-308. https://dx.doi.org/10.1007/s11547-013-0339-y

28. Peng ZW, Zhang YJ, Liang HH, Lin XJ, Guo RP, Chen MS. Recurrent hepatocellular carcinoma treated with sequential transcatheter arterial chemoembolization and RF ablation versus RF ablation alone: a prospective randomized trial. *Radiology* 2012;**262**:689-700. https://doi.org/10.1148/radiol.11110637

29. Shibata T, Iimuro Y, Yamamoto Y, Maetani Y, Ametani F, Itoh K*, et al.* Small hepatocellular carcinoma: comparison of radio-frequency ablation and percutaneous microwave coagulation therapy. *Radiology* 2002;**223**:331-7. https://doi.org/10.1148/radiol.2232010775

30. Shibata T, Isoda H, Hirokawa Y, Arizono S, Shimada K, Togashi K. Small hepatocellular carcinoma: is radiofrequency ablation combined with transcatheter arterial chemoembolization more effective than radiofrequency ablation alone for treatment? *Radiology* 2009;**252**:905-13. https://dx.doi.org/10.1148/radiol.2523081676

31. Shiina S, Teratani T, Obi S, Sato S, Tateishi R, Fujishima T*, et al.* A randomized controlled trial of radiofrequency ablation with ethanol injection for small hepatocellular carcinoma. *Gastroenterology* 2005;**129**:122-30. https://doi.org/10.1053/j.gastro.2005.04.009

32. Vietti V, Duran R, Guiu B, Cercueil JP, Aube C, Digklia A*, et al.* Efficacy of microwave ablation versus radiofrequency ablation for the treatment of hepatocellular carcinoma in patients with chronic liver disease: a randomised controlled phase 2 trial. *Lancet Gastroenterol Hepatol* 2018;**3**:317-25. https://dx.doi.org/10.1016/S2468-1253(18)30029-3

33. Xia Y, Li J, Liu G, Wang K, Qian G, Lu Z*, et al.* Long-term effects of repeat hepatectomy vs percutaneous radiofrequency ablation among patients with recurrent hepatocellular carcinoma: a randomized clinical trial. *JAMA Oncol* 2020;**6**:255-63. https://dx.doi.org/10.1001/jamaoncol.2019.4477

34. Yan SY, Zhang Y, Sun C, Cao HX, Li GM, Wang YQ*, et al.* The clinical effect and relevant mechanism of combined sorafenib and radiofrequency ablation in the treatment of early small hepatocellular carcinoma. *Oncol Lett* 2016;**12**:951-5. https://doi.org/10.3892/ol.2016.4694

35. Zhang YJ, Liang HH, Chen MS, Guo RP, Li JQ, Zheng Y*, et al.* Hepatocellular carcinoma treated with radiofrequency ablation with or without ethanol injection: a prospective randomized trial. *Radiology* 2007;**244**:599-607. https://doi.org/10.1148/radiol.2442060826

36. Zhu F, Chang Q, Duan S, Leng W. Efficacy and safety of radiofrequency ablation versus laparoscopic hepatectomy for small hepatocellular carcinoma: a protocol for a randomized controlled trial. *Medicine (Baltimore)* 2021;**100**:e23678. https://dx.doi.org/10.1097/MD.0000000000023678

37. Zou D, Pan D, Deng L, Mao W, Zhang F, Miao J. Comparison of ultrasound-guided laser ablation and radiofrequency ablation in the treatment of small hepatocellular carcinoma. *Int J Clin Exp Med* 2017;**10**:9562-8.

38. Wade R, South E, Anwer S, Sharif-Hurst S, Harden M, Fulbright H*, et al.* Ablative and non-surgical therapies for early and very early hepatocellular carcinoma: a systematic review and network meta-analysis. *Health Technol Assess* (in press).
